# Dispersion of the HIV-1 Epidemic in Men Who Have Sex with Men in the Netherlands: A Combined Mathematical Model and Phylogenetic Analysis

**DOI:** 10.1371/journal.pmed.1001898

**Published:** 2015-11-03

**Authors:** Daniela Bezemer, Anne Cori, Oliver Ratmann, Ard van Sighem, Hillegonda S. Hermanides, Bas E. Dutilh, Luuk Gras, Nuno Rodrigues Faria, Rob van den Hengel, Ashley J. Duits, Peter Reiss, Frank de Wolf, Christophe Fraser, ATHENA observational cohort

**Affiliations:** 1 HIV Monitoring Foundation, Amsterdam, the Netherlands; 2 Medical Research Council Centre for Outbreak Analysis and Modelling, Department of Infectious Disease Epidemiology, School of Public Health, Imperial College London, London, United Kingdom; 3 Red Cross Blood Bank Foundation, Willemstad, Curaçao; 4 Centre for Molecular and Biomolecular Informatics, Nijmegen Centre for Molecular Life Sciences, Radboud University Medical Centre, Nijmegen, the Netherlands; 5 Department of Marine Biology, Institute of Biology, Federal University of Rio de Janeiro, Rio de Janeiro, Brazil; 6 Theoretical Biology and Bioinformatics, Utrecht University, Utrecht, the Netherlands; 7 Department of Zoology, University of Oxford, Oxford, United Kingdom; 8 Department of Global Health, Academic Medical Center, Amsterdam, the Netherlands; 9 Amsterdam Institute for Global Health and Development, Amsterdam, the Netherlands; Johns Hopkins University, UNITED STATES

## Abstract

**Background:**

The HIV-1 subtype B epidemic amongst men who have sex with men (MSM) is resurgent in many countries despite the widespread use of effective combination antiretroviral therapy (cART). In this combined mathematical and phylogenetic study of observational data, we aimed to find out the extent to which the resurgent epidemic is the result of newly introduced strains or of growth of already circulating strains.

**Methods and Findings:**

As of November 2011, the ATHENA observational HIV cohort of all patients in care in the Netherlands since 1996 included HIV-1 subtype B *polymerase* sequences from 5,852 patients. Patients who were diagnosed between 1981 and 1995 were included in the cohort if they were still alive in 1996. The ten most similar sequences to each ATHENA sequence were selected from the Los Alamos HIV Sequence Database, and a phylogenetic tree was created of a total of 8,320 sequences. Large transmission clusters that included ≥10 ATHENA sequences were selected, with a local support value ≥ 0.9 and median pairwise patristic distance below the fifth percentile of distances in the whole tree. Time-varying reproduction numbers of the large MSM-majority clusters were estimated through mathematical modeling. We identified 106 large transmission clusters, including 3,061 (52%) ATHENA and 652 Los Alamos sequences. Half of the HIV sequences from MSM registered in the cohort in the Netherlands (2,128 of 4,288) were included in 91 large MSM-majority clusters. Strikingly, at least 54 (59%) of these 91 MSM-majority clusters were already circulating before 1996, when cART was introduced, and have persisted to the present. Overall, 1,226 (35%) of the 3,460 diagnoses among MSM since 1996 were found in these 54 long-standing clusters. The reproduction numbers of all large MSM-majority clusters were around the epidemic threshold value of one over the whole study period. A tendency towards higher numbers was visible in recent years, especially in the more recently introduced clusters. The mean age of MSM at diagnosis increased by 0.45 years/year within clusters, but new clusters appeared with lower mean age. Major strengths of this study are the high proportion of HIV-positive MSM with a sequence in this study and the combined application of phylogenetic and modeling approaches. Main limitations are the assumption that the sampled population is representative of the overall HIV-positive population and the assumption that the diagnosis interval distribution is similar between clusters.

**Conclusions:**

The resurgent HIV epidemic amongst MSM in the Netherlands is driven by several large, persistent, self-sustaining, and, in many cases, growing sub-epidemics shifting towards new generations of MSM. Many of the sub-epidemics have been present since the early epidemic, to which new sub-epidemics are being added.

## Abstract 2

Daniela Bezemer and colleagues investigate the extent to which the resurgent HIV epidemic in the Netherlands is the result of newly introduced strains, or of growth of already circulating strains.

## Editors' Summary

**Background:**

Since the first recorded case of AIDS in 1981, the number of people infected with HIV, the virus that causes AIDS, has risen steadily. Now, three and a half decades later, about 35 million people (more than half of whom are women) are infected with HIV, the virus that causes AIDS. HIV is most often spread by having unprotected sex with an infected partner, and, globally, most sexual transmission of HIV occurs during heterosexual sex. Nevertheless, many new HIV infections still occur in men who have sex with men (MSM; homosexual, bisexual, and transgender men, and heterosexual men who sometimes have sex with men), and, in some countries, HIV/AIDS still predominantly affects the MSM community. In the US, for example, 78% of new HIV infections occurred among MSM in 2010 although MSM represent only 4% of the total population, and, in 2011, 54% of all people living with HIV were MSM. Indeed, despite HIV-positive individuals being diagnosed earlier these days and having access to effective combination antiretroviral therapy (cART), which both halts disease progression and reduces the risk of HIV transmission, the HIV epidemic among MSM is resurgent (growing again) in many Western countries.

**Why Was This Study Done?:**

To control this resurgent epidemic, it is important to know as much as possible about HIV transmission among MSM so that effective prevention strategies can be designed. Here, the researchers use phylogenetic analysis and mathematical modeling to ask whether the introduction of new strains or the spread of already circulating strains is responsible for the resurgent HIV-1 subtype B epidemic occurring among MSM in the Netherlands. Viral phylogenetic analysis infers evolutionary relationships between viral strains by examining their genetic relatedness and can be used to identify HIV transmission clusters. HIV-1 viruses are classified into subtypes based on their genetic sequence and geographical distribution. HIV-1 subtype B is a common subtype that is found in west and central Europe, the Americas, and several other regions.

**What Did the Researchers Do and Find?:**

The researchers built a phylogenetic tree for the HIV epidemic in MSM in the Netherlands by analyzing HIV-1 subtype B *polymerase* gene sequences found in 5,852 participants (73% of whom were MSM) in the ATHENA cohort, an observational cohort of all HIV-1-infected patients in care in the Netherlands since 1996 (when cART became available). Examination of this tree identified 106 large transmission clusters (groups of ten or more closely related subtype B HIV-1 strains). Half of the HIV-1 *polymerase* sequences from HIV-1-positive MSM registered in the ATHENA cohort in the Netherlands were included in 91 MSM-majority clusters: large transmission clusters in which more than half the related sequences originated from MSM. At least 54 of the MSM-majority clusters were circulating before 1996 and have persisted until the present day. Moreover, about a third of new HIV infections diagnosed among MSM since 1996 involve viruses included in these long-lived clusters. The researchers then used mathematical modeling to estimate that the effective reproduction number (the number of secondary infections per primary infection) for all the MSM-majority clusters was around one for the whole study period. Thus, these clusters were self-sustaining and not contracting. Notably, MSM-majority clusters (particularly the newer clusters) tended to have higher reproduction numbers in recent years. Moreover, although the average age at diagnosis within each of the MSM-majority clusters increased over the study period at a rate of 0.45 years/year, the average age at diagnosis was lower at initiation of new clusters and only increased by 0.28 years/year.

**What Do These Findings Mean?:**

These findings suggest that several large, persistent, and self-sustaining sub-epidemics, many of which have been present since early in the AIDS epidemic, are driving the resurgent HIV epidemic among MSM in the Netherlands, despite the widespread availability of treatment, increasing rates of diagnosis, and earlier treatment initiation. Importantly, however, these findings also suggest that some sub-epidemics have emerged more recently and that some sub-epidemics, particularly the newer ones, are growing and may be preferentially affecting younger MSM. The accuracy of these findings may be limited by some aspects of the study. For example, the reproduction number estimates assume that the time from diagnosis of a case to the diagnoses of secondary cases is similar across clusters. Nevertheless, the new insights provided by this study should help guide the development of strategies to curb the resurgent HIV epidemic that is currently affecting MSM in the Netherlands and elsewhere.

**Additional Information:**

This list of resources contains links that can be accessed when viewing the PDF on a device or via the online version of the article at http://dx.doi.org/10.1371/journal.pmed.1001898.

- The US Centers for Disease Control and Prevention provides information on all aspects of HIV/AIDS, including information on HIV/AIDS among MSM (in English and Spanish)
- NAM/aidsmap provides basic information about HIV/AIDS and summaries of recent research findings, including information on HIV and MSM and personal stories from MSM living with HIV
- Information is available from Avert, an international AIDS charity on many aspects of HIV/AIDS, including MSM and HIV; Avert also provides personal stories about living with HIV/AIDS
- The World Health Organization provides information on all aspects of HIV/AIDS, including HIV/AIDS and MSM (in several languages)
- The UNAIDS Fast-Track Strategy to End the AIDS Epidemic by 2030 provides up-to-date information about the AIDS epidemic and efforts to halt it
- A 2011 World Bank Report *The Global HIV Epidemics among Men Who Have Sex with Men* is available
- A *PLOS Computational Biology* Topic Page (a review article that is a published copy of record of a dynamic version of an article in Wikipedia) about viral phylodynamics is available

## Introduction

The HIV-1 epidemic amongst men who have sex with men (MSM) is resurgent in many Western countries [1–5]. This may seem paradoxical, as HIV-positive men are being diagnosed at an increasingly earlier stage of HIV infection, and, since 1996, most diagnosed men take effective combination antiretroviral therapy (cART). Not only does successful therapy halt disease progression [6], but successful suppression of virus replication also reduces the risk of transmitting HIV [7,8]. With the introduction of cART, however, there has been an increase in unprotected sex in many countries. For the Netherlands, this was first demonstrated using a mathematical model that included disease progression to analyze national data from the ATHENA cohort on HIV and AIDS diagnoses and treatment success and failure [1,2]. These findings were later confirmed by analysis of behavioral data from the Amsterdam Cohort Studies, which found that sexual risk behavior increased in a manner similar to model predictions [9,10]. The resulting reproduction number for the epidemic amongst MSM was estimated to be around the epidemic threshold of one [2]. In the period 2007–2010, 2,928 new HIV diagnoses were registered amongst MSM in the Netherlands, very similar to earlier predictions assuming no reductions in risk behavior [2].

In the current study, we aimed to gain more insight into the transmission clusters that constitute this mainly HIV-1 subtype B epidemic amongst MSM over time [11]. Similarly to an earlier Swiss study, we used phylogenetic analysis of extensive HIV-1 subtype B *polymerase* (*pol*) sequence data to identify large transmission clusters [12]. The authors of the Swiss study subsequently estimated a reproduction number for each sub-epidemic directly from the sequence data [13,14]. Here, we also analyze the viral transmission dynamics over time within the different clusters. However, we applied a mathematical model to the estimated incidence of diagnoses within each cluster, using the distribution of time between diagnoses of index cases and secondary cases derived from a previous study [2,15]. Our analysis estimates the time-varying reproduction numbers of the large MSM-majority clusters that together drive the overall dynamics of the epidemic. This will clarify the extent to which the resurgent epidemic is the result of newly introduced strains or of growth of already circulating strains.

## Methods

**Fig 1:**
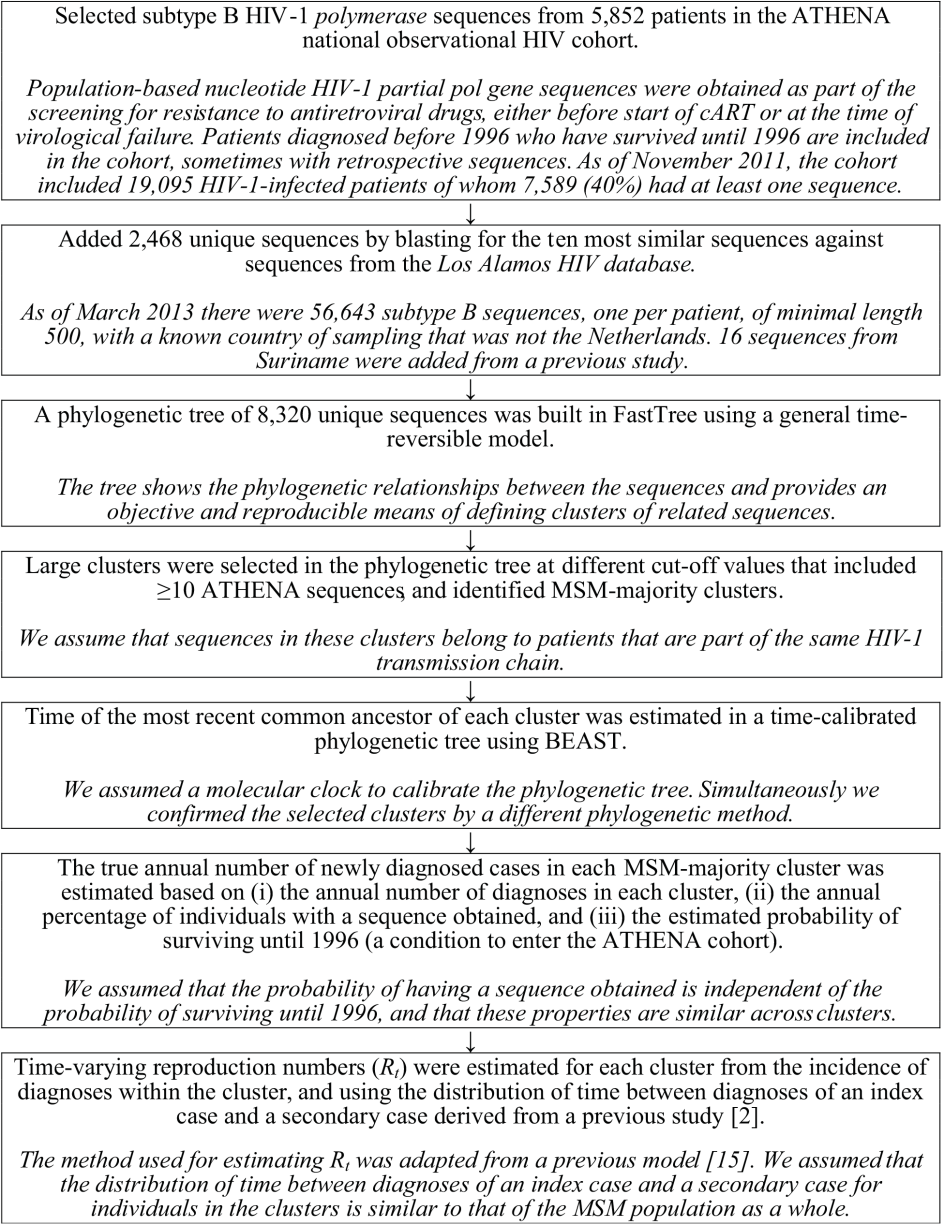
Flow diagram describing the methods and underlying assumptions.

The methods used in this study, along with the underlying assumptions, are summarized in Fig 1 and explained in detail below and in S1 Text.

### Patient Cohort

The ATHENA national observational HIV cohort includes anonymized data from all HIV-infected patients followed longitudinally in the 27 HIV treatment centers, in the Netherlands since 1 January 1996 and in the St. Elisabeth Hospital in Willemstad, Curaçao, since 1 January 2005, except 1.5% who opt out [16]. Patients who were diagnosed between 1981 and 1995 were included in the cohort if they were still alive in 1 January 1996 [2]. Demographic data were collected at entry in the cohort, including self-reported most likely risk group of infection, and most likely country of infection. Population-based nucleotide HIV-1 partial *pol* gene sequences were obtained as part of the screening for resistance to antiretroviral drugs, either before start of cART or at the time of virological failure, sometimes retrospectively, as described in detail previously [11,17]. Subtype B sequences were identified by phylogenetic analysis using reference sequences from the Los Alamos HIV Sequence Database [18]. As of 1 November 2011, the ATHENA cohort included 19,095 HIV-1-infected patients, of whom 7,589 (40%) had at least one sequence; 5,852 (77%) patients were infected with a subtype B strain.

At initiation, the ATHENA observational cohort was approved by the institutional review boards of all participating centers. It has subsequently become an integral part of HIV care and includes anonymized data and stored plasma samples from HIV-infected patients living in the Netherlands and receiving care in one of the designated HIV treatment centers. Patients can opt out after being informed by their treating physician of the purpose of collection of data and samples. Data from patients who opt out are not included in the ATHENA database. Anonymized data may be used for scientific purposes without further review. Patients are informed that if there are future requests for use of stored plasma samples for scientific research, they will be asked for prior consent by their treating HIV physician. Data are anonymized before being provided to investigators. For the purpose of our analysis, only existing data have been used, and therefore no additional review or consent has been necessary.

### Recent HIV-1 Infections

Recent HIV-1 infections were defined as infections with either (i) a seroconversion interval of 18 mo or less between the last negative and the first positive HIV-1 serology test or (ii) a diagnosis of primary HIV-1 infection defined as detectable HIV-1 RNA in plasma, detectable serum p24 antigen in plasma, or both, combined with either a negative HIV-1 antibody test or a positive HIV-1 antibody test with a negative or indeterminate HIV-1 Western blot.

### Patient Sequences in a Phylogenetic Context

A phylogenetic context for the HIV sequences in our patient cohort was provided by sequences that were selected from the Los Alamos HIV Sequence Database [18]. As of 15 March 2013, there were 56,643 subtype B sequences, one per patient, of minimal length 500, with a known country of sampling that was not the Netherlands. Sixteen sequences from Suriname were added from a previous study [19]. For each of the 5,852 subtype B sequences in our dataset, the ten most similar sequences were identified by BLAST 2.2.22+ [20]. In total, 2,468 unique sequences were added to the study.

### Sequence Alignment

A total of 8,320 unique sequences were aligned using Clustal Omega 1.1.0 [21] and manually checked and adjusted. Gblocks 0.91b was used to define the optimal sequence length of consistently aligned positions at 1,254 nucleotides [22]. Major resistance-conferring sites at the amino acid positions described by the International AIDS Society–USA were excluded for phylogenetic analysis, including alternative substitutions at position 215 and two insertions [23], resulting in an alignment length of 1,137 nucleotides.

### Transmission Clusters

A phylogenetic tree was built in FastTree 2.1.3 using a general time-reversible(GTR) model [24]. PhyloPart 2.0 was used to define transmission clusters as those clusters of sequences with a local support value ≥ 0.9 based on 1,000 resamples and for which the median value of all pairwise patristic distances of the sub-tree was below the fifth percentile threshold of the distribution of all pairwise distances of the whole tree [25], which corresponded to 0.068 mutations per site. We considered only large national transmission clusters with sequences from at least ten patients from within the ATHENA cohort [12]. The median of the median values of all pairwise distances per large cluster was 0.042 (interquartile range [IQR] 0.030–0.057) mutations per site. The duration of a large cluster was defined as the time between the earliest and latest date of diagnosis within the cluster. As a sensitivity analysis, comparisons for three different cluster definitions were made: a “looser” cluster definition, with a local support value ≥ 0.8 and with a median value of all pairwise distances under the tenth percentile threshold, a “more stringent” cluster definition, with a local support value ≥ 0.95 and with a median value of all pairwise distances under the 2.5th percentile threshold, and a third cluster definition, with a local support value ≥ 0.9 and a single linkage distance threshold of 0.042 mutations/site.

### Bayesian Time-Scaled Phylogenetic Analysis

We applied a Bayesian Markov chain Monte Carlo (MCMC) method as implemented in BEAST 1.7.5 for phylogenetic analysis of time-stamped sequence data to calculate the time of the most recent common ancestor (MRCA) of each large cluster. This value would indicate the time point since when each cluster has been circulating in the Netherlands, and simultaneously we confirmed the selected clusters by a different phylogenetic method. This was done by grouping clusters together in batches of approximately 200 sequences. Sequences from the Los Alamos HIV Sequence Database were included when the national cluster included ≥5 time-stamped sequences obtained in another country [26]. We used the uncorrelated log-normal relaxed molecular clock with a discretized gamma-distributed general time-reversible substitution model and the Bayesian skyride coalescent model [27,28]. Analyses of 200 million MCMC generations were performed, and evolutionary parameters were sampled every 20,000 generations to retrieve a distribution of posterior trees. After removing 10% of the initial output for burn-in, convergence of the MCMC output with effective sample size above 200 was inspected and confirmed in Tracer [29]. Maximum clade credibility (MCC) trees keeping the heights of the target tree were obtained using TreeAnnotator 1.8.0 after discarding 10% of the posterior trees as burn-in and were visualized using FigTree 1.4.2 [29]. Node ages and correspondent 95% highest posterior density (HPD) intervals were taken as the time of the MRCA and corresponding uncertainty estimates.

### Estimation of the Time-Varying Case Reproduction Number within Transmission Clusters

We analyzed HIV-1 transmission dynamics within each of the large transmission clusters containing a majority of MSM. For each cluster, we estimated the case reproduction number for each year *t*, *R*_*t*_, defined as the average number of secondary cases infected by an index case diagnosed in year *t*. We used an extension of the method introduced by Wallinga and Teunis [15], which allows estimation of *R*_*t*_ based on the incidence of diagnosis time series and what we call the diagnosis interval distribution, i.e., the distribution of the time from diagnosis of an index case to diagnosis of a secondary case.

We extended the Wallinga and Teunis method to allow the diagnosis interval to vary according to the time of diagnosis of the index case, and to allow for the possibility that the diagnosis interval can be negative, because it is possible that individuals may have been infected by index cases who had not yet been diagnosed. The likelihood that case *i* has been infected by case *j* is decomposed into the product of the likelihood that the index case of *i* has been diagnosed and the relative likelihood that if the index case of *i* has been diagnosed, it is a particular individual *j*. The relative likelihood that case *i* has been infected by case *j* (rather than by another case diagnosed up to year *T*, here *T* = 2010) is given by

$$p_{ij}^{*}=\frac{w_{t_{j}}(t_{i}-t_{j})}{\sum_{k,k\neq i}w_{t_{k}}(t_{i}-t_{k})}$$ (1)

where *w*_*t*_(.) denotes the distribution of the diagnosis interval for index cases diagnosed in year *t*.

The likelihood that case *i* has been infected by a case observed up to year *T* can be approximated by

$$q_{i}=\frac{\sum_{t=0}^{T}w_{t}(t_{i}-t)}{\sum_{t=0}^{+\infty }w_{t}(t_{i}-t)}$$ (2)

assuming that the incidence of diagnosed cases is constant over time (see S1 Text). Additionally assuming that the diagnosis interval distribution remains unchanged after *T*, this ratio simplifies to

$$q_{i}=\frac{\sum_{t=0}^{T}w_{t}(t_{i}-t)}{\sum_{t=0}^{T}w_{t}(t_{i}-t)+\sum_{t=T+1}^{T+S}w_{T}(t_{i}-t)}$$ (3)

where *S >* 0 is the maximum possible negative diagnosis interval, such that for all *u < −S* and *w*_*T*_(*u*) *=* 0, the total likelihood that a diagnosed case *i* has been infected by case *j* (rather than by another case, diagnosed or not) is given by

$$p_{ij}=p_{ij}^{*}\times q_{i}=\frac{w_{t_{j}}(t_{i}-t_{j})}{\sum_{k,k\neq i}w_{t_{k}}(t_{i}-t_{k})}\times \frac{\sum_{t=0}^{T}w_{t}(t_{i}-t)}{\sum_{t=0}^{T}w_{t}(t_{i}-t)+\sum_{t=T+1}^{T+S}w_{T}(t_{i}-t)}.$$ (4)

Note that the derivation of *q*_*i*_ assumes a constant incidence over time. Analyses presented in S1 Text show how *q*_*i*_ is modified when this assumption does not hold, and these analyses indicate that the resulting estimates of the reproduction number show consistent temporal trends regardless of the validity of this assumption, although the actual values may vary (S2 and S3 Figs).

The time-varying diagnosis interval *w*_*t*_(.) was obtained by numerically simulating a compartmental HIV transmission model previously fitted to HIV and AIDS diagnosis data on MSM in the Netherlands, where it is assumed no transmission occurs when individuals are on treatment (see S1 Text) [2]. This mathematical model was used to estimate the time-varying diagnosis interval for the whole HIV epidemic amongst MSM, and not to directly fit to cluster dynamics (see S1 Fig).

We further extended the Wallinga and Teunis method to incorporate uncertainty in the number of individuals diagnosed in a certain year *t*, *D*_*t*_. The ATHENA cohort records the number *S*_*t*_ of individuals diagnosed in year *t* who survived until 1996 and for whom a *pol* sequence was available. Therefore, we assumed that *D*_*t*_ = *X*_*t*_ + *S*_*t*_, with *X*_*t*_ ∼ Negbin(*S*_*t*_, 1−φ_*t*_×π_*t*_), where φ_*t*_ is the probability that an individual diagnosed in year *t* survives until 1996 (taken from [2]) and π_*t*_ is the probability that the virus is sequenced (taken from the ATHENA records) given survival up to 1996.

To estimate *R*_*t*_ for each cluster, we first sampled *n =* 50 realizations of *D*_*t*_ from this negative binomial distribution. For each of these, we then sampled 50 possible transmission chains (for each transmission chain, the index case of each secondary case *i* is sampled according to a multinomial distribution with probabilities *p*_*ij*_), leading to a total of 2,500 likely transmission chains. For each chain, we counted the number of secondary cases corresponding to each case, and averaged over index cases diagnosed in the same year to get one *R*_*t*_ trajectory over time. We pooled the 2,500 trajectories and used their mean and 2.5–97.5 quantiles as the point estimate and 95% confidence interval of *R*_*t*_. Before pooling, each *R*_*t*_ trajectory was corrected for right censoring to account for the fact that some of the secondary cases of index cases diagnosed in year *t* may not have been diagnosed yet, which may bias downwards the estimates of *R*_*t*_, with a stronger effect in recent years. To correct for this effect, *R*_*t*_ was divided by $\sum_{u=-S}^{2010-t}w_{t}(u)$, which represents the expected proportion of secondary cases of index cases diagnosed in year *t* who should be diagnosed in or before 2010, should overall transmission rates remain constant. Note that although this correction assumes that overall transmission rates will remain constant in the future, it still allows for heterogeneity in the times at which secondary cases appear relative to a given index case. We performed a sensitivity analysis to check that this right censoring correction was not introducing a systematic bias in the recent estimates of *R*_*t*_ (see S4 Fig).

## Results

### Study Population and Phylogenetic Tree

Characteristics of the ATHENA patients are shown in Table 1. Of the 5,852 patients with a HIV-1 subtype B *pol* sequence in this study, 219 (4%) were registered in the cohort in Curaçao and 5,644 were registered in the Netherlands, of whom 4,288 (73%) reported being MSM, 207 (4%) reported being persons who inject drugs (PWID), and 849 (15%) reported being infected via heterosexual contact. Of HIV-positive MSM registered in the Netherlands and diagnosed before 1 January 1996, 35% (781/2,218) have a *pol* sequence included, versus 43% (3,507/8,247) of those diagnosed since 1 January 1996. Overall, 48% of MSM registered in the cohort in the Netherlands are treated in the capital city, Amsterdam, and account for 51% of the sequences in this study; 25% of MSM are treated in the other big cities in the western part of the country and account for 27% of the sequences. S7 Fig shows the phylogenetic tree of a total of 8,320 unique *pol* sequences, including sequences obtained from the Los Alamos HIV Sequence Database.

**Table 1:** Patient baseline information.

Characteristic	All HIV-1-Infected Patients	Patients with HIV-1 Non-B Subtype	Patients with HIV-1 Subtype B				
			**All**	**MSM in the Netherlands**	**PWID in the Netherlands**	**HT in the Netherlands**	**Patients in Curaçao**
***n***	19,095	1,737	5,852	4,288	207	849	219
**Self-reported risk group of infection, *n* (percent)**							
MSM	10,465 (55%)	249 (14%)	4,340 (74%)	4,288	—	—	52 (24%)
HT	6,258 (33%)	1,228 (71%)	996 (17%)	—	—	849	147 (67%)
HT–male	2,760	488 (28%)	526 (9%)	—	—	447	79 (36%)
HT–female	3,498	740 (43%)	470 (8%)	—	—	402	68 (31%)
PWID	736	22	207 (4%)	—	207	—	0
Other and unknown	1,636	238	309 (5%)	—	—	—	20 (9%)
**Age at diagnosis (years), median (IQR)**	36 (IQR 29–43)	32 (IQR 26–40)	37 (IQR 30–44)	37 (IQR 30–43)	32 (IQR 27–39)	34 (IQR 28–44)	38 (IQR 29–46)
**CD4 cell count at diagnosis (cells/μl), median (IQR)**	328 (IQR 130–530) (*n =* 13,308)	284 (IQR 110–480) (*n* = 1,350)	370 (IQR 186–560) (*n =* 4,335)	390 (IQR 210–570) (*n =* 3,302)	430 (IQR 190–670) (*n =* 83)	291 (IQR 80–511) (*n =* 656)	307 (IQR 101–449) (*n =* 105)
**Year of diagnosis, median (IQR)**	2002 (IQR 1997–2007)	2004 (IQR 2001–2007)	2004 (IQR 1997–2007)	2005 (IQR 1998–2008)	1994 (IQR 1990–1997)	2004 (IQR 1999–2007)	2002 (IQR 1998–2005)
**Year of diagnosis, range**	1980–2011	1981–2010	1981–2010	1981–2010	1982–2010	1985–2010	1984–2010
**Year sample sequenced, median (IQR)**	—	2006 (IQR 2003–2008)	2006 (IQR 2003–2008)	2006 (IQR 2003–2008)	2002 (IQR 1997–2005)	2006 (IQR 2003–2008)	2005 (IQR 2004–2009)
**Year sample sequenced, range**	—	1991–2011	1986–2011	1986–2011	1987–2010	1996–2011	1999–2011
**Percent resistant mutations to any drug (PI, NNRTI, NRTI)**							
All patients	—	—	30% (10%, 12%, 24%)	28% (9%, 11%, 23%)	41% (13%, 15%, 37%)	32% (10%, 16%, 26%)	33% (15%, 10%, 29%)
Treatment-naive patients	—	—	12% (2%, 5%, 7%) (*n =* 4,051)	12% (2%, 4%, 7%) (*n =* 3,161)	8% (2%, 3%, 2%) (*n =* 86)	11% (2%, 6%, 5%) (*n =* 546)	4% (1%, 1%, 3%) (*n =* 91)
Patients with recent infection	—	—	12% (2%, 4%, 7%) (*n =* 1,265)	12% (2%, 4%, 7%) (*n =* 1,117)	10% (0%, 5%, 5%) (*n =* 40)	13% (3%, 4%, 6%) (*n =* 71)	0% (*n =* 6)
**Region of origin, *n* (percent)**							
Netherlands	10,377 (54%)	442 (25%)	3,914 (67%)	3,168 (74%)	119 (57%)	436 (51%)	6 (3%)
Former Dutch Antilles	1,236 (6%)	16 (1%)	366 (6%)	105	2 (1%)	79 (9%)	166 (76%)
Suriname	805 (4%)	22 (1%)	313 (5%)	125 (3%)	4 (2%)	159 (19%)	3 (1%)
Latin America	652 (3%)	17 (1%)	274 (5%)	179 (4%)	-	43 (5%)	42 (19%)
Western Europe	1,228 (6%)	36 (2%)	400 (7%)	313 (7%)	32 (15%)	37 (4%)	—
Central Europe	335 (2%)	15 (1%)	118 (2%)	73 (2%)	7 (3%)	27 (3%)	—
Eastern Europe	114 (1%)	21 (1%)	31	23	1	5	—
North America	264 (1%)	3	94	79	4 (2%)	2	—
North Africa and Middle East	235 (1%)	13 (1%)	74	31	7 (3%)	3	—
Oceania	66	7	19	18	—	—	—
Sub-Saharan Africa	3,040 (16%)	1,063 (61%)	55	36	1	16 (2%)	—
South and Southeast Asia	590 (3%)	76 (4%)	120	95	4	17 (2%)	—
Australia	37	2	10	10	—	—	—
Unknown	139	3	82 (1%)	51 (1%)	26 (12%)	3	2
**Self-reported country of infection, *n* (percent)**							
Netherlands	10,223 (70%)	398 (33%)	3,918 (86%)	3,118 (91%)	141 (88%)	507 (79%)	8 (6%)
Former Dutch Antilles	537 (4%)	4	170 (4%)	17	1	36 (6%)	110 (83%)
US	179 (1%)	1	60 (1%)	51 (1%)	1	2	—
Suriname	146 (1%)	1	40 (1%)	7	—	27 (4%)	—
Other	3,547 (24%)	804 (67%)	343 (8%)	223 (7%)	17 (11	66 (10%)	14 (11%)
Unknown	4,463	529	1,321	872	47	211	87
**Region of hospital, *n* (percent)**							
Curaçao	700 (4%)	5	219 (4%)	—	—	—	219
Amsterdam	7,424 (39%)	712 (41%)	2,879 (51%)	2,201 (51%)	124 (60%)	414 (49%)	—
**In a cluster with ≥10 ATHENA sequences**	—	—	3,716 (52%)	2,196 (51%)	153 (74%)	474 (56%)	103 (47%)

HT, heterosexual transmission; NNRTI, non-nucleoside reverse transcriptase inhibitor; NRTI, nucleoside reverse transcriptase inhibitor; PI, protein inhibitor.

### Transmission Clusters

**Fig 2:**
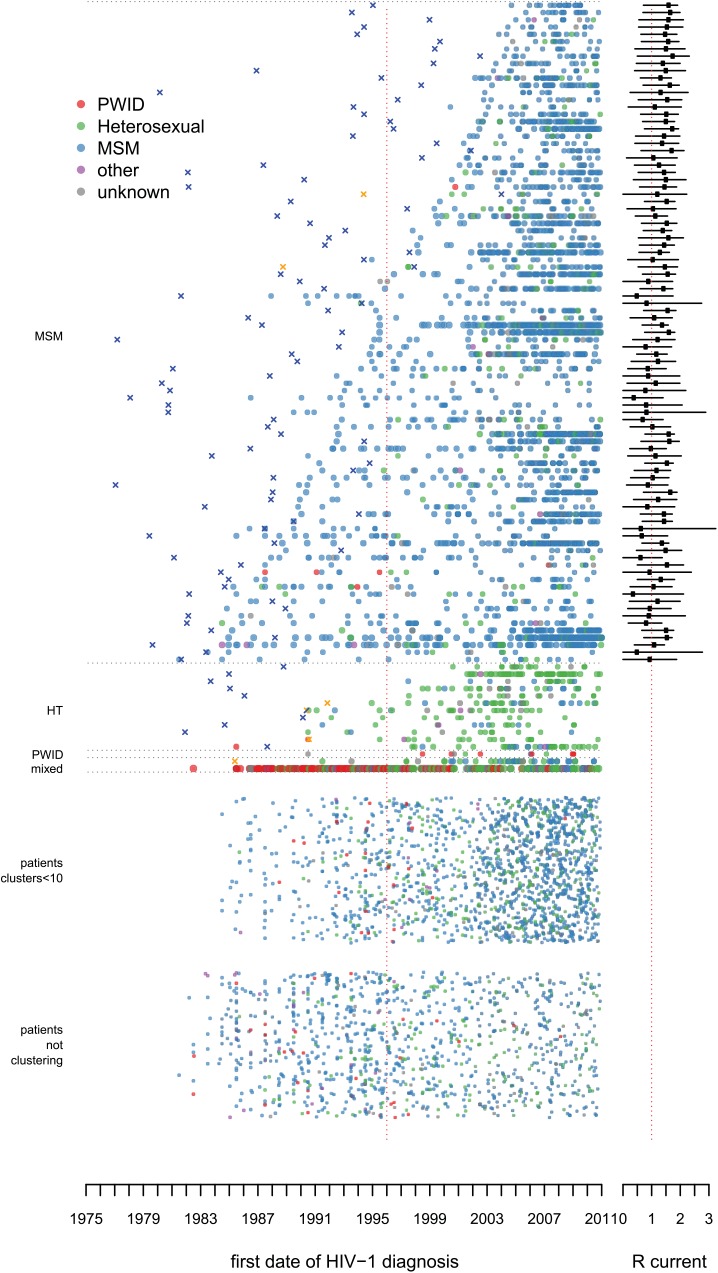
Large transmission clusters over time: risk group of infection. The picture illustrates the distribution of 106 large transmission clusters, where every horizontal line of dots represents one cluster, and each dot represents a single patient in the cluster by the year of diagnosis. The dots in a cluster represent in total 52% (3,061) of 5,852 ATHENA patients with a HIV-1 subtype B *pol* sequence in this study. The clusters are ordered by majority risk group and by the number of years between the first and last patient identified within each particular cluster. The color of each dot represents the self-reported risk group of infection. X’s indicate the estimated time of the MRCA, in orange for Curaçao. Some discrepancies may arise as the earliest cases sometimes are included with a sequence many years after their year of diagnosis. On the right-hand side the estimated mean reproduction number over the last 5 y is indicated. At the bottom of the figure, patients are represented who could not be identified as belonging to a cluster. The group above this one shows those patients who belonged to clusters in the phylogenetic tree with fewer than 10 ATHENA sequences included, which were not regarded as large clusters according to our definition. S8 Fig shows the same figure with also these smaller clusters stratified by duration. HT, heterosexual transmission.

**Fig 3:**
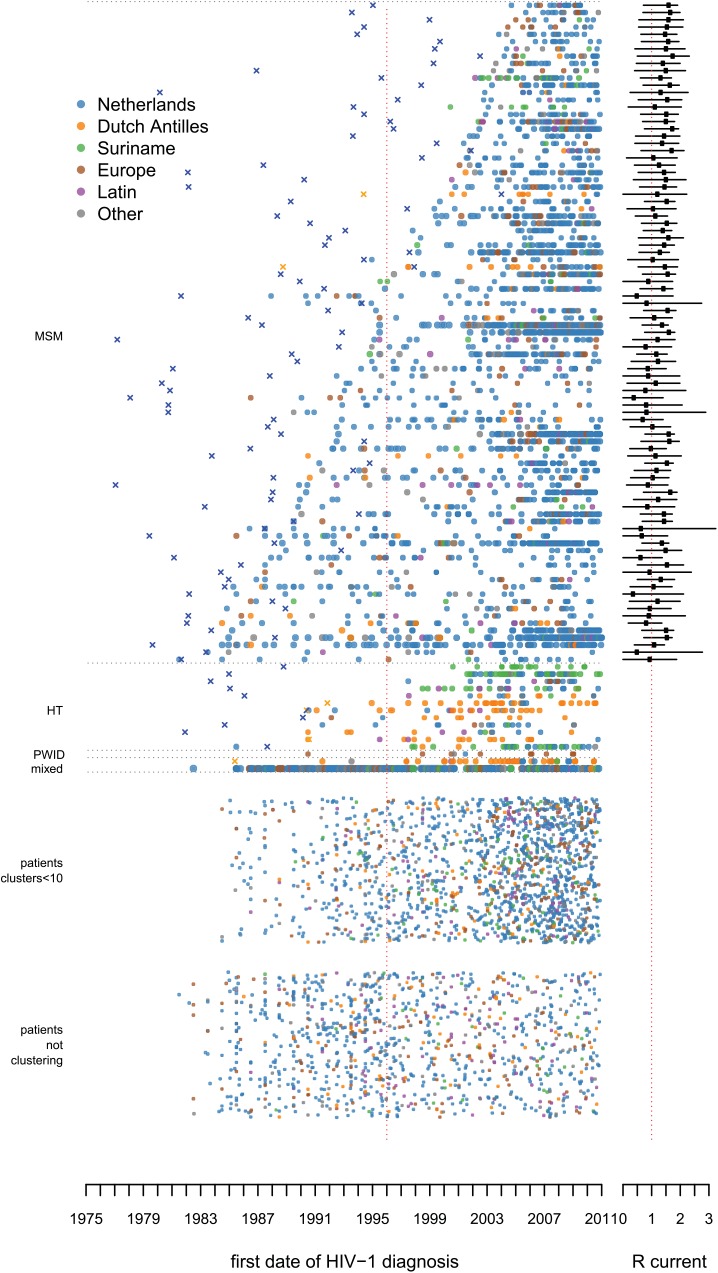
Large transmission clusters over time: region of origin. As in Fig 2, but here the color of each dot represents the region of origin. In large MSM-majority clusters 79% (1,673) of MSM were of Dutch origin. HT, heterosexual transmission.

From the total phylogenetic tree, 106 large transmission clusters were identified that included HIV *pol* sequences from ≥10 patients from the ATHENA cohort, illustrated in Fig 2 stratified by majority risk group of infection and cluster duration. The 106 clusters included 52% (3,061) of sequences from 5,852 ATHENA patients and 652 sequences obtained from the Los Alamos HIV Sequence Database. Only 15 of the 106 clusters were not MSM-majority clusters (≥50% MSM). Of these, 11 clusters were dominated by heterosexually infected individuals of Suriname or Antillean origin, one was dominated by PWID of Polish origin, and two were mixed heterosexual and MSM clusters of mostly Dutch origin. The largest cluster included sequences from 136 (42%) PWID—hence, from 66% of all PWID with a sequence in this study—and also included sequences from 122 (37%) heterosexually infected individuals and from 24 (7%) MSM. It also included 200 HIV *pol* sequences from the Los Alamos sequences. Fig 3 illustrates all clusters by region of origin. See S1 Trees for timed trees of all clusters and S1 Text for more detailed information on the largest PWID cluster and the transmission clusters circulating on Curaçao.

### MSM-Majority Clusters

**Fig 4:**
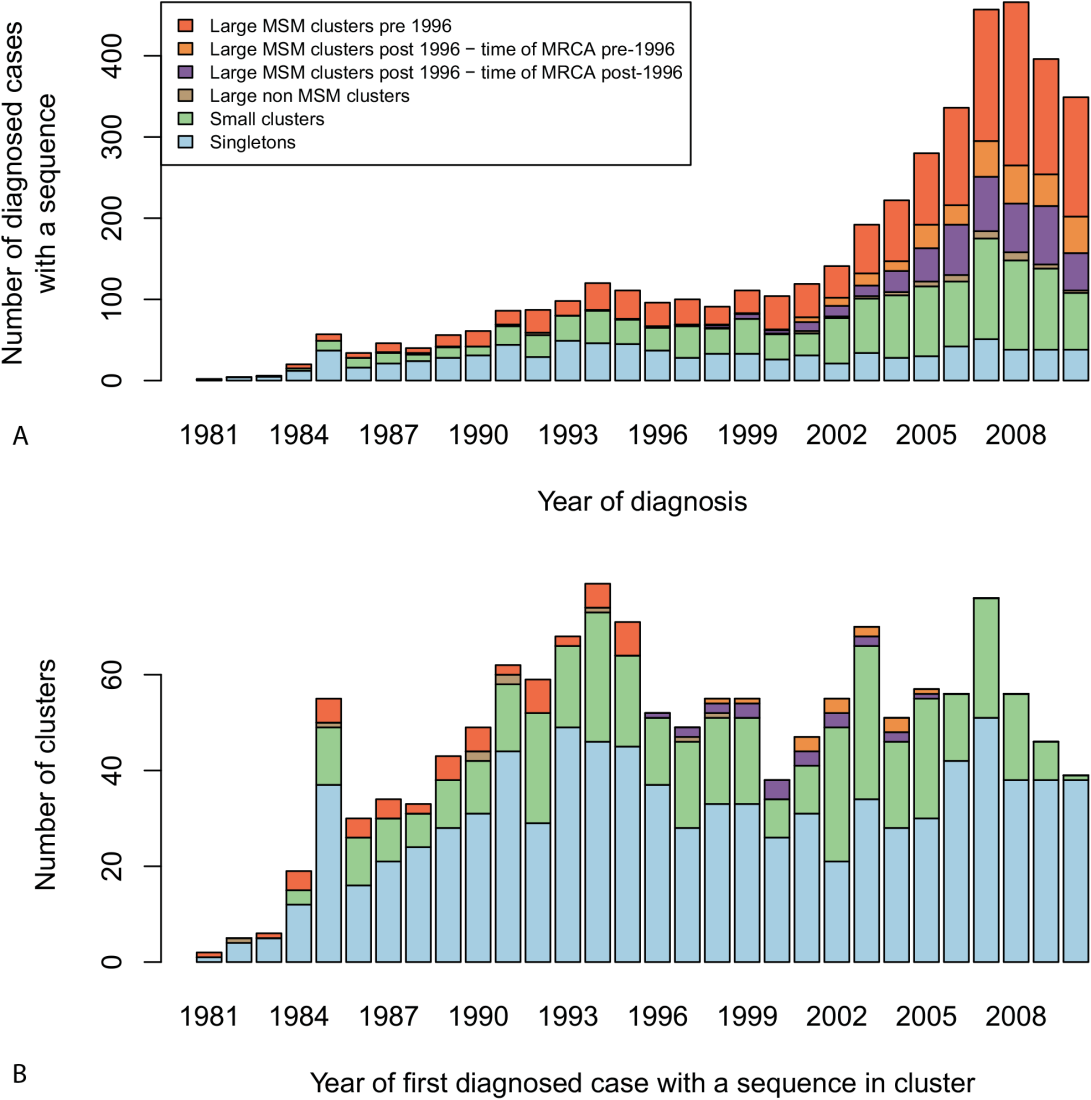
Diagnosis and growth of transmission clusters over time. Cluster types within the phylogenetic tree are defined as follows. Singletons (in blue) are clusters of size 1, or cases whose sequence solely clustered with sequences from the Los Alamos HIV Sequence Database. Small clusters (in green) comprise sequences from 2–9 ATHENA patients. Large clusters comprise sequences from ten or more patients in the ATHENA cohort. Amongst those, non-MSM-dominant clusters (in brown) contain a majority of sequences from non-MSM patients, whilst MSM-majority clusters contain a majority of sequences from MSM patients. Among large MSM-majority clusters, pre-1996 clusters (in dark orange) are defined as those in which the first diagnosed patient in the cluster was diagnosed before 1996, and post-1996 clusters are defined as those in which all patients in the cluster were diagnosed in or after 1996. Large MSM-majority post-1996 clusters are stratified as “time of MRCA pre-1996” (in light orange) when the estimated time of the MRCA is before 1996, and “time of MRCA post-1996” (in purple) when the estimated time of the MRCA is in or after 1996. (A) Number of MSM registered in the ATHENA cohort in the Netherlands with a sequence in this study by year of diagnosis and by cluster type. (B) Number of clusters of each type by year of first diagnosed case in each cluster.

Fifty percent (2,128) of the 4,288 MSM registered in the ATHENA cohort in the Netherlands with a sequence in this study were in 91 large MSM-majority (≥50%) transmission clusters including at least ≥10 patients from the ATHENA cohort. A further 28% (1,192) were in 417 small clusters containing 2–9 ATHENA sequences; 21% (900) were singletons, of whom 132 clustered solely with foreign sequences; and only 68 were in nine of the non-MSM-dominated clusters (Fig 4). The median number of MSM registered in the cohort in the Netherlands per large cluster was 16 (IQR 11–27, range 5–121).

**Fig 5:**
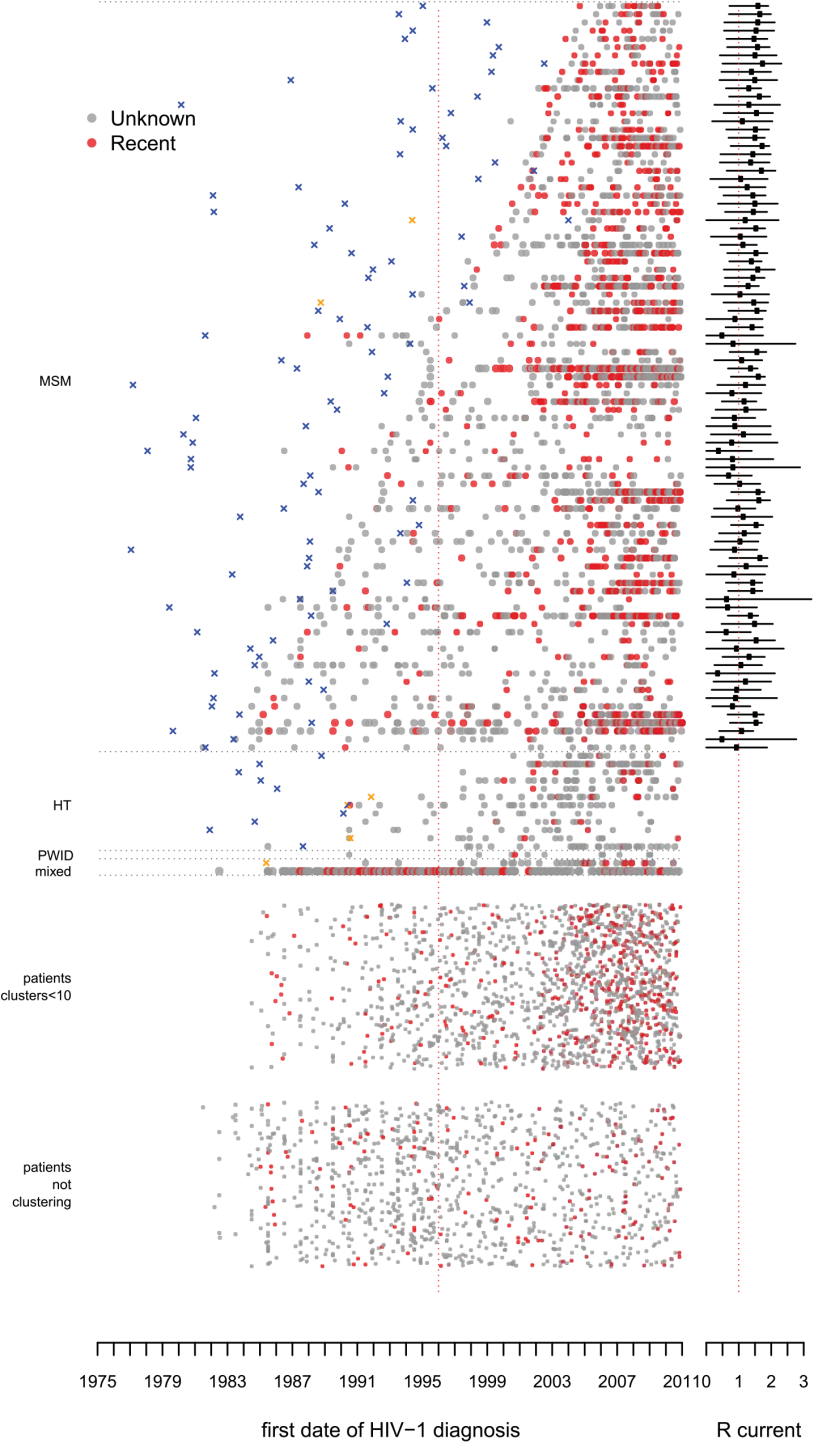
Large transmission clusters over time: recent infections. As in Fig 2, but here red dots represent patients with a documented recent infection. HT, heterosexual transmission.

At least 59% (54) of the 91 large MSM-majority clusters were already circulating before 1996 (Fig 4). Most clusters were confirmed in the BEAST analysis. Figs 2, 3, and 5 show the estimated time of the MRCA of all clusters, and the 95% HPD intervals are in S2 Table. These estimations indicate that 23 (95% HPD interval 8–33) of the clusters identified in or after 1996 (57%) might have started off before 1996, but also that 14 (95% HPD interval 4–29) of these clusters started off more recently (Fig 4).

Of 3,460 MSM with a sequence in this study who were registered in the ATHENA cohort in the Netherlands and diagnosed after 1996, 1,226 (35%) were infected within 54 transmission clusters circulating before 1996, and 20% (700) within 37 large clusters identified in or after 1996; of the latter, 429 (95% HPD interval 272–621) were in clusters with a time of the MRCA before 1996. Thus, in total, 48% (95% HPD interval 43%–53%) of these men diagnosed in or after 1996 were infected within a cluster that started circulating in the Netherlands before 1996. The proportion of these men diagnosed in the 54 clusters identified before 1996 increased over time to 42% in 2010; the proportion in either these clusters or clusters identified since 1996 but with a time of the MRCA before 1996 increased to 55% (95% HPD interval 53%–62%). Those diagnosed within any of the large clusters increased to 68% (Fig 4).

#### Recent infections

Of the 54 pre-1996 clusters, 78% (40) included recent infections after 2000 (Fig 5). Since 1996, a recent infection at diagnosis could be identified amongst 41% of MSM in large clusters, 31% of MSM in smaller clusters, and 22% of MSM singletons. Only three (6%) of the 54 pre-1996 clusters had no new diagnoses observed in the last 5 y of the study period, 2006–2010.

#### Country of infection

Amongst 3,416 MSM registered in the ATHENA cohort in the Netherlands with a sequence in this study who reported a most likely country of infection, 4.3% of MSM in large clusters reported likely having been infected abroad, and likewise 8.1% (risk ratio [RR] = 1.9; 95% CI 1.4–2.6) in smaller clusters, 19.0% (RR = 4.5; 95% CI 3.4–5.9) of singletons, and 27.8% (RR = 6.5; 95% CI 4.4–9.7) of MSM clustering solely with one or more Los Alamos sequences. This indicates that singletons, besides being part of unidentified clusters of national transmission, often represent new introductions.

#### Data versus theory

Fig 4 illustrates that only a small minority (7%) of all the observed phylogenetic MSM clusters (sum of singletons and small and large clusters) were established “large” transmission clusters including ten or more cases. This is broadly consistent with results from branching theory, which indicates that this proportion should be between 6% and 35% for a reproduction number between 0.8 and 1.2 (see S1 Text). Theoretical results also show that this observed proportion is a good approximation for the proportion of introductions leading to sub-epidemics of ten cases or more, with little sensitivity to the proportion of cases whose virus was sequenced.

### Estimation of the Case Reproduction Number within Large MSM-Majority Transmission Clusters

**Fig 6:**
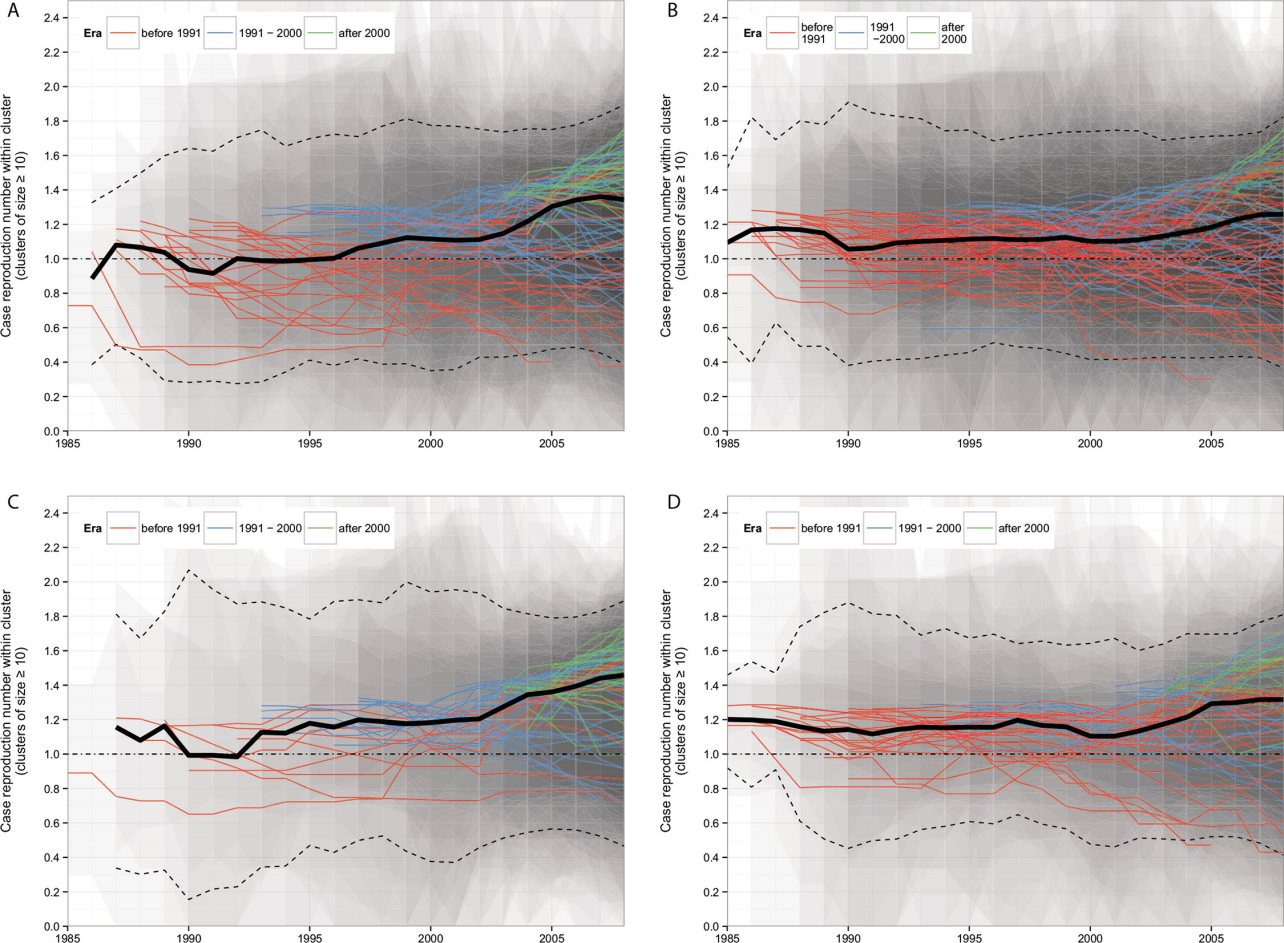
Estimated case reproduction number over time for all MSM-majority transmission clusters of ≥10 cases. The solid lines show the mean *R*_*t*_ estimate for each transmission cluster. The bold black line is the mean *R*_*t*_ of all clusters, with the 95% confidence interval shown by the dotted lines. The shaded areas show the 95% confidence intervals for each transmission cluster: darker areas indicate overlapping intervals across different transmission clusters. Transmission clusters are shown in red if their first sequence appeared before 1991, in blue if their first sequence appeared between 1991 and 2000, and in green if their first diagnosed case appeared after 2000. The black horizontal dotted line represents the threshold value *R*_*t*_ = 1. (A) Main analysis. (B) Sensitivity analysis for a looser cluster definition. (C) Sensitivity analysis for a more stringent cluster definition. (D) Sensitivity analysis for the clusters defined under a single linkage branch length threshold.

Fig 6 shows the estimated annual case reproduction number for all 91 large MSM-majority transmission clusters. With a reproduction number around one (the critical value that separates epidemic spread from contraction), clusters were self-sustaining over the whole study period. Reproduction numbers were strikingly consistent across clusters. A tendency towards higher numbers was visible over recent years, especially in the more recently introduced clusters (see S6 Fig). Seventy-five percent (68) of the clusters had a mean reproduction number greater than one over the last 5 y (Fig 2).

### Sensitivity Analysis

With the looser cluster definition, 105 large MSM-majority clusters were selected, containing 69% (2,977) of the HIV *pol* sequences from MSM registered in the ATHENA cohort in the Netherlands, and only 4% (183) of sequences were singletons. In this sensitivity analysis, 17% of all observed phylogentic MSM clusters (including the singletons) included ten or more cases. With the more stringent cluster definition, 67 clusters were identified, containing 39% (1,670) of the *pol* sequences from MSM registered in the cohort in the Netherlands. With clusters defined under the single linkage branch length threshold, 63 large MSM-majority clusters were selected, containing 69% (2,974) of the *pol* sequences from MSM registered in the cohort in the Netherlands. In this analysis also, only a small minority (5%) of all observed phylogentic MSM clusters included ten or more cases. The estimations of the case reproduction number for the large MSM-majority clusters in all three sensitivity analyses were similar to those in our main analysis (Fig 6).

### Age of MSM at Diagnosis

**Fig 7:**
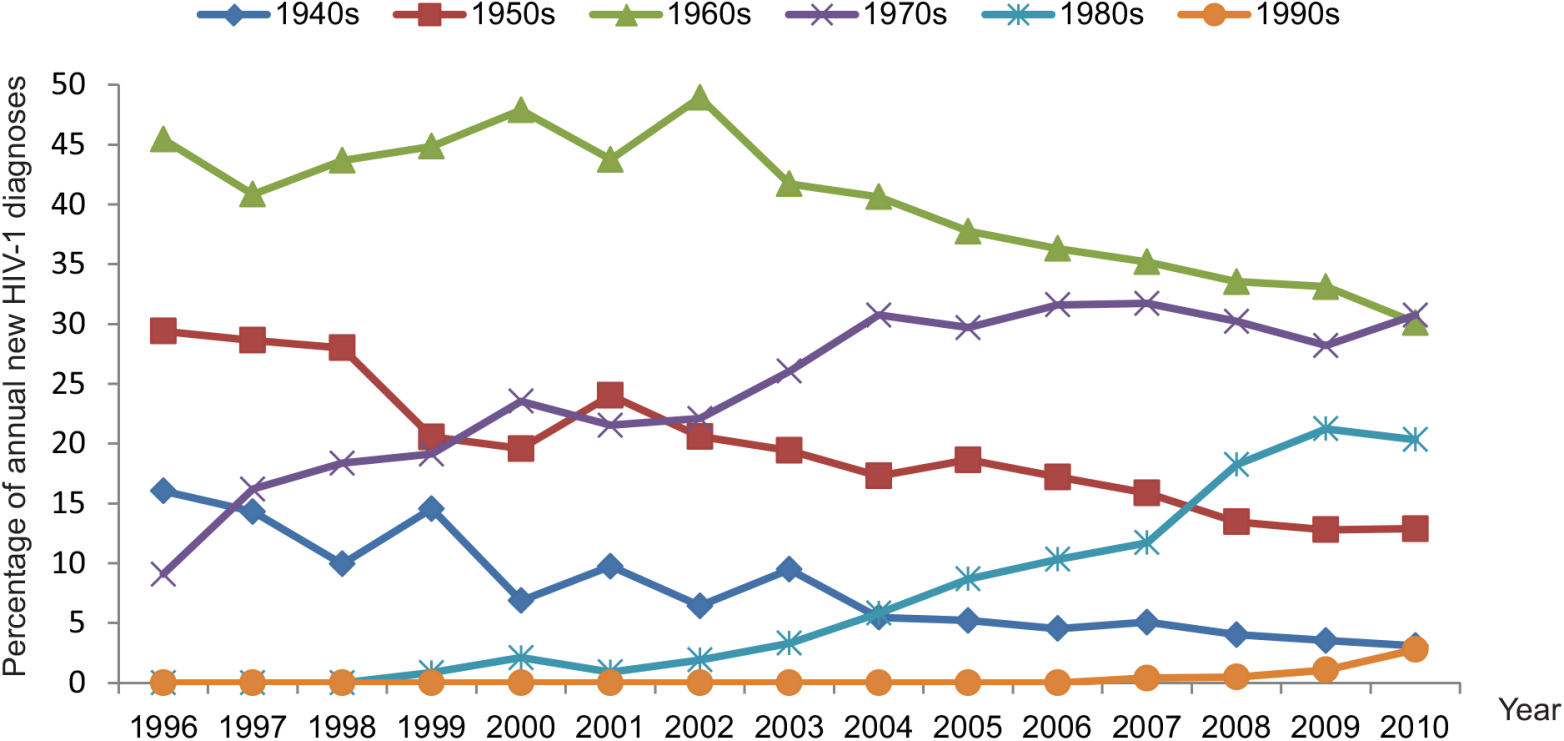
Proportional contribution of new HIV-1 diagnoses amongst all MSM in the ATHENA cohort by decade of birth.

Fig 7 shows that the HIV epidemic is shifting from the initially infected generations of MSM towards younger generations. The mean age at diagnosis of MSM in our study has been increasing by 0.31 years per calendar year overall. However, this overall trend hides more complicated dynamics because within each of the 91 large MSM-majority clusters, the mean age at diagnosis has increased more rapidly, at a rate of 0.45 years/year, compared to the epidemic as a whole. On the other hand, new clusters have started with a lower initial mean age, which, across clusters, has increased at rate of only 0.28 years/year.

## Discussion

This study provides insight into the different transmission clusters that have contributed to the ongoing resurgent HIV epidemic amongst MSM in the Netherlands. We found that clusters that started spreading before the introduction of cART in 1996 still constitute the main source for new HIV-1 subtype B infections (~55% in 2010). Calculations of the reproduction number separately for each cluster show that whilst the epidemic diminished at the population level during the 1990s [1,2,10], most of these clusters continued spreading in a self-sustaining manner. There is little indication that any of the clusters will stop in the near future as most clusters contain recent infections and are rejuvenated by the inclusion of younger men, and many have a reproduction number greater than the epidemic threshold. While all clusters appear to be aging, new clusters appear to be of lower mean age; if anything, these “younger” clusters have higher epidemic potential than the “older” clusters. It is also conceptually interesting to see the whole HIV-1 subtype B epidemic disaggregated in a single figure (Figs 2 and 3). This perspective illustrates the tight links between clusters on Curaçao with people from the same region living in the Netherlands [19,30–32]. It also shows that the largest transmission cluster, which is dominated by PWID, shows links to heterosexual transmission but is an almost entirely separate epidemic from that amongst MSM.

The median reproduction number of all large clusters together found in this study is consistent with our previous findings of the HIV epidemic amongst MSM in that it is around the epidemic threshold and increases in later years. However, our previous study reported the mean reproduction number aggregated over the whole epidemic, whilst our findings here are based on disaggregating the epidemic into its constituent sub-epidemics. We found that the resurgent epidemic is the result of existing transmission clusters, many of which have been spreading since the early epidemic, to which new transmission clusters are being added. There seems to be a high and continued rate of case importation, but with only a small minority of cases going on to establish new self-sustaining clusters (Fig 5). This heterogeneity of outcomes is in line with expectations from epidemiological theory, as we also show in S1 Text: self-sustaining sub-epidemics are hard to establish but, once initiated, are difficult to stop [33]. The higher percentage of recent infections in large clusters versus small clusters and singletons could indicate an effect of partner tracing. Several studies indicated that a large part of onward transmission amongst MSM is during the early phase of infection [11,34–37]. And thus it is worrying that even though an increasing number of MSM with HIV are diagnosed early in infection, this does not seem to have stopped clusters from growing.

The clusters and findings were confirmed using different phylogenetic methods and cluster definitions. However, by phylogenetic methods alone and using only *pol* sequences of only a subset of patients, we were not able to fully identify all transmission chains. The focus in our study was on the large transmission clusters. The observation that sequences from patients diagnosed before 1996 were less often part of a large cluster could indicate that some initial clusters have stopped circulating and have gone unnoticed. It is also possible that, since the sample fraction was lower in early years, fewer individuals appear to cluster (due to missing links), even though they were in fact linked to larger transmission clusters via unknown intermediates.

Our estimates of the reproduction number are based on a number of assumptions, including that the distribution of the diagnosis interval (time from diagnosis of an index case to diagnosis of a secondary case) is similar across clusters and similar across the whole HIV-positive population in the Netherlands. These estimates also assume that the probability of having a sequence obtained and the probability of surviving until 1996 are similar across clusters. It should be noted that the reproduction numbers estimated in this study are effective reproduction numbers. Our findings might thus indicate local or temporal saturation effects in certain sexual networks. However, as prevalence of HIV amongst MSM ranges from 5% to 8% [38], behavior change, not saturation, is expected to be the dominant driver of epidemic trends in this population. For a future study it would be interesting to compare findings, from real and simulated data, with recent studies that obtained reproduction numbers for large transmission clusters directly from sequence data [13,14].

This study, combining phylogenetic methods with mathematical modeling to analyze extensive HIV-1 subtype B sequence data, provides new insight into how different transmission networks together contribute to the resurgent HIV epidemic in the Netherlands. The analysis suggests that the epidemic amongst MSM is dispersed amongst a large number of self-sustaining or growing transmission clusters, many of which persisted throughout the 1990s, before increases in risk behavior became widespread. In particular, the relative homogeneity and consistency of reproduction number estimates for the different networks was unexpected. Our study highlights that many different sub-epidemics have independently persisted for decades, despite the widespread availability of treatment, steadily increasing rates of diagnosis, and increasing tendency for early treatment initiation. The fastest growing sub-epidemics are the newest ones, which also tend to be amongst the youngest men. Preventing further increases in rates of infection will require further developments in prevention services.

## Supporting Information

S1 Fig Distribution of the diagnosis interval stratified by year of diagnosis of the index case. (PDF)

S2 Fig *q*_*i*_ as a function of time in different scenarios regarding incidence growth over time. The black line shows the scenario with constant incidence over time, which was considered in the main text.
(PDF)

S3 Fig Influence of assumptions underlying *q*_*i*_ derivation on the estimates of yearly reproduction numbers *R* for the four largest transmission clusters. (PDF)

S4 Fig Estimated case reproduction number over time for all MSM-majority transmission clusters with ≥10 cases diagnosed before 2000. The solid lines show the mean *R*_*t*_ estimates for each transmission cluster using the dataset truncated after 2000 (black lines) and using the full dataset (blue lines). The shaded areas show the 95% confidence intervals for each transmission cluster (black shading for the dataset truncated after 2000, blue shading for the full dataset). The red horizontal line represents the threshold value *R*_*t*_ = 1.
(PDF)

S5 Fig Comparison of *R*_*t*_ in different time periods. (PDF)

S6 Fig Increasing *R*_*t*_. (A) Number of cases in the first 5 y of each cluster and overall sample fraction. (B) Rate of increase in the mean estimated *R*_*t*_ over the first 5 y for each cluster.
(PDF)

S7 Fig Phylogenetic tree of a total of 8,320 HIV-1 subtype B *pol* sequences. Branches corresponding to the 5,852 ATHENA patients are colored by self-reported risk group of infection. HT, heterosexual transmission.
(PDF)

S8 Fig Large transmission clusters over time. As in Fig 2A, but including the small clusters including 2–9 ATHENA sequences sorted by duration.
(PDF)

S9 Fig Timed tree of HIV-1 *pol* sequences from patients in the six largest clusters on Curaçao. (A) Branches are colored according to region of residence. (B) Branches are colored according to the self-reported risk group of infection. The root of each cluster is indicated in bold. HT, heterosexual transmission.
(PDF)

S10 Fig Observed and expected proportion of singletons and small clusters. Under the Poisson (left) and geometric (right) model. The top three rows show how these proportions change as a function of π, the proportion of cases with a sequence, for a mean number of offspring of *R* = 1, *R* = 0.9, and *R* = 1.1, respectively. The vertical red dotted lines indicate the observed proportion of cases with a sequence, π = 0.3 over the whole time period considered. The bottom row shows how the proportions of singletons and small clusters change as a function of *R*, the mean number of offspring, for a proportion of cases with a sequence of π = 0.3. In all plots, the black and grey curves show the expected and observed proportion of singletons, respectively, with dotted lines indicating confidence intervals around the observed proportion; the dark and light blue curves show the expected and observed proportion of small clusters (size 2–9), with dashed lines indicating confidence intervals around the observed proportion.
(PDF)

S1 Table Phylogenetic trait association test on structuring within the largest HIV-1 subtype B transmission cluster in the Netherlands. To test whether HIV-1 *pol* sequences in the last 1,000 posterior trees obtained by BEAST were structured more strongly by risk group for the ATHENA sequences and more strongly by country for the sequences from the Los Alamos HIV Sequence Database [18] than expected by chance alone, a phylogenetic trait association analysis using Bayesian tip-association significance testing was performed [39]. The maximum monophyletic clade (MC) size statistic was estimated for each trait, which provides an estimate of the mean cluster size of sequences sampled for each trait. Significant clustering is defined as critical (*p* < 0.01), marginally significant (0.01 ≤ *p* ≤ 0.05), or not significant (*p* > 0.05).
(PDF)

S2 Table Time of the MRCA and 95% HPD intervals of the 91 large majority MSM phylogenetic clusters. (XLS)

S1 Text Supporting information on methods, sensitivity analysis, and results. (PDF)

S1 Trees Timed trees of 91 MSM-majority clusters. The first number of the branch name is the cluster name. Bars indicate the 95% HPD intervals of the node ages. In total, 380 of the Los Alamos sequences selected are included in the 91 large MSM-majority clusters. Twenty-six clusters have no Los Alamos sequences included. Nine clusters include ≥5 time-stamped sequences from a particular country (Foreign). Although some mixing is visible, these sequences form mainly separate clusters within the respective countries. Timed tree of the 15 other clusters (Other): four clusters with a majority of patients of Suriname origin, three clusters with a majority of patients of Antillean origin, two clusters of heterosexually infected individuals and MSM of mainly Dutch origin, one cluster with mainly PWID of Polish origin, the largest PWID cluster, and the six clusters on Curaçao with bars (two are also in MSM).
(ZIP)

## Abbreviations

cART: combination antiretroviral therapy
HPD: highest posterior density
IQR: interquartile range
MCMC: Markov chain Monte Carlo
MRCA: most recent common ancestor
MSM: men who have sex with men
pol: polymerase
PWID: persons who inject drugs
RR: risk ratio
