# Supplementary figures and images for: Dispersion of the HIV-1 Epidemic in Men Who Have Sex with Men in the Netherlands: A Combined Mathematical Model and Phylogenetic Analysis
Source: PLoS Med. 2015 Nov 3;12(11):e1001898. doi: 10.1371/journal.pmed.1001898 (PMC4631366; doi:10.1371/journal.pmed.1001898)

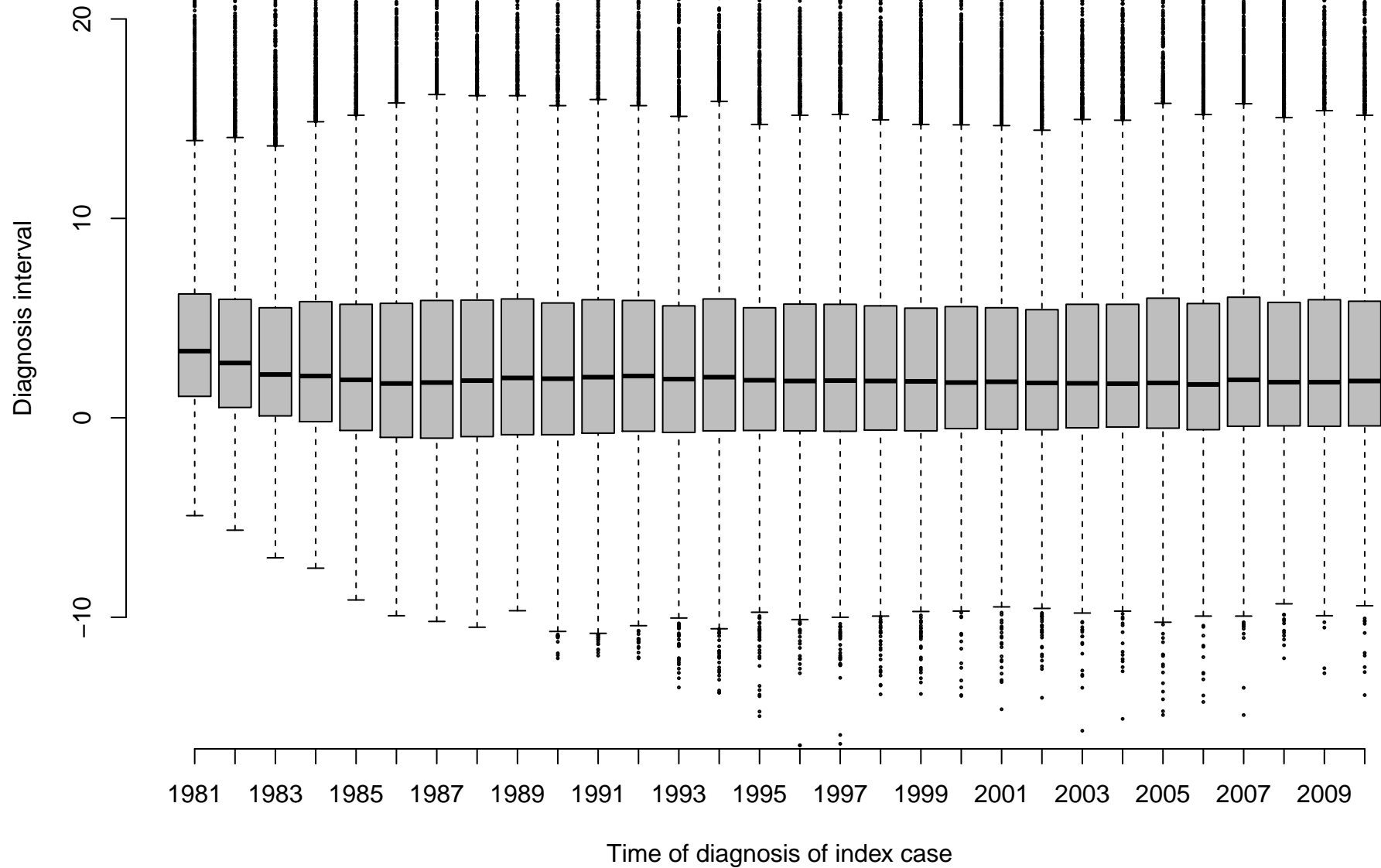

Supplement: S1 Fig — (PDF) [file pmed.1001898.s001.pdf]

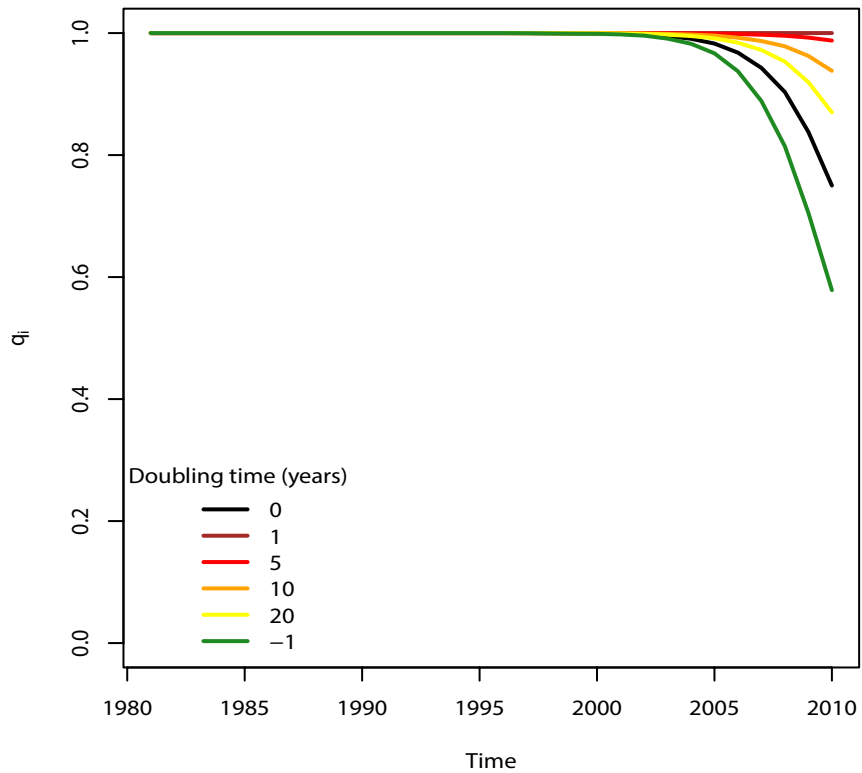

Supplement: S2 Fig — The black line shows the scenario with constant incidence over time, which was considered in the main text. (PDF) [file pmed.1001898.s002.pdf]

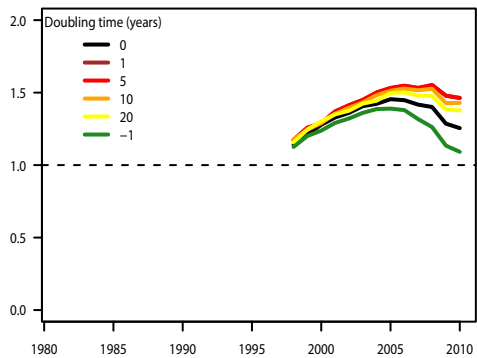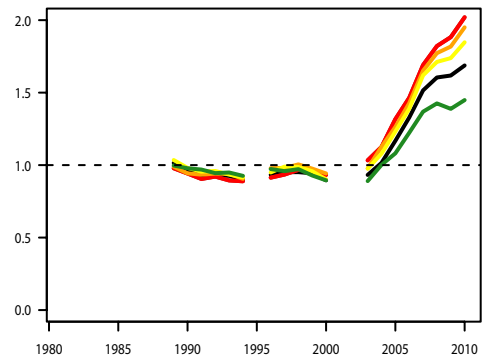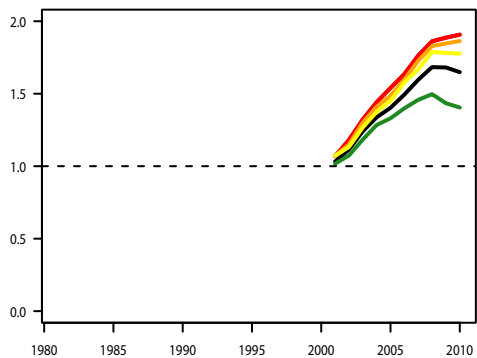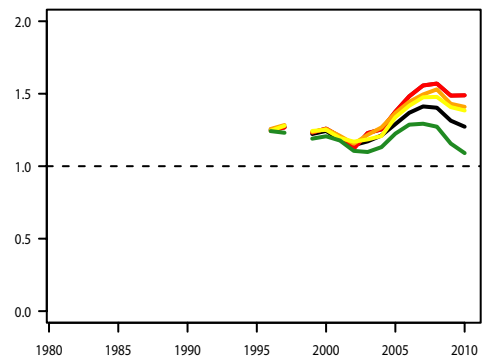

Supplement: S3 Fig — (PDF) [file pmed.1001898.s003.pdf]

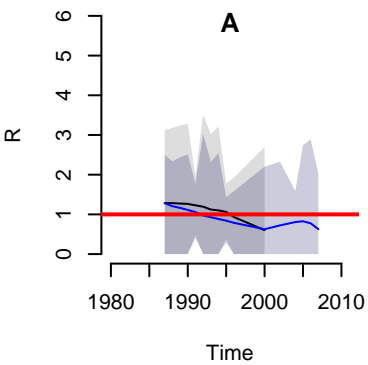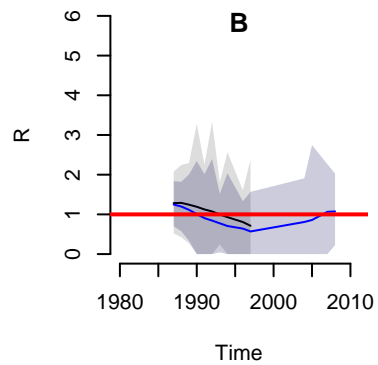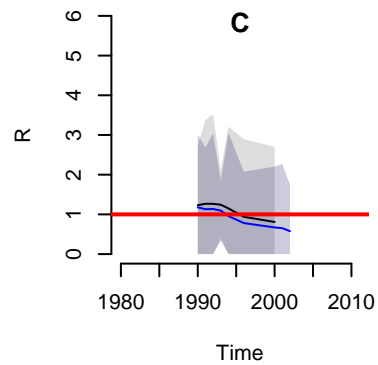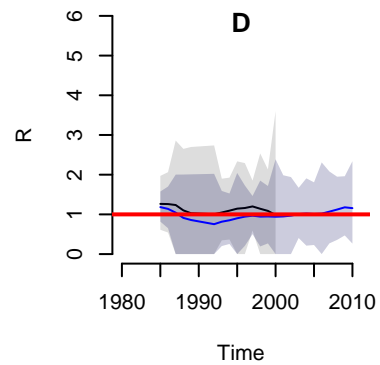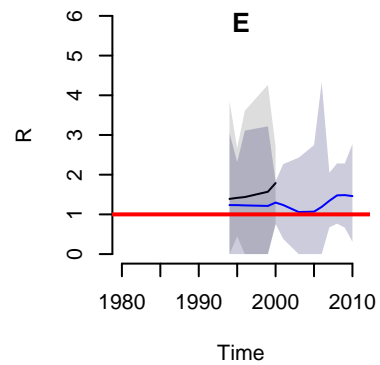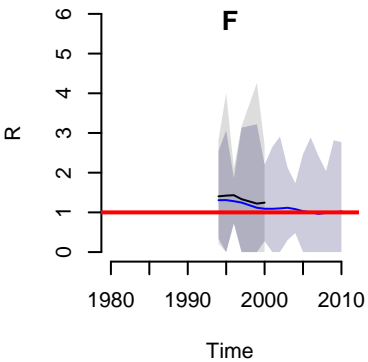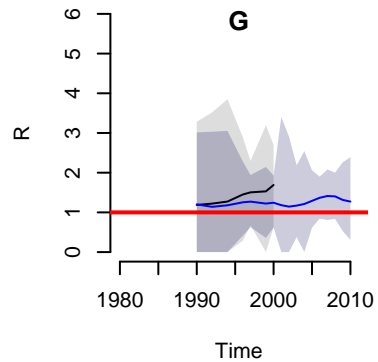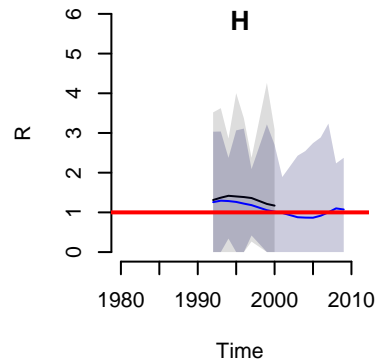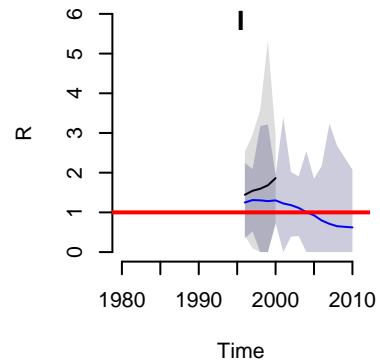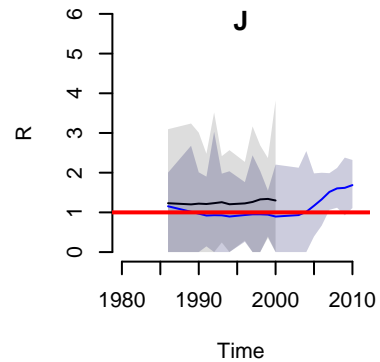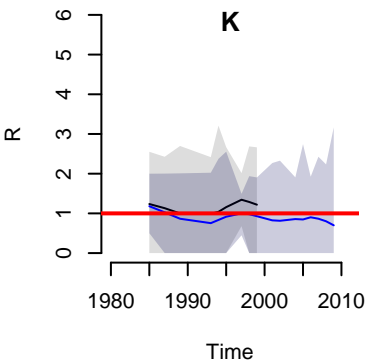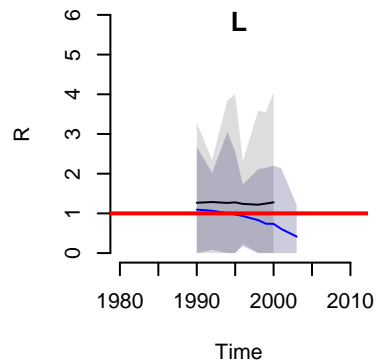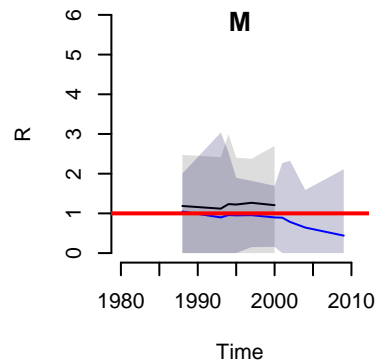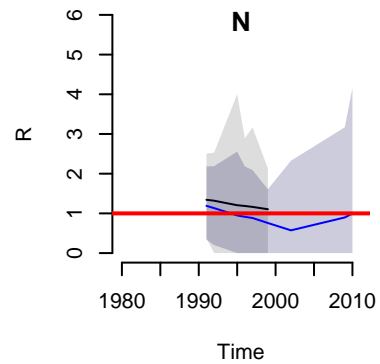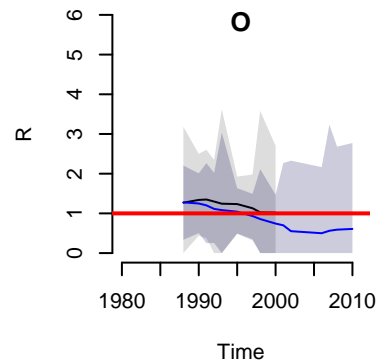

Supplement: S4 Fig — The solid lines show the mean R t estimates for each transmission cluster using the dataset truncated after 2000 (black lines) and using the full dataset (blue lines). The shaded areas show the 95% confidence intervals for each transmission cluster (black shading for the dataset truncated after 2000, blue shading for the full dataset). The red horizontal line represents the threshold value R t = 1. (PDF) [file pmed.1001898.s004.pdf]

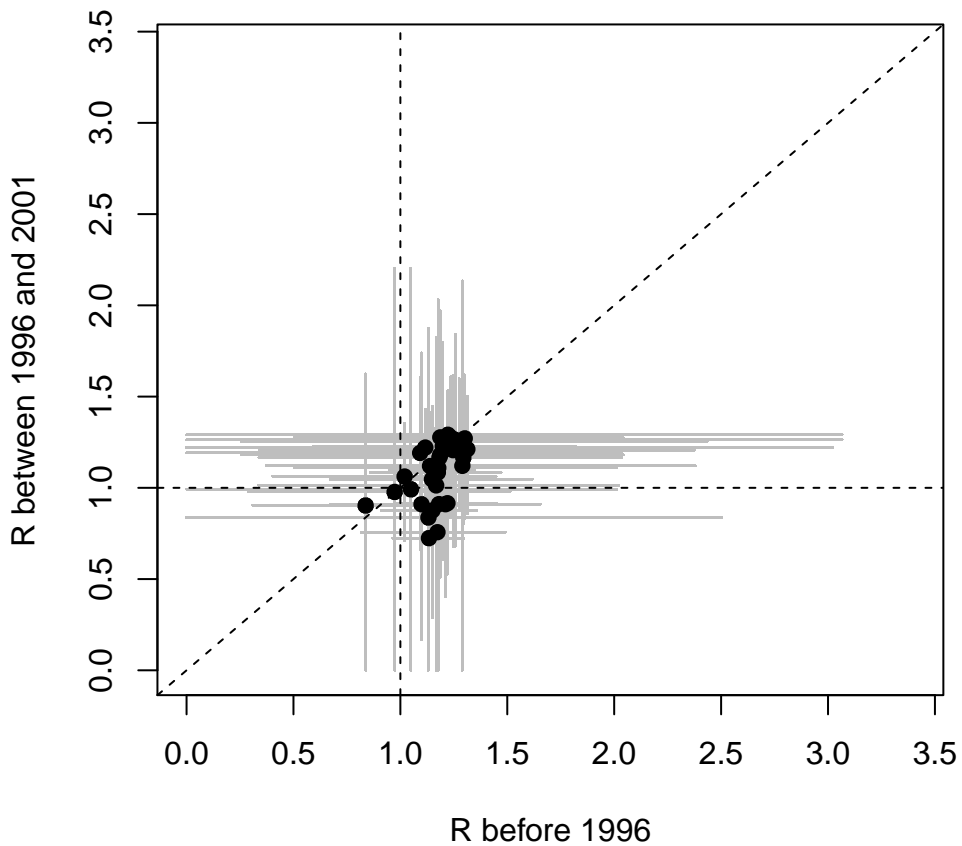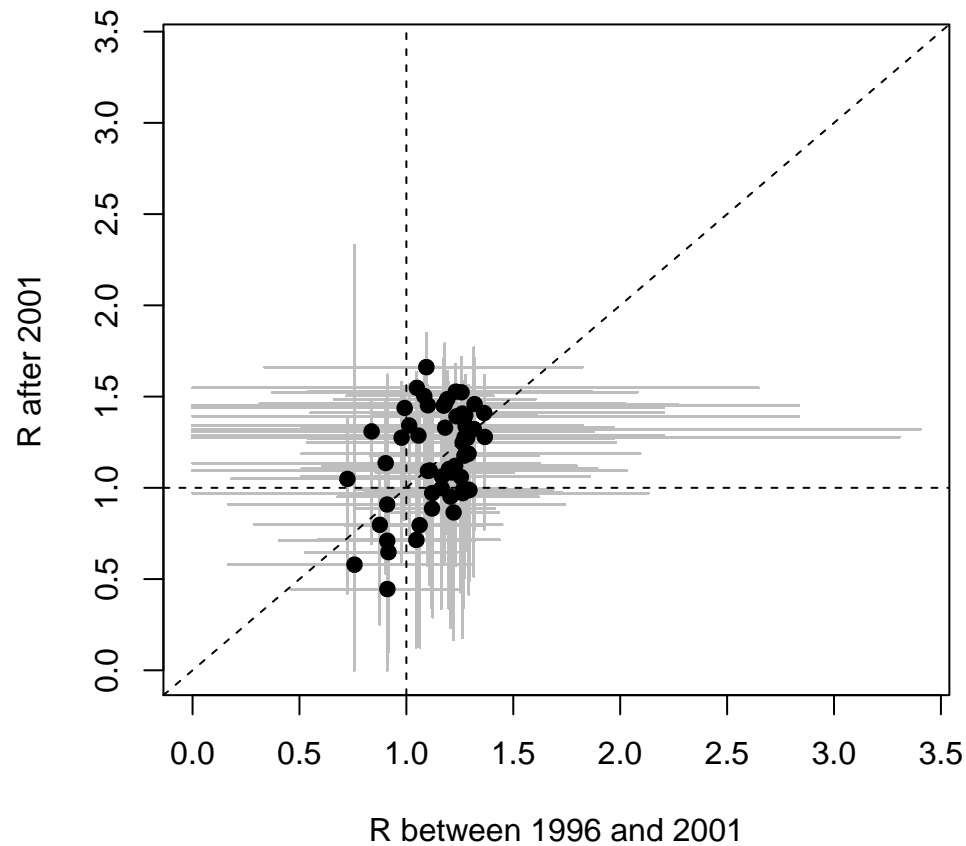

Supplement: S5 Fig — (PDF) [file pmed.1001898.s005.pdf]

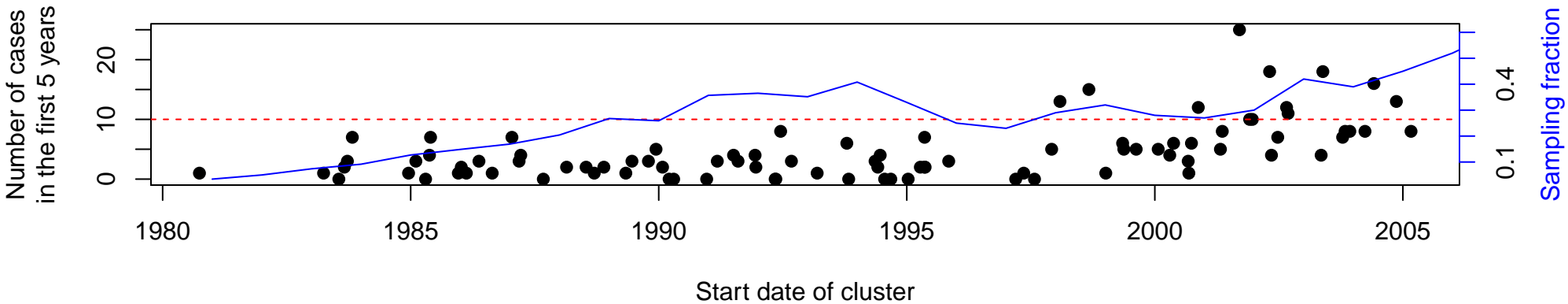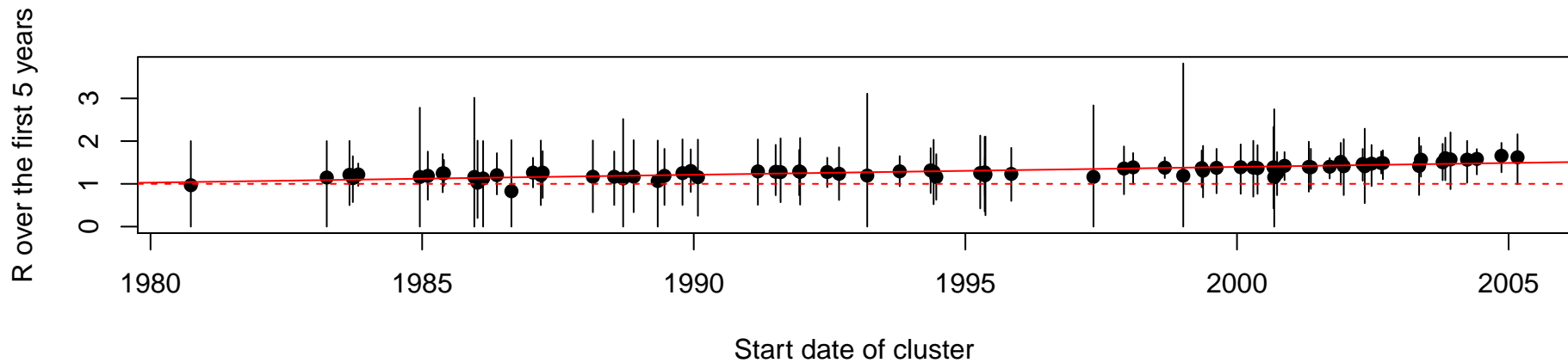

Supplement: S6 Fig — (A) Number of cases in the first 5 y of each cluster and overall sample fraction. (B) Rate of increase in the mean estimated R t over the first 5 y for each cluster. (PDF) [file pmed.1001898.s006.pdf]

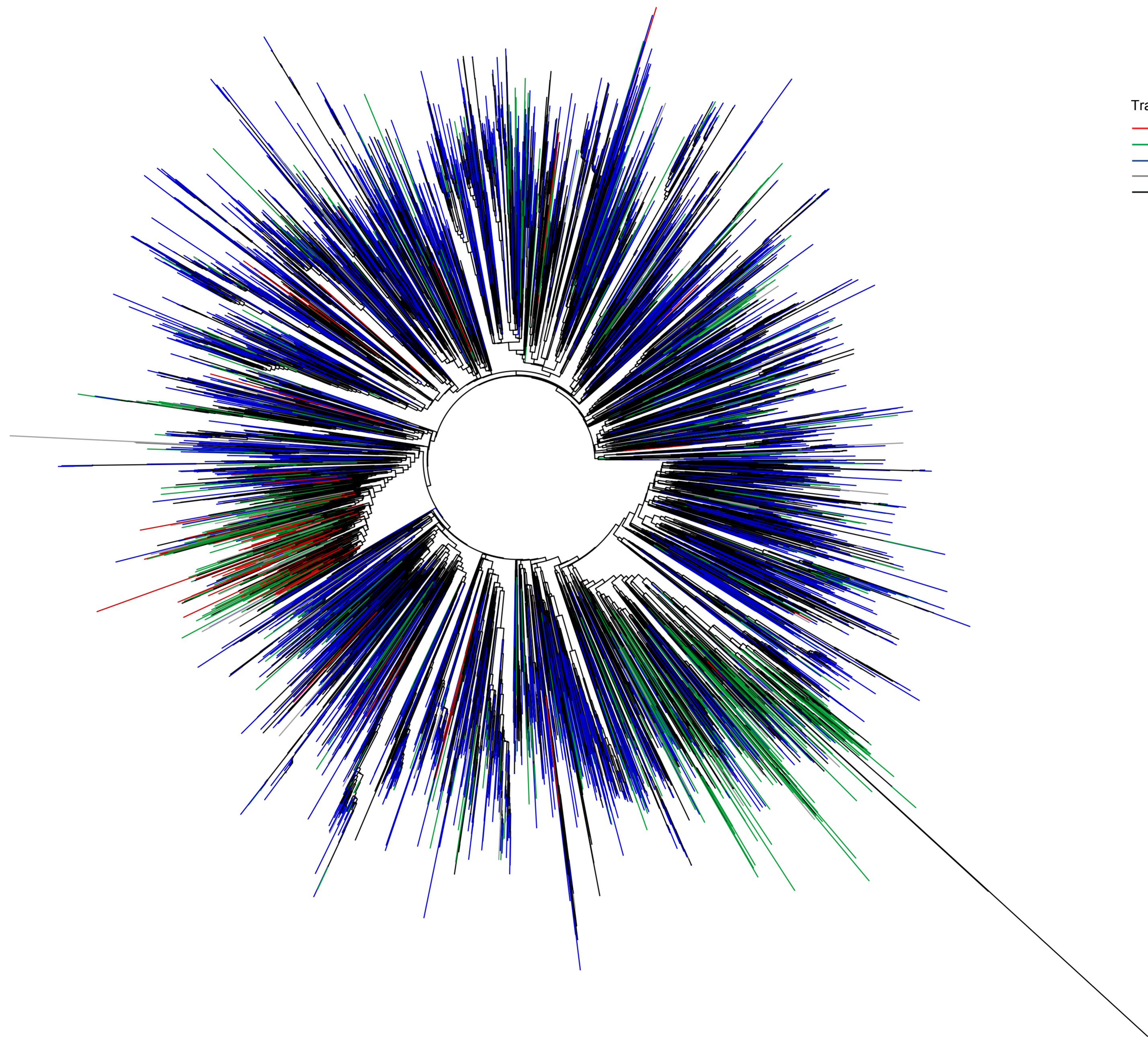

Transmission risk group

- PWID
- HT
- MSM
- Other
- Los Alamos

0.03 mutations / nucleotide site

Supplement: S7 Fig — Branches corresponding to the 5,852 ATHENA patients are colored by self-reported risk group of infection. HT, heterosexual transmission. (PDF) [file pmed.1001898.s007.pdf]

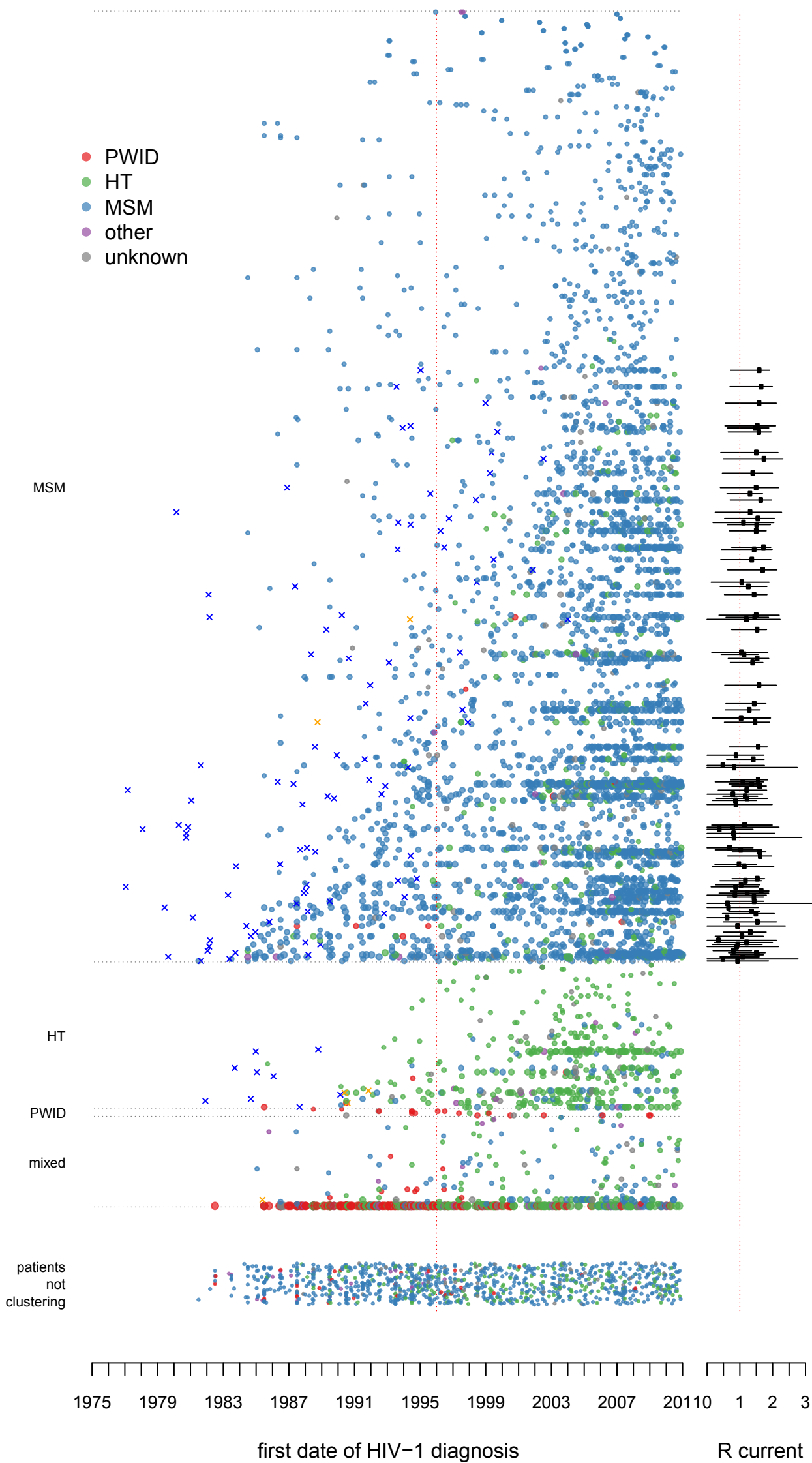

Supplement: S8 Fig — As in Fig 2A, but including the small clusters including 2–9 ATHENA sequences sorted by duration. (PDF) [file pmed.1001898.s008.pdf]

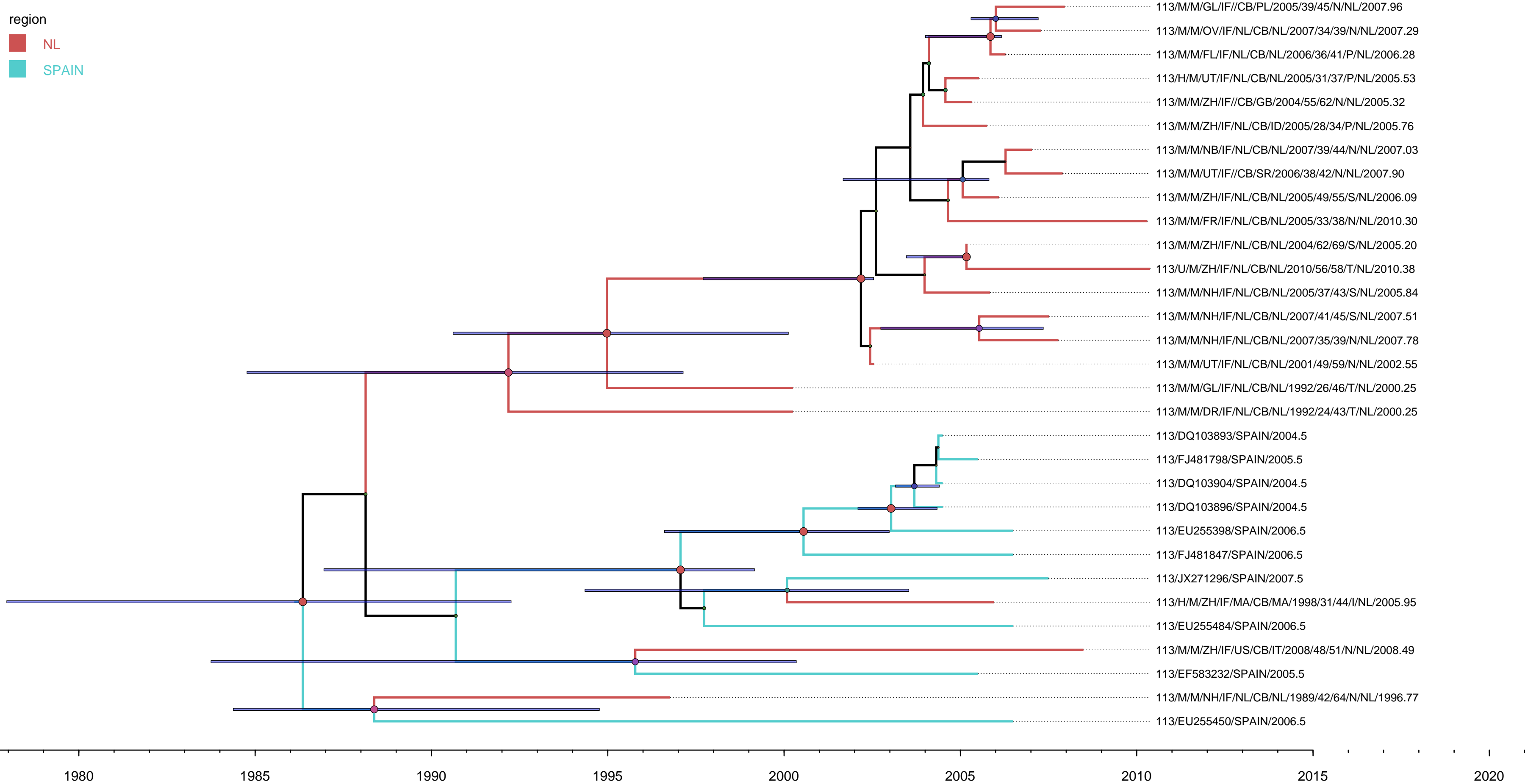

Supplement: S1 Trees — The first number of the branch name is the cluster name. Bars indicate the 95% HPD intervals of the node ages. In total, 380 of the Los Alamos sequences selected are included in the 91 large MSM-majority clusters. Twenty-six clusters have no Los Alamos sequences included. Nine clusters include ≥5 time-stamped sequences from a particular country (Foreign). Although some mixing is visible, these sequences form mainly separate clusters within the respective countries. Timed tree of the 15 other clusters (Other): four clusters with a majority of patients of Suriname origin, three clusters with a majority of patients of Antillean origin, two clusters of heterosexually infected individuals and MSM of mainly Dutch origin, one cluster with mainly PWID of Polish origin, the largest PWID cluster, and the six clusters on Curaçao with bars (two are also in MSM). (ZIP) [file pmed.1001898.s014.zip › S1_Trees/Foreign/MSMcluster113withSpain.pdf]

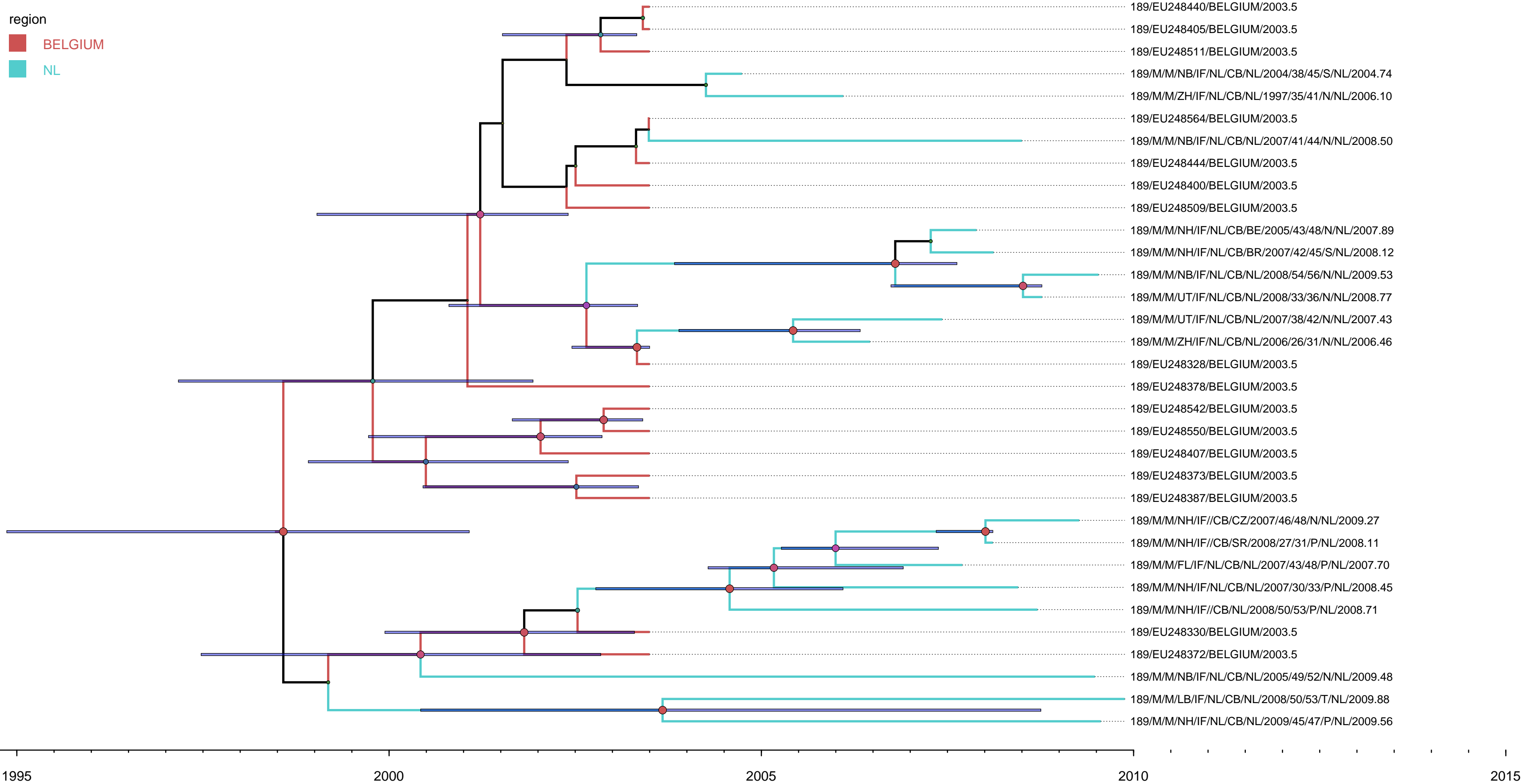

Supplement: S1 Trees — The first number of the branch name is the cluster name. Bars indicate the 95% HPD intervals of the node ages. In total, 380 of the Los Alamos sequences selected are included in the 91 large MSM-majority clusters. Twenty-six clusters have no Los Alamos sequences included. Nine clusters include ≥5 time-stamped sequences from a particular country (Foreign). Although some mixing is visible, these sequences form mainly separate clusters within the respective countries. Timed tree of the 15 other clusters (Other): four clusters with a majority of patients of Suriname origin, three clusters with a majority of patients of Antillean origin, two clusters of heterosexually infected individuals and MSM of mainly Dutch origin, one cluster with mainly PWID of Polish origin, the largest PWID cluster, and the six clusters on Curaçao with bars (two are also in MSM). (ZIP) [file pmed.1001898.s014.zip › S1_Trees/Foreign/MSMcluster189withBelgium.pdf]

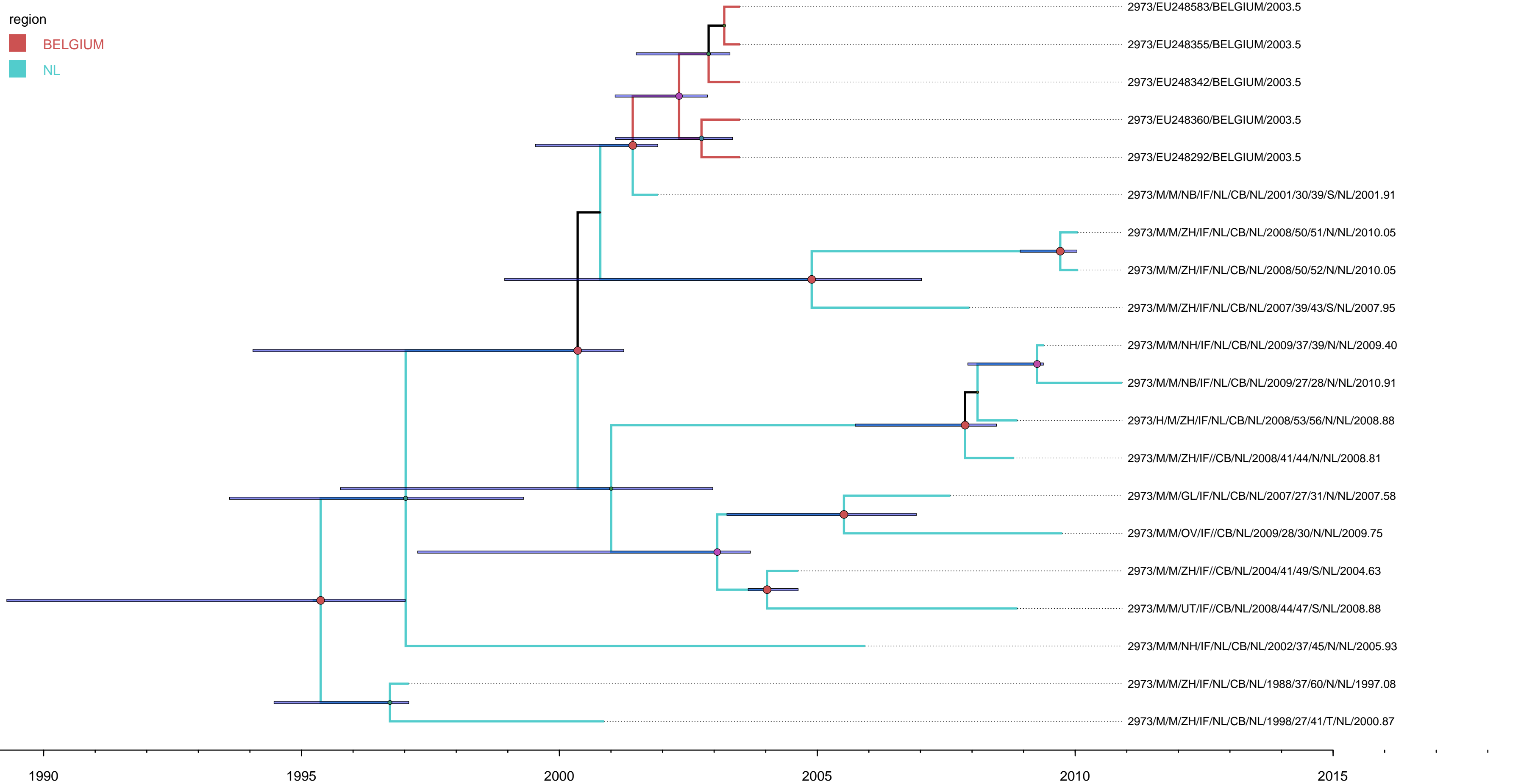

Supplement: S1 Trees — The first number of the branch name is the cluster name. Bars indicate the 95% HPD intervals of the node ages. In total, 380 of the Los Alamos sequences selected are included in the 91 large MSM-majority clusters. Twenty-six clusters have no Los Alamos sequences included. Nine clusters include ≥5 time-stamped sequences from a particular country (Foreign). Although some mixing is visible, these sequences form mainly separate clusters within the respective countries. Timed tree of the 15 other clusters (Other): four clusters with a majority of patients of Suriname origin, three clusters with a majority of patients of Antillean origin, two clusters of heterosexually infected individuals and MSM of mainly Dutch origin, one cluster with mainly PWID of Polish origin, the largest PWID cluster, and the six clusters on Curaçao with bars (two are also in MSM). (ZIP) [file pmed.1001898.s014.zip › S1_Trees/Foreign/MSMcluster2973withBelgium.pdf]

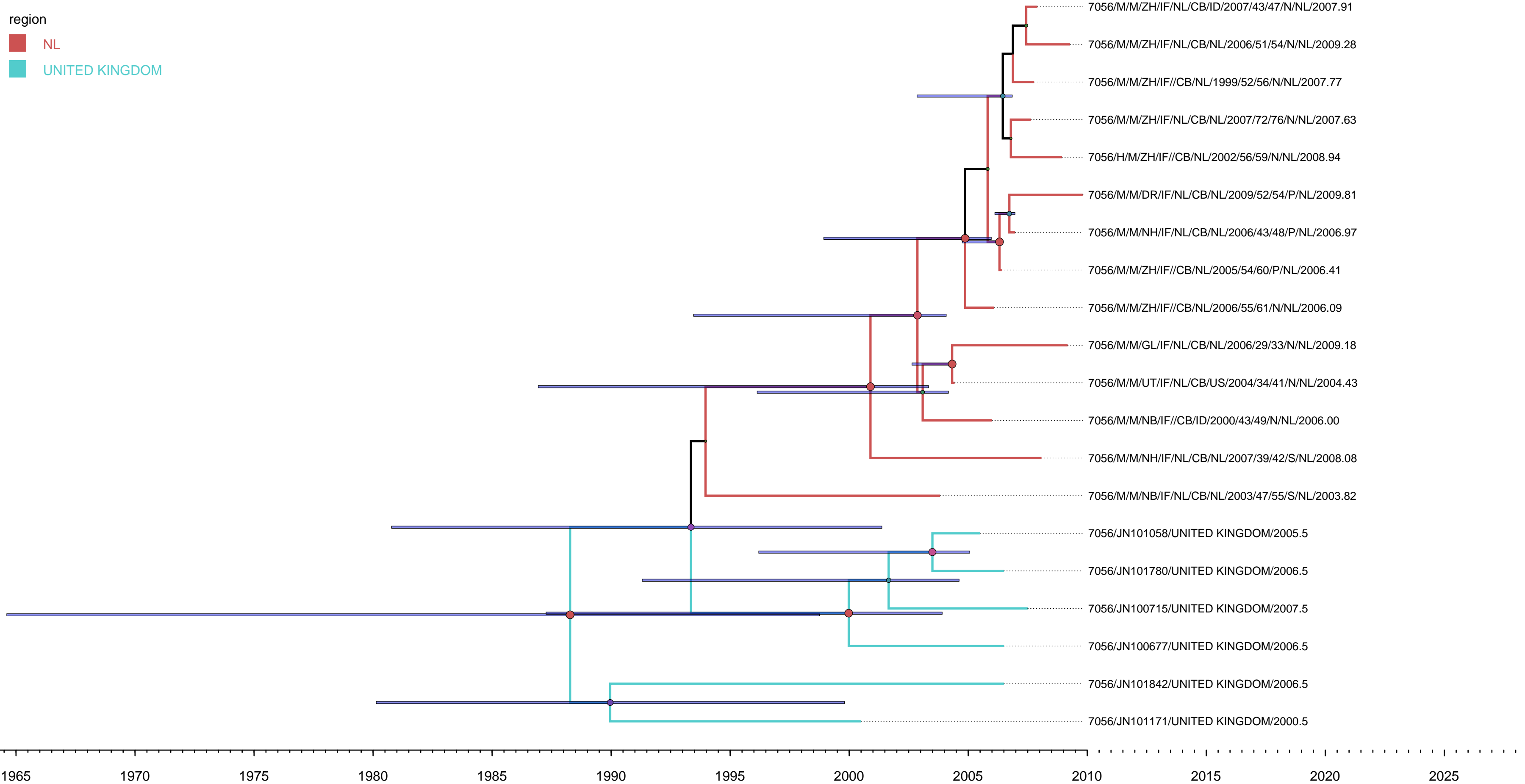

Supplement: S1 Trees — The first number of the branch name is the cluster name. Bars indicate the 95% HPD intervals of the node ages. In total, 380 of the Los Alamos sequences selected are included in the 91 large MSM-majority clusters. Twenty-six clusters have no Los Alamos sequences included. Nine clusters include ≥5 time-stamped sequences from a particular country (Foreign). Although some mixing is visible, these sequences form mainly separate clusters within the respective countries. Timed tree of the 15 other clusters (Other): four clusters with a majority of patients of Suriname origin, three clusters with a majority of patients of Antillean origin, two clusters of heterosexually infected individuals and MSM of mainly Dutch origin, one cluster with mainly PWID of Polish origin, the largest PWID cluster, and the six clusters on Curaçao with bars (two are also in MSM). (ZIP) [file pmed.1001898.s014.zip › S1_Trees/Foreign/MSMcluster7056withUK.pdf]

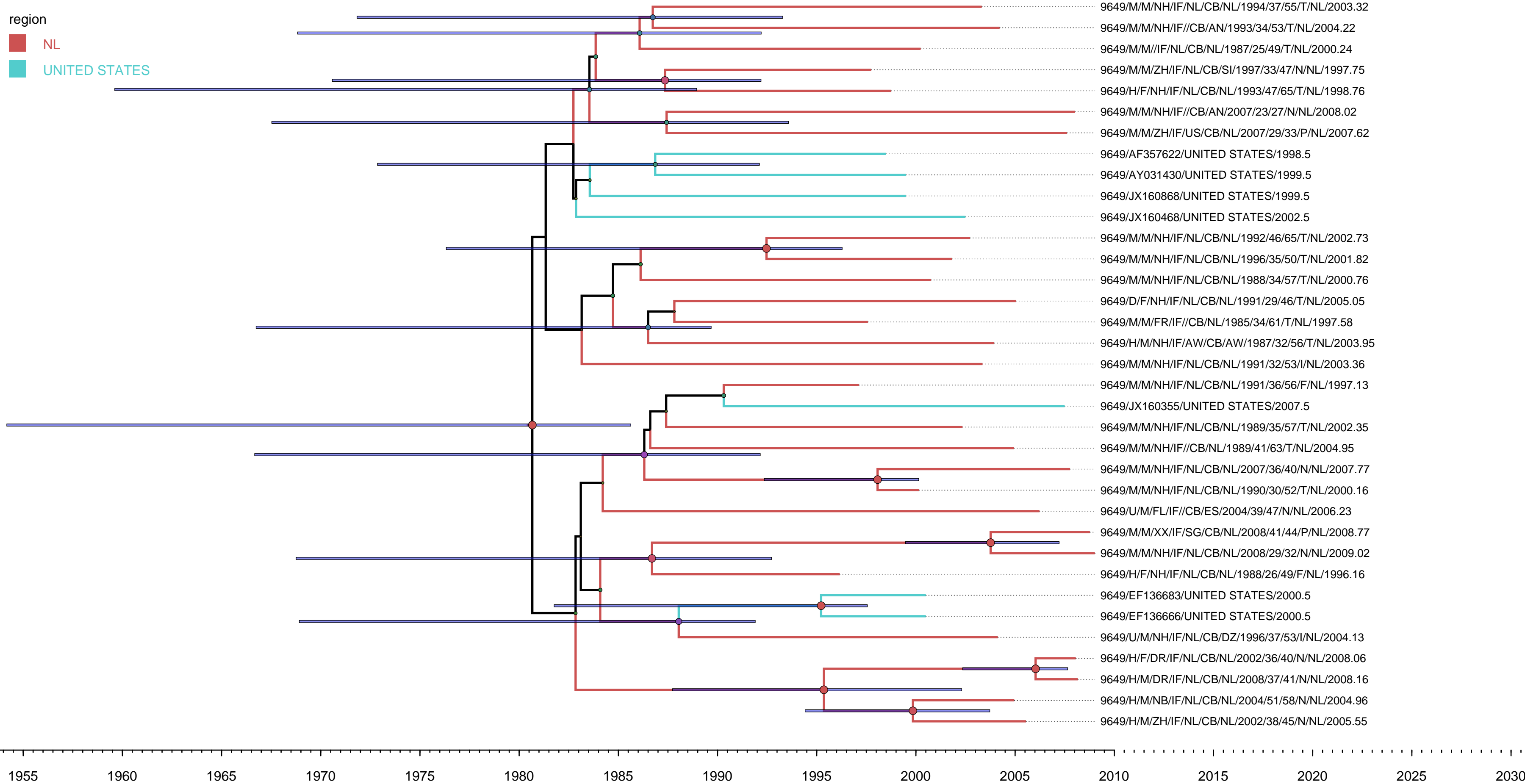

Supplement: S1 Trees — The first number of the branch name is the cluster name. Bars indicate the 95% HPD intervals of the node ages. In total, 380 of the Los Alamos sequences selected are included in the 91 large MSM-majority clusters. Twenty-six clusters have no Los Alamos sequences included. Nine clusters include ≥5 time-stamped sequences from a particular country (Foreign). Although some mixing is visible, these sequences form mainly separate clusters within the respective countries. Timed tree of the 15 other clusters (Other): four clusters with a majority of patients of Suriname origin, three clusters with a majority of patients of Antillean origin, two clusters of heterosexually infected individuals and MSM of mainly Dutch origin, one cluster with mainly PWID of Polish origin, the largest PWID cluster, and the six clusters on Curaçao with bars (two are also in MSM). (ZIP) [file pmed.1001898.s014.zip › S1_Trees/Foreign/MSMcluster9649withUS.pdf]

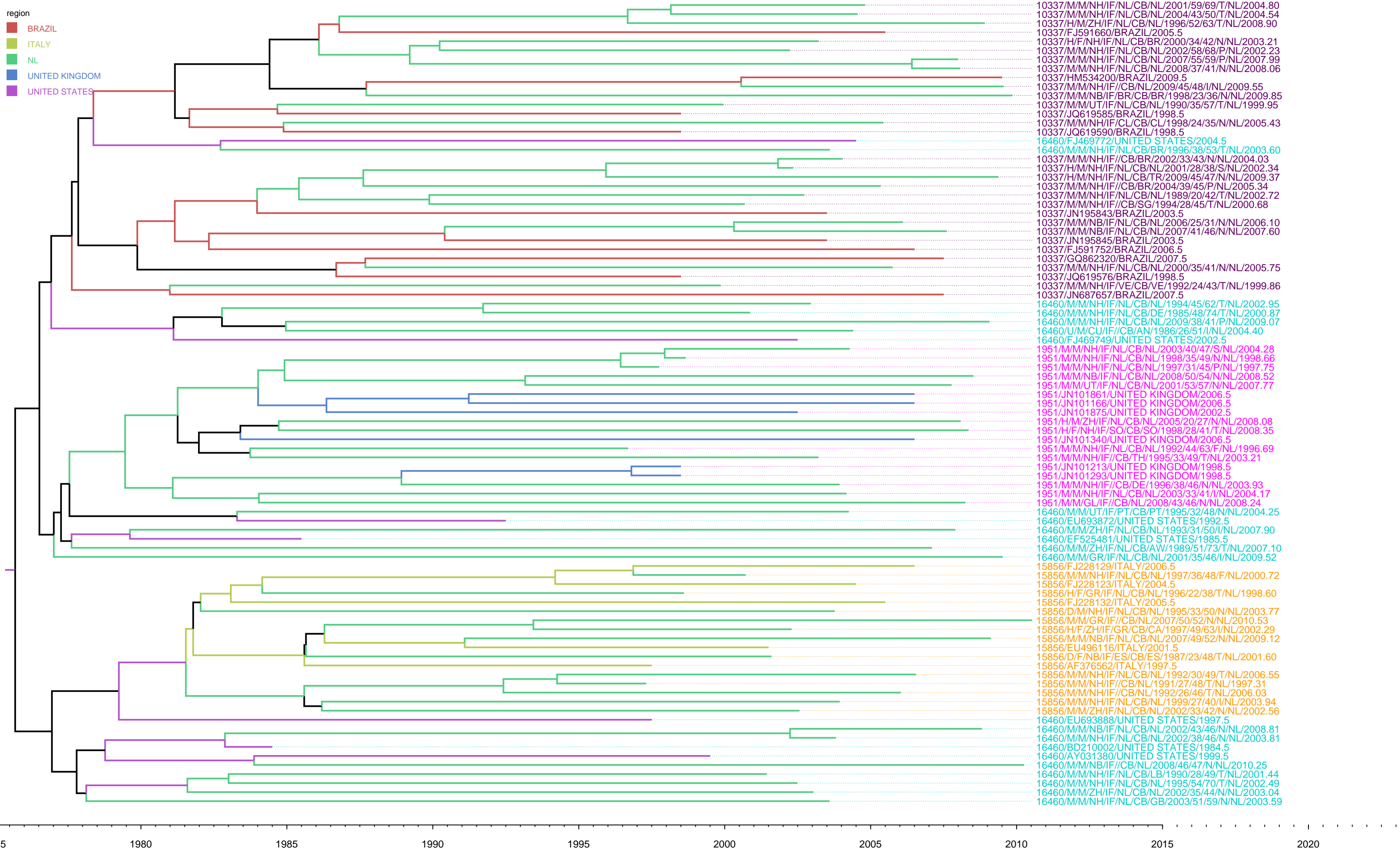

Supplement: S1 Trees — The first number of the branch name is the cluster name. Bars indicate the 95% HPD intervals of the node ages. In total, 380 of the Los Alamos sequences selected are included in the 91 large MSM-majority clusters. Twenty-six clusters have no Los Alamos sequences included. Nine clusters include ≥5 time-stamped sequences from a particular country (Foreign). Although some mixing is visible, these sequences form mainly separate clusters within the respective countries. Timed tree of the 15 other clusters (Other): four clusters with a majority of patients of Suriname origin, three clusters with a majority of patients of Antillean origin, two clusters of heterosexually infected individuals and MSM of mainly Dutch origin, one cluster with mainly PWID of Polish origin, the largest PWID cluster, and the six clusters on Curaçao with bars (two are also in MSM). (ZIP) [file pmed.1001898.s014.zip › S1_Trees/Foreign/MSMclusters4xforeign_rootprior.pdf]

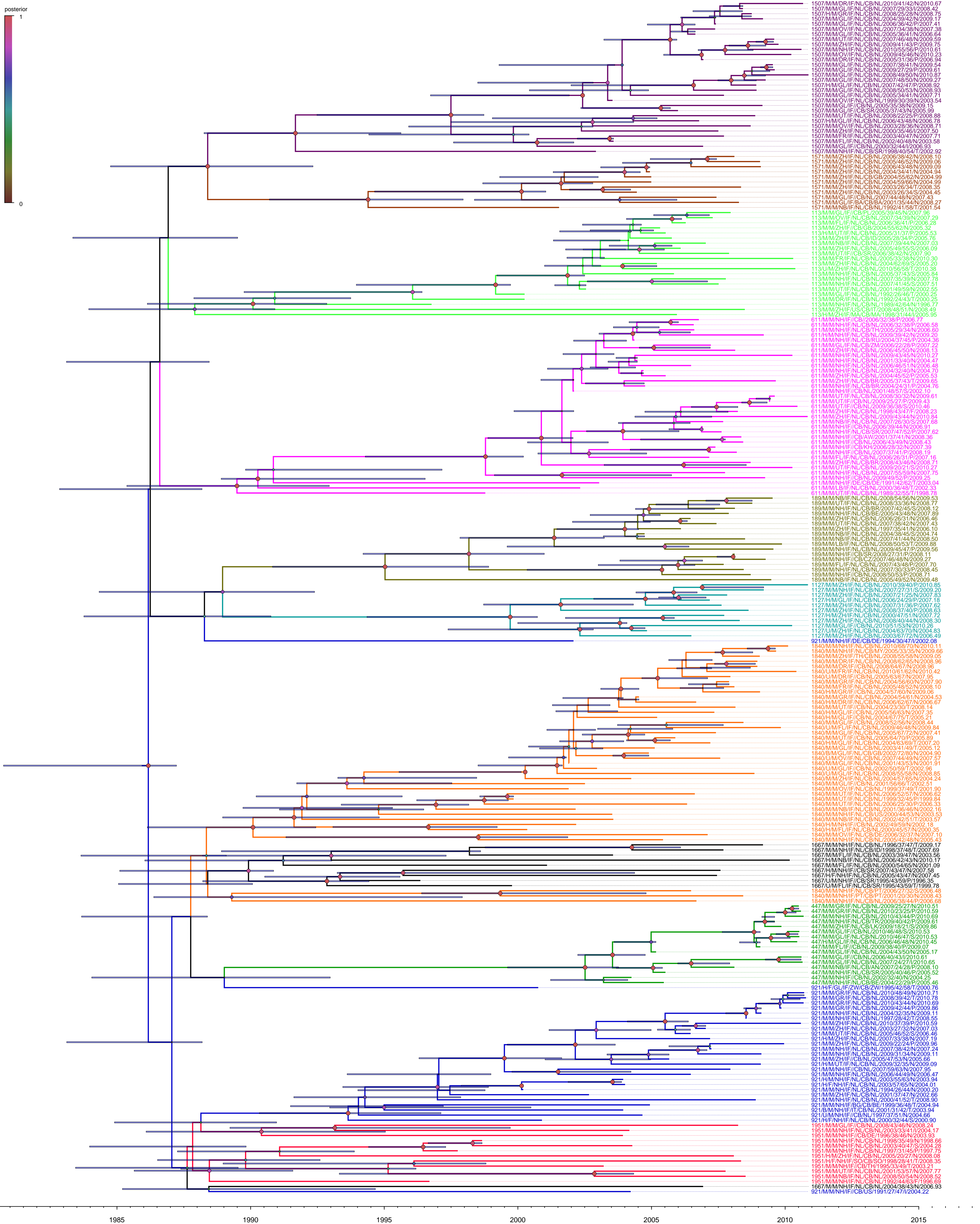

Supplement: S1 Trees — The first number of the branch name is the cluster name. Bars indicate the 95% HPD intervals of the node ages. In total, 380 of the Los Alamos sequences selected are included in the 91 large MSM-majority clusters. Twenty-six clusters have no Los Alamos sequences included. Nine clusters include ≥5 time-stamped sequences from a particular country (Foreign). Although some mixing is visible, these sequences form mainly separate clusters within the respective countries. Timed tree of the 15 other clusters (Other): four clusters with a majority of patients of Suriname origin, three clusters with a majority of patients of Antillean origin, two clusters of heterosexually infected individuals and MSM of mainly Dutch origin, one cluster with mainly PWID of Polish origin, the largest PWID cluster, and the six clusters on Curaçao with bars (two are also in MSM). (ZIP) [file pmed.1001898.s014.zip › S1_Trees/MSM/MSM1.pdf]

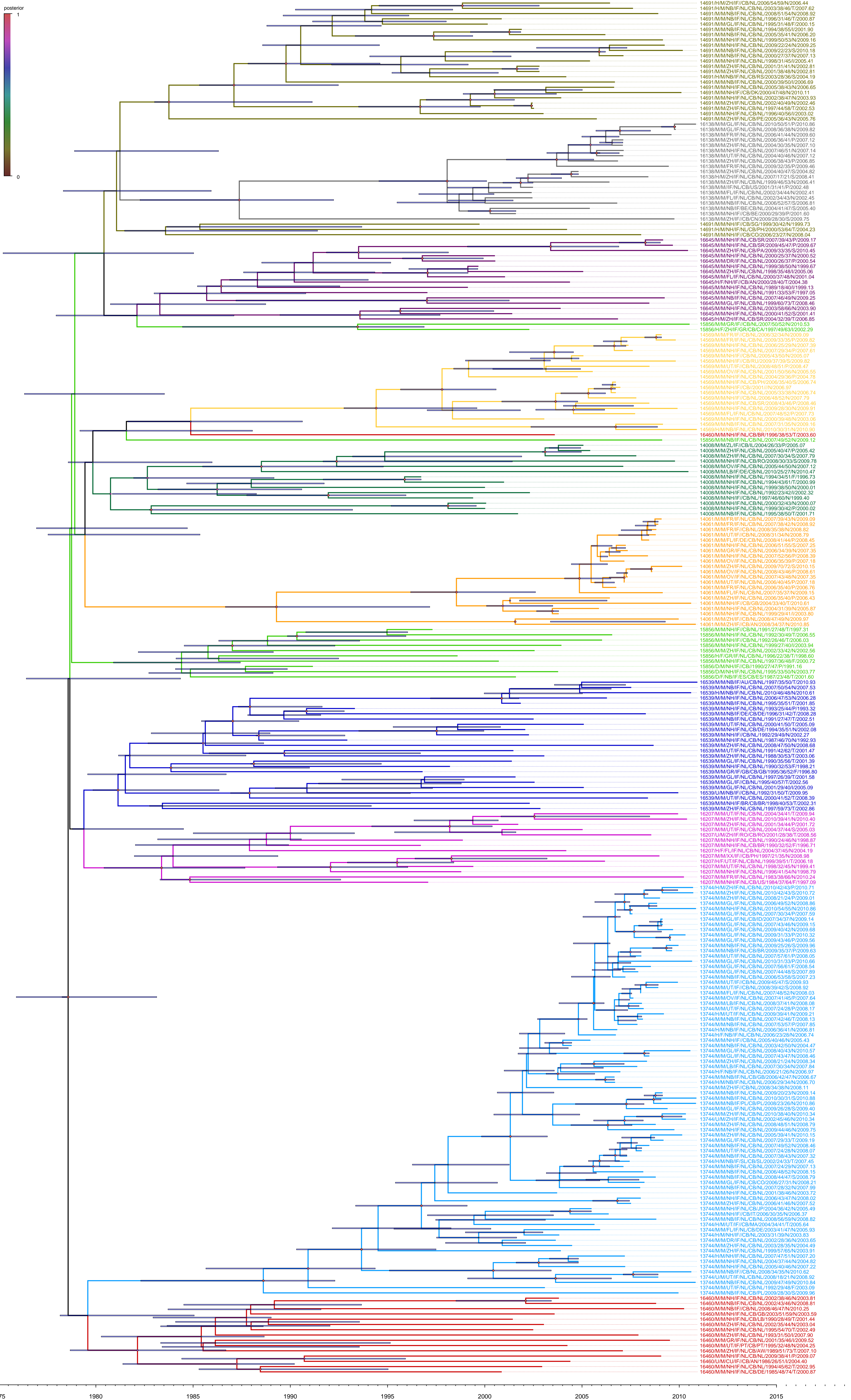

Supplement: S1 Trees — The first number of the branch name is the cluster name. Bars indicate the 95% HPD intervals of the node ages. In total, 380 of the Los Alamos sequences selected are included in the 91 large MSM-majority clusters. Twenty-six clusters have no Los Alamos sequences included. Nine clusters include ≥5 time-stamped sequences from a particular country (Foreign). Although some mixing is visible, these sequences form mainly separate clusters within the respective countries. Timed tree of the 15 other clusters (Other): four clusters with a majority of patients of Suriname origin, three clusters with a majority of patients of Antillean origin, two clusters of heterosexually infected individuals and MSM of mainly Dutch origin, one cluster with mainly PWID of Polish origin, the largest PWID cluster, and the six clusters on Curaçao with bars (two are also in MSM). (ZIP) [file pmed.1001898.s014.zip › S1_Trees/MSM/MSM10.pdf]

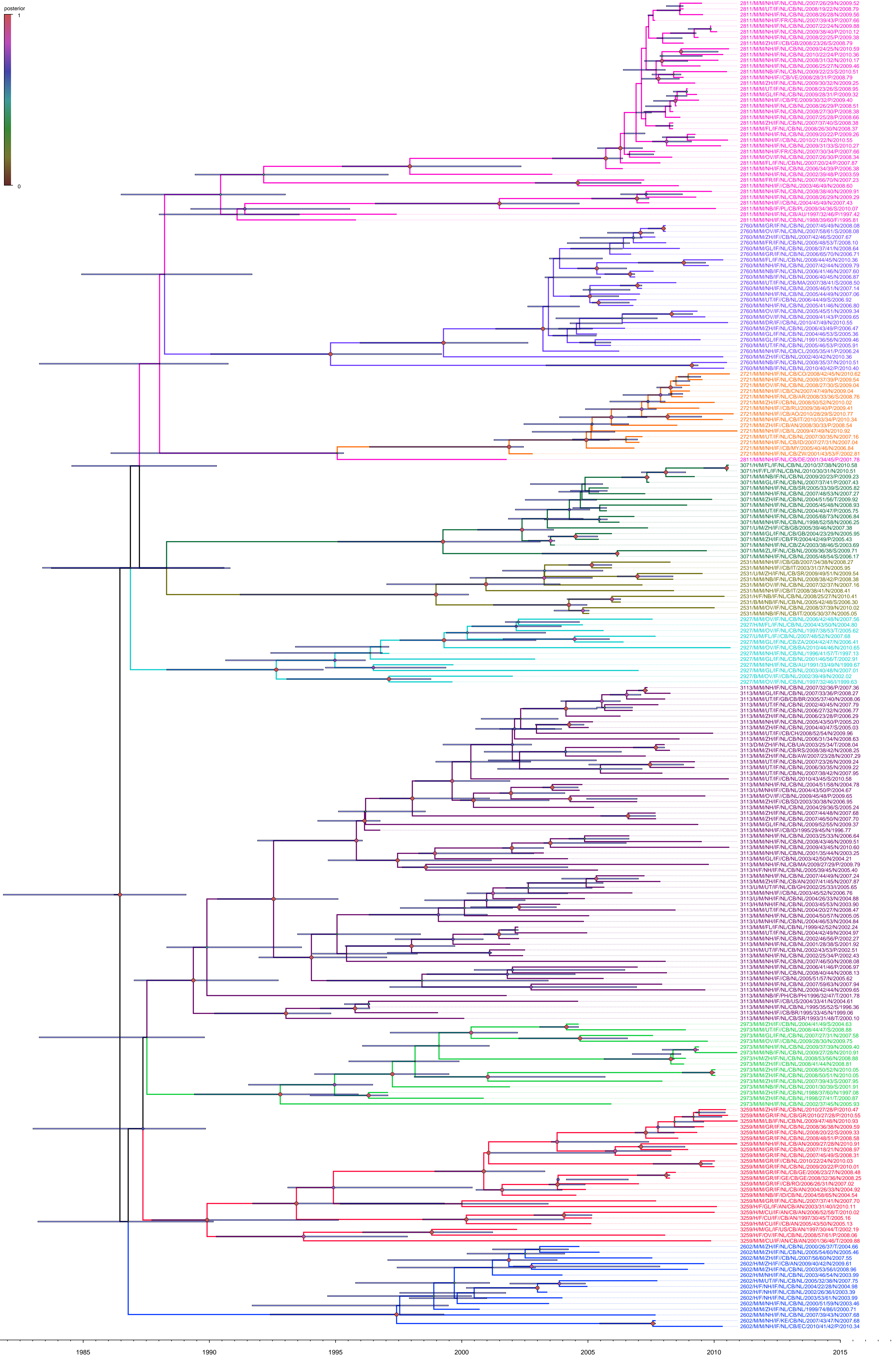

Supplement: S1 Trees — The first number of the branch name is the cluster name. Bars indicate the 95% HPD intervals of the node ages. In total, 380 of the Los Alamos sequences selected are included in the 91 large MSM-majority clusters. Twenty-six clusters have no Los Alamos sequences included. Nine clusters include ≥5 time-stamped sequences from a particular country (Foreign). Although some mixing is visible, these sequences form mainly separate clusters within the respective countries. Timed tree of the 15 other clusters (Other): four clusters with a majority of patients of Suriname origin, three clusters with a majority of patients of Antillean origin, two clusters of heterosexually infected individuals and MSM of mainly Dutch origin, one cluster with mainly PWID of Polish origin, the largest PWID cluster, and the six clusters on Curaçao with bars (two are also in MSM). (ZIP) [file pmed.1001898.s014.zip › S1_Trees/MSM/MSM2.pdf]

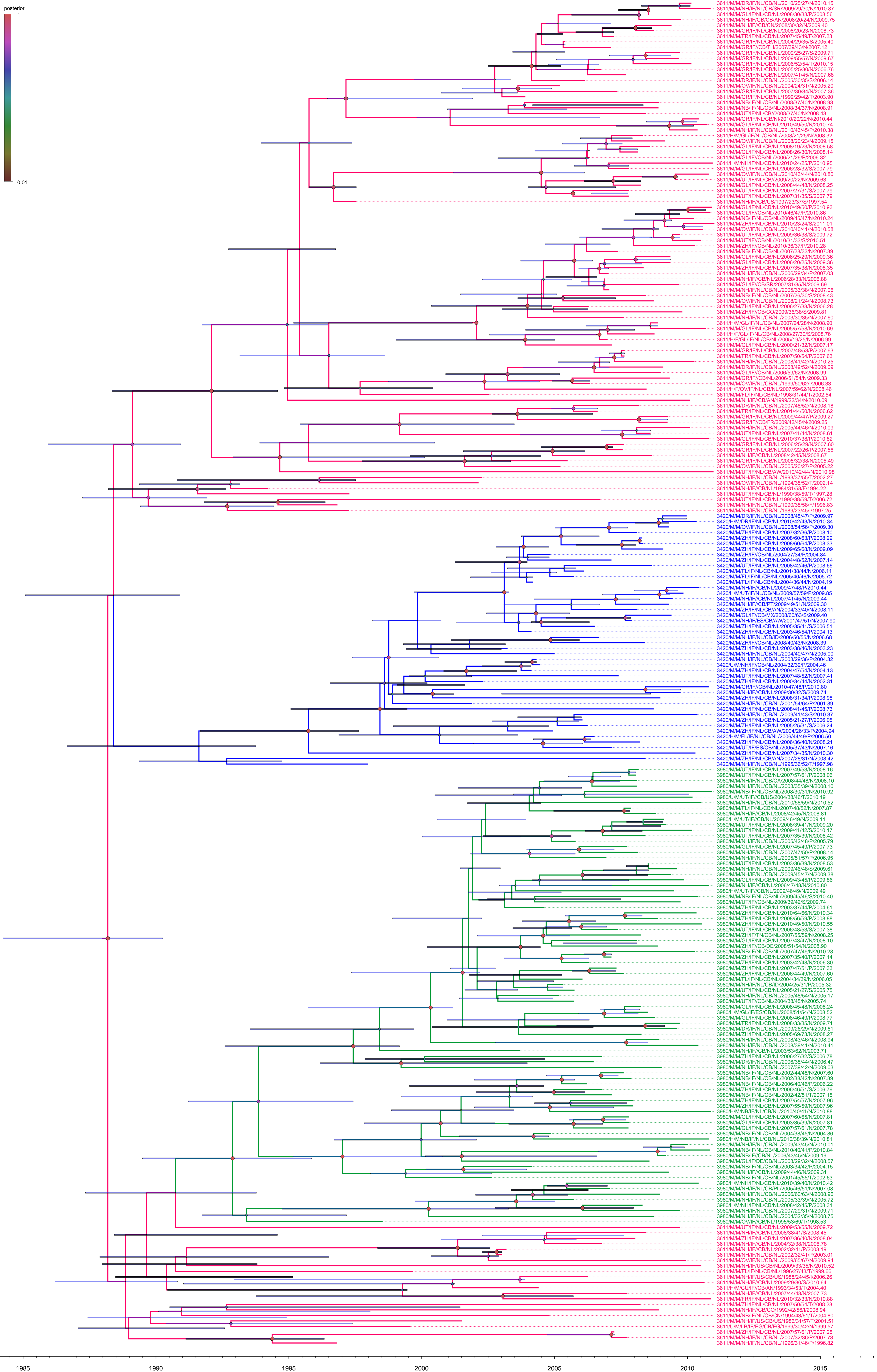

Supplement: S1 Trees — The first number of the branch name is the cluster name. Bars indicate the 95% HPD intervals of the node ages. In total, 380 of the Los Alamos sequences selected are included in the 91 large MSM-majority clusters. Twenty-six clusters have no Los Alamos sequences included. Nine clusters include ≥5 time-stamped sequences from a particular country (Foreign). Although some mixing is visible, these sequences form mainly separate clusters within the respective countries. Timed tree of the 15 other clusters (Other): four clusters with a majority of patients of Suriname origin, three clusters with a majority of patients of Antillean origin, two clusters of heterosexually infected individuals and MSM of mainly Dutch origin, one cluster with mainly PWID of Polish origin, the largest PWID cluster, and the six clusters on Curaçao with bars (two are also in MSM). (ZIP) [file pmed.1001898.s014.zip › S1_Trees/MSM/MSM3.pdf]

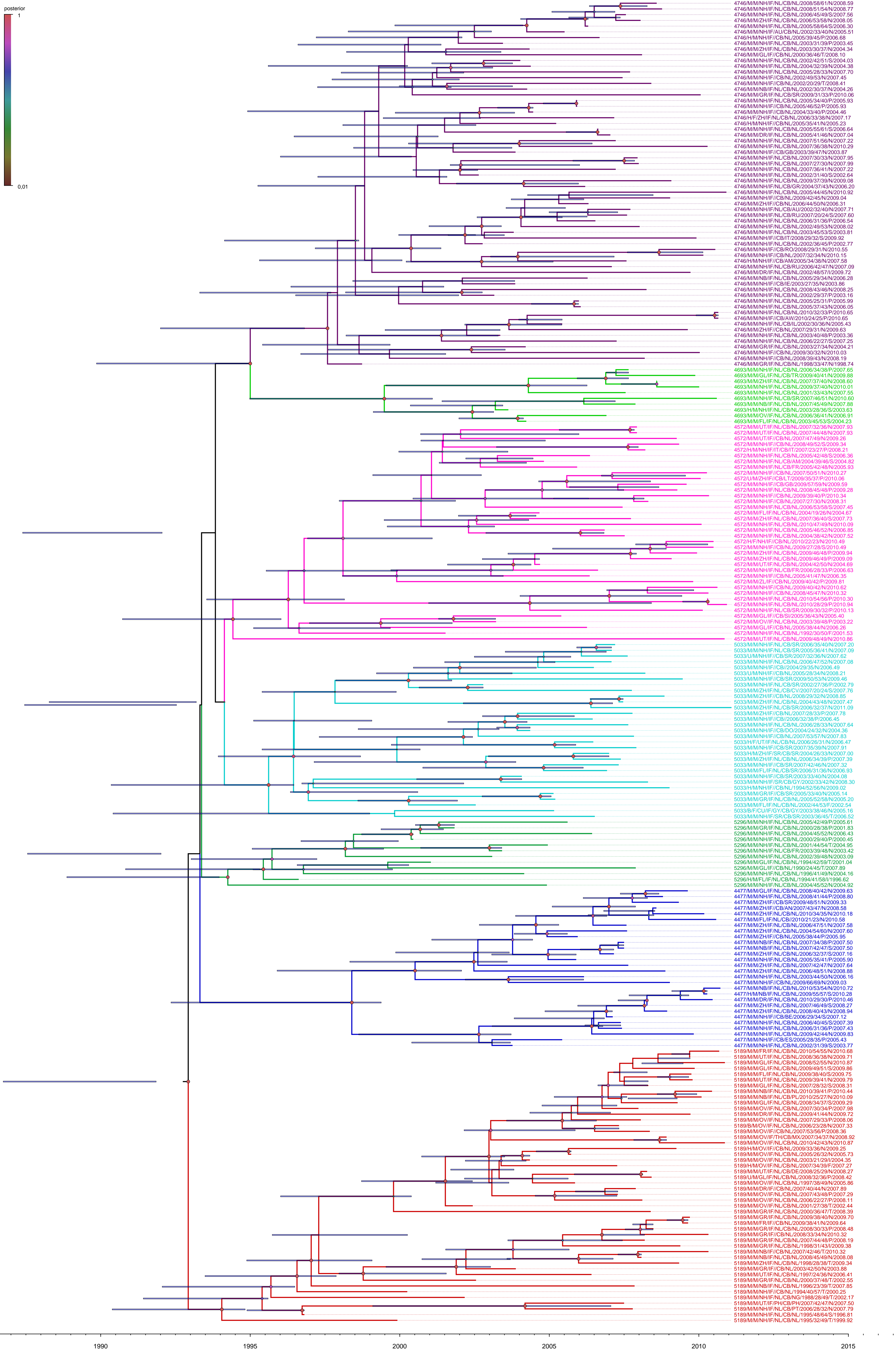

Supplement: S1 Trees — The first number of the branch name is the cluster name. Bars indicate the 95% HPD intervals of the node ages. In total, 380 of the Los Alamos sequences selected are included in the 91 large MSM-majority clusters. Twenty-six clusters have no Los Alamos sequences included. Nine clusters include ≥5 time-stamped sequences from a particular country (Foreign). Although some mixing is visible, these sequences form mainly separate clusters within the respective countries. Timed tree of the 15 other clusters (Other): four clusters with a majority of patients of Suriname origin, three clusters with a majority of patients of Antillean origin, two clusters of heterosexually infected individuals and MSM of mainly Dutch origin, one cluster with mainly PWID of Polish origin, the largest PWID cluster, and the six clusters on Curaçao with bars (two are also in MSM). (ZIP) [file pmed.1001898.s014.zip › S1_Trees/MSM/MSM4.pdf]

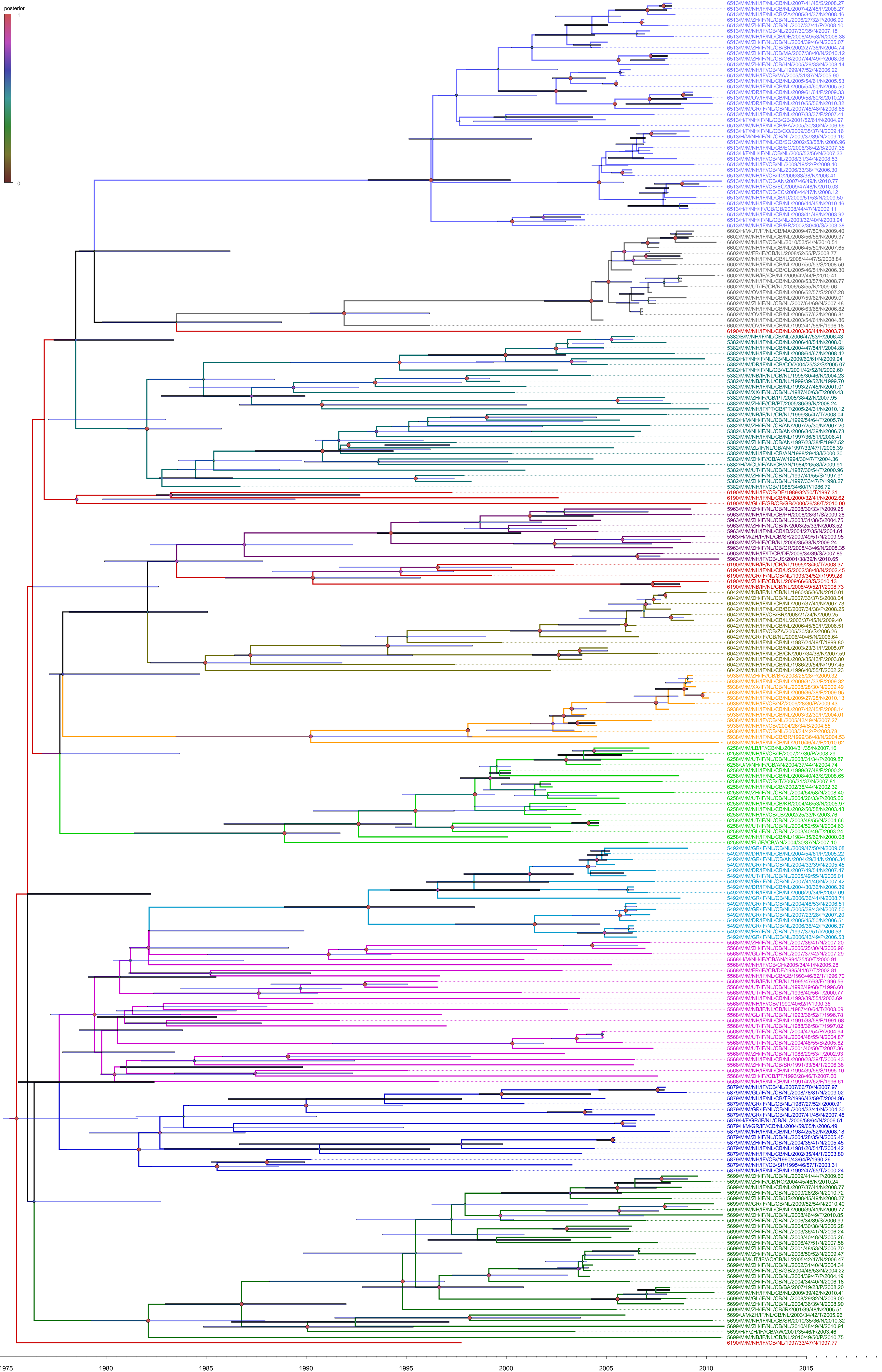

Supplement: S1 Trees — The first number of the branch name is the cluster name. Bars indicate the 95% HPD intervals of the node ages. In total, 380 of the Los Alamos sequences selected are included in the 91 large MSM-majority clusters. Twenty-six clusters have no Los Alamos sequences included. Nine clusters include ≥5 time-stamped sequences from a particular country (Foreign). Although some mixing is visible, these sequences form mainly separate clusters within the respective countries. Timed tree of the 15 other clusters (Other): four clusters with a majority of patients of Suriname origin, three clusters with a majority of patients of Antillean origin, two clusters of heterosexually infected individuals and MSM of mainly Dutch origin, one cluster with mainly PWID of Polish origin, the largest PWID cluster, and the six clusters on Curaçao with bars (two are also in MSM). (ZIP) [file pmed.1001898.s014.zip › S1_Trees/MSM/MSM5.pdf]

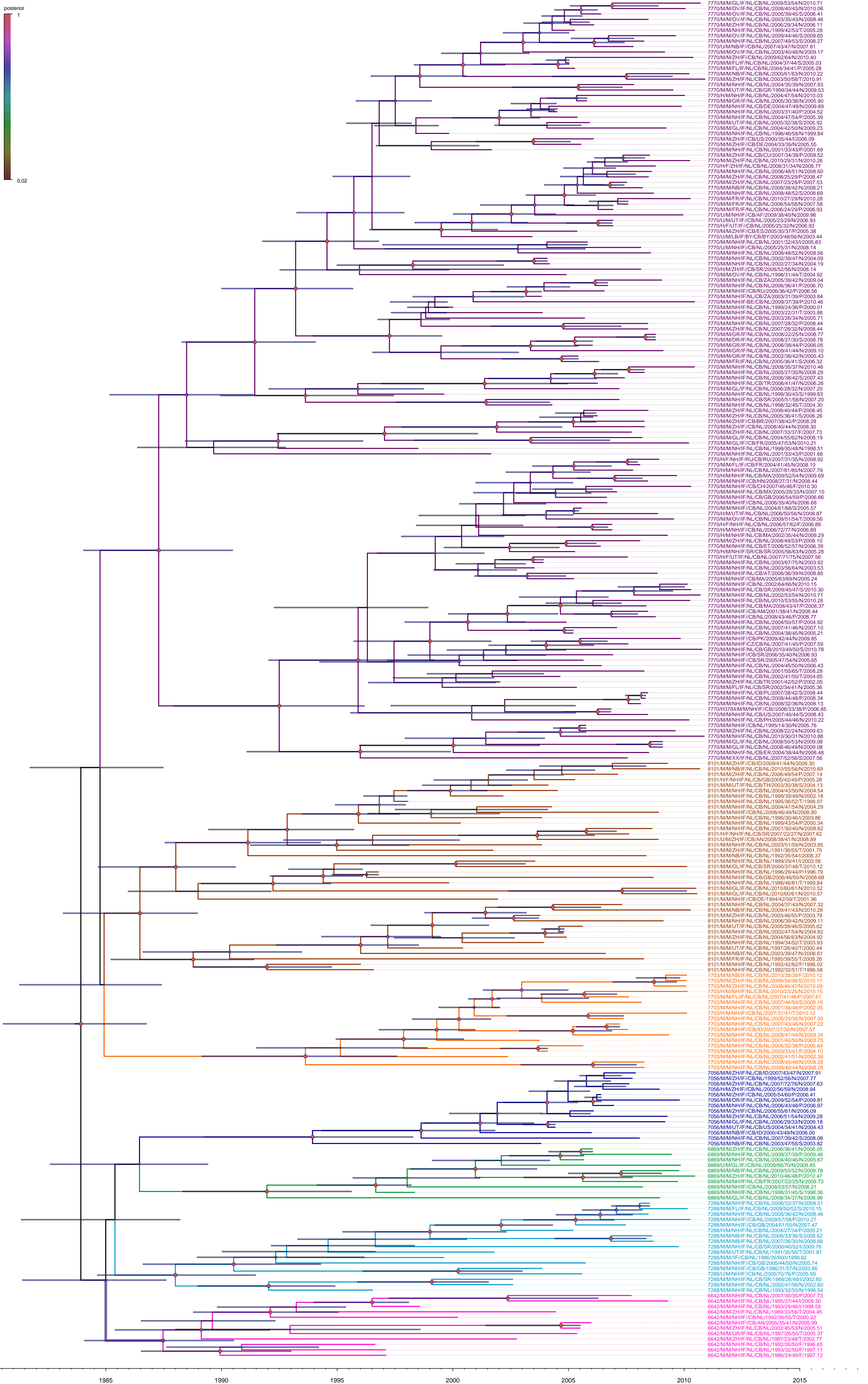

Supplement: S1 Trees — The first number of the branch name is the cluster name. Bars indicate the 95% HPD intervals of the node ages. In total, 380 of the Los Alamos sequences selected are included in the 91 large MSM-majority clusters. Twenty-six clusters have no Los Alamos sequences included. Nine clusters include ≥5 time-stamped sequences from a particular country (Foreign). Although some mixing is visible, these sequences form mainly separate clusters within the respective countries. Timed tree of the 15 other clusters (Other): four clusters with a majority of patients of Suriname origin, three clusters with a majority of patients of Antillean origin, two clusters of heterosexually infected individuals and MSM of mainly Dutch origin, one cluster with mainly PWID of Polish origin, the largest PWID cluster, and the six clusters on Curaçao with bars (two are also in MSM). (ZIP) [file pmed.1001898.s014.zip › S1_Trees/MSM/MSM6.pdf]

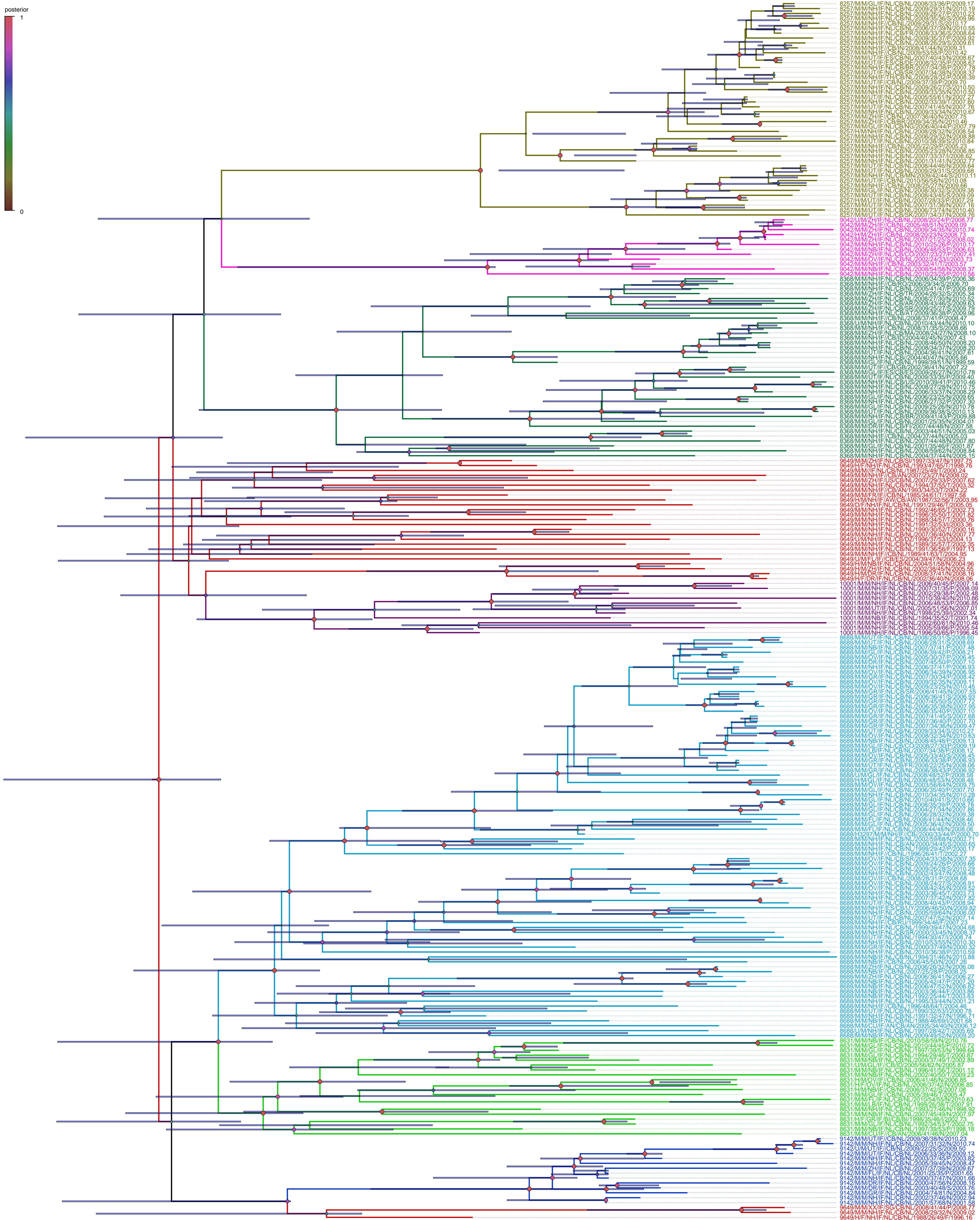

Supplement: S1 Trees — The first number of the branch name is the cluster name. Bars indicate the 95% HPD intervals of the node ages. In total, 380 of the Los Alamos sequences selected are included in the 91 large MSM-majority clusters. Twenty-six clusters have no Los Alamos sequences included. Nine clusters include ≥5 time-stamped sequences from a particular country (Foreign). Although some mixing is visible, these sequences form mainly separate clusters within the respective countries. Timed tree of the 15 other clusters (Other): four clusters with a majority of patients of Suriname origin, three clusters with a majority of patients of Antillean origin, two clusters of heterosexually infected individuals and MSM of mainly Dutch origin, one cluster with mainly PWID of Polish origin, the largest PWID cluster, and the six clusters on Curaçao with bars (two are also in MSM). (ZIP) [file pmed.1001898.s014.zip › S1_Trees/MSM/MSM7.pdf]

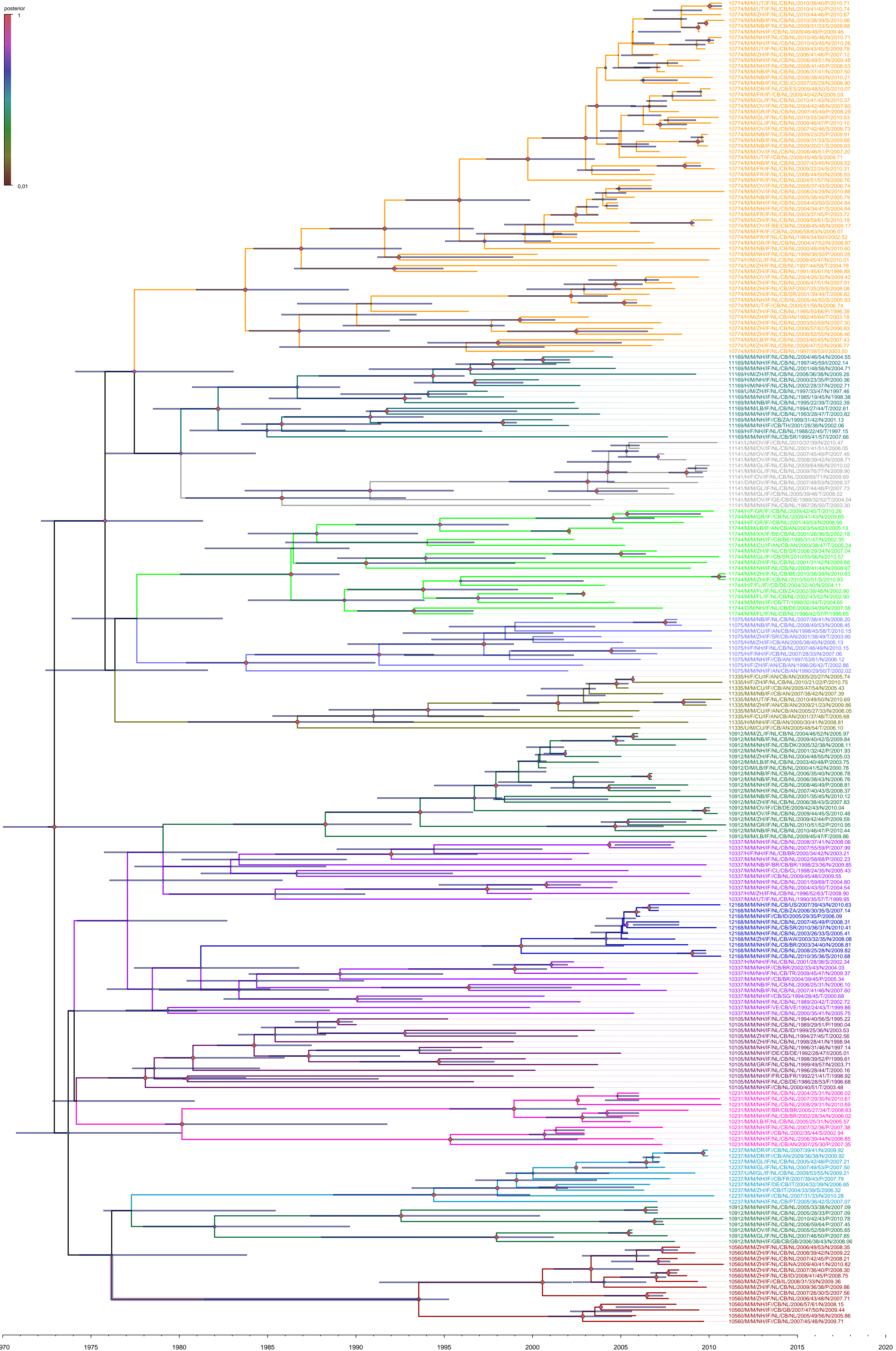

Supplement: S1 Trees — The first number of the branch name is the cluster name. Bars indicate the 95% HPD intervals of the node ages. In total, 380 of the Los Alamos sequences selected are included in the 91 large MSM-majority clusters. Twenty-six clusters have no Los Alamos sequences included. Nine clusters include ≥5 time-stamped sequences from a particular country (Foreign). Although some mixing is visible, these sequences form mainly separate clusters within the respective countries. Timed tree of the 15 other clusters (Other): four clusters with a majority of patients of Suriname origin, three clusters with a majority of patients of Antillean origin, two clusters of heterosexually infected individuals and MSM of mainly Dutch origin, one cluster with mainly PWID of Polish origin, the largest PWID cluster, and the six clusters on Curaçao with bars (two are also in MSM). (ZIP) [file pmed.1001898.s014.zip › S1_Trees/MSM/MSM8.pdf]

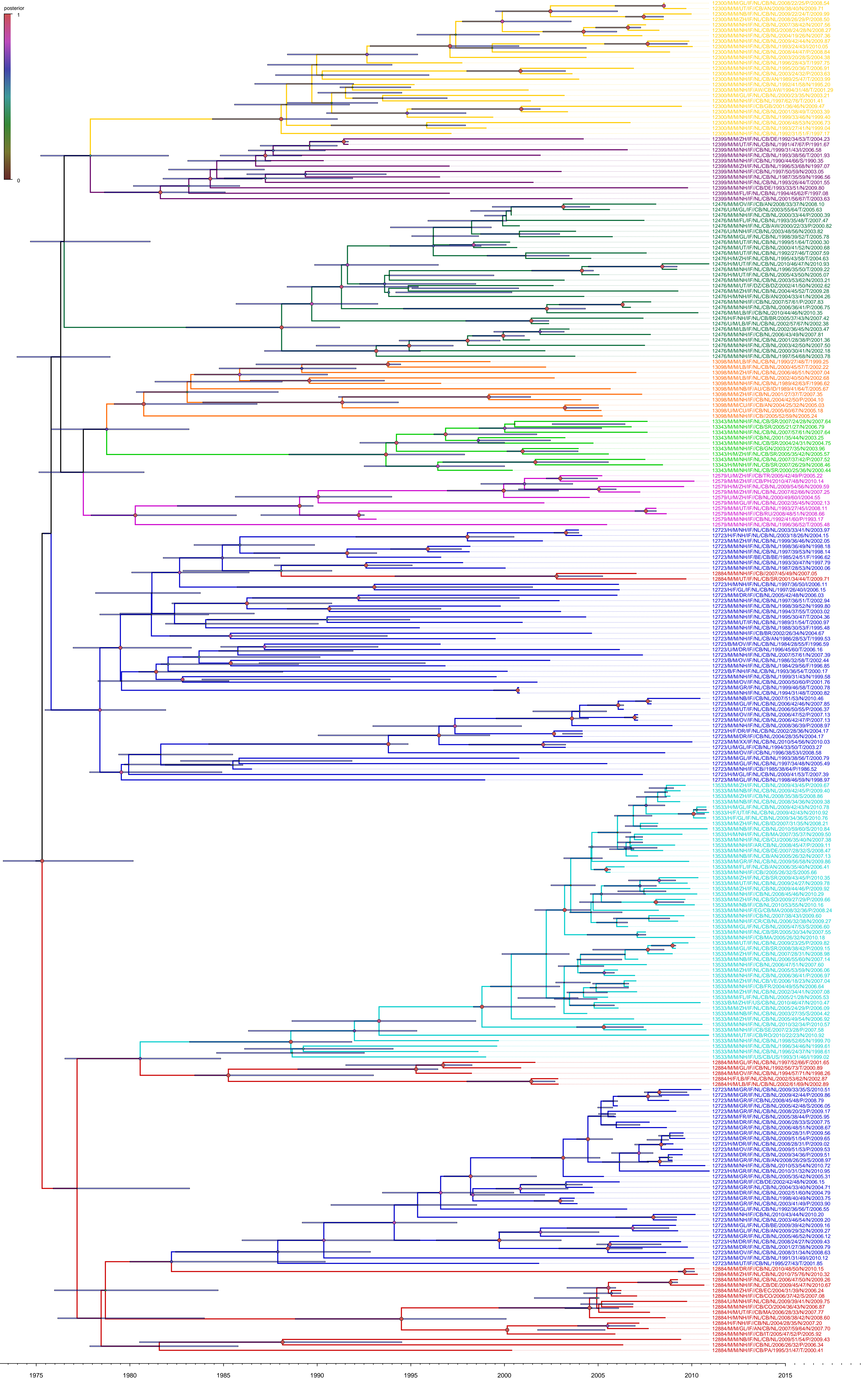

Supplement: S1 Trees — The first number of the branch name is the cluster name. Bars indicate the 95% HPD intervals of the node ages. In total, 380 of the Los Alamos sequences selected are included in the 91 large MSM-majority clusters. Twenty-six clusters have no Los Alamos sequences included. Nine clusters include ≥5 time-stamped sequences from a particular country (Foreign). Although some mixing is visible, these sequences form mainly separate clusters within the respective countries. Timed tree of the 15 other clusters (Other): four clusters with a majority of patients of Suriname origin, three clusters with a majority of patients of Antillean origin, two clusters of heterosexually infected individuals and MSM of mainly Dutch origin, one cluster with mainly PWID of Polish origin, the largest PWID cluster, and the six clusters on Curaçao with bars (two are also in MSM). (ZIP) [file pmed.1001898.s014.zip › S1_Trees/MSM/MSM9.pdf]

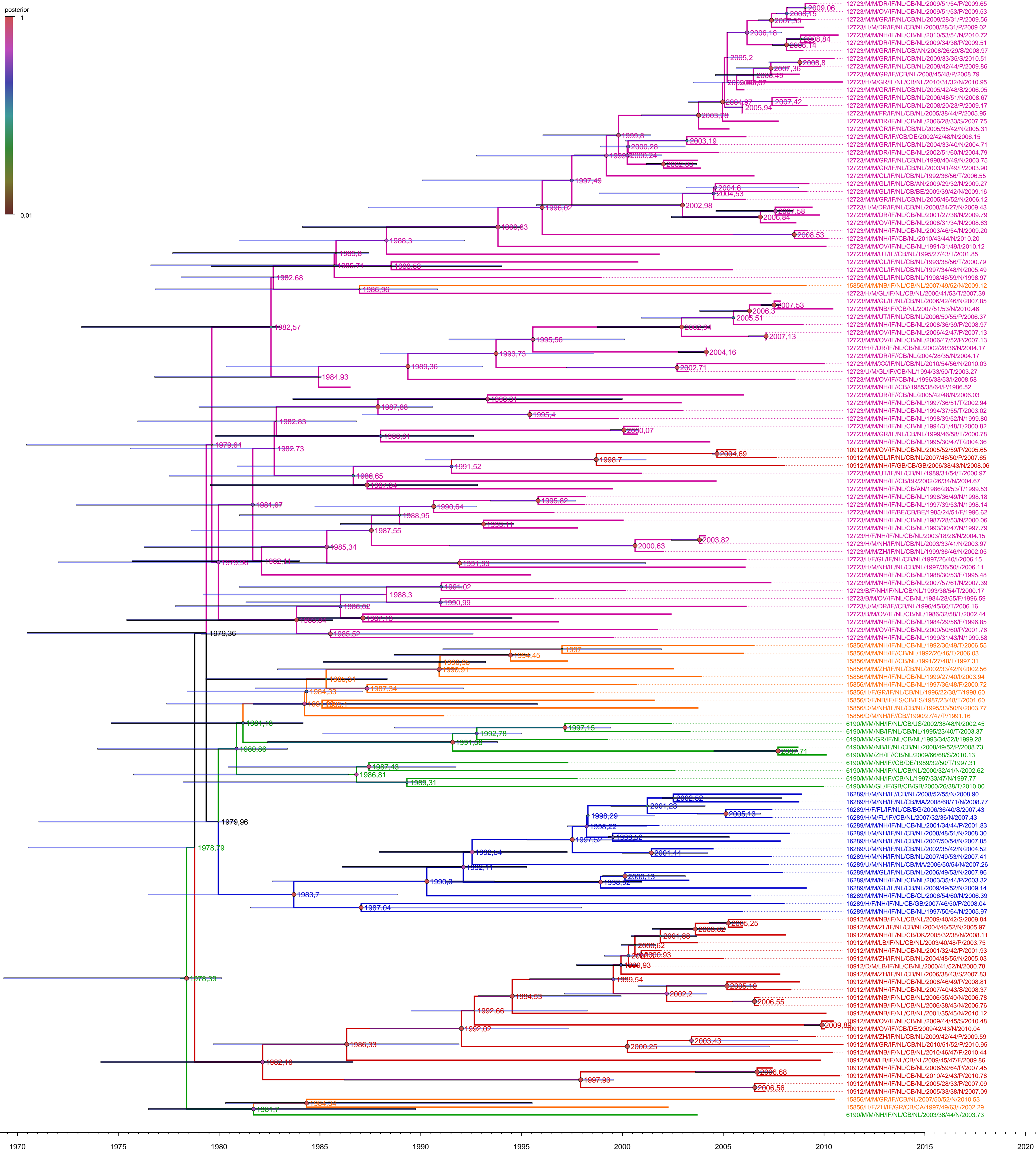

Supplement: S1 Trees — The first number of the branch name is the cluster name. Bars indicate the 95% HPD intervals of the node ages. In total, 380 of the Los Alamos sequences selected are included in the 91 large MSM-majority clusters. Twenty-six clusters have no Los Alamos sequences included. Nine clusters include ≥5 time-stamped sequences from a particular country (Foreign). Although some mixing is visible, these sequences form mainly separate clusters within the respective countries. Timed tree of the 15 other clusters (Other): four clusters with a majority of patients of Suriname origin, three clusters with a majority of patients of Antillean origin, two clusters of heterosexually infected individuals and MSM of mainly Dutch origin, one cluster with mainly PWID of Polish origin, the largest PWID cluster, and the six clusters on Curaçao with bars (two are also in MSM). (ZIP) [file pmed.1001898.s014.zip › S1_Trees/MSM/MSMdc.pdf]

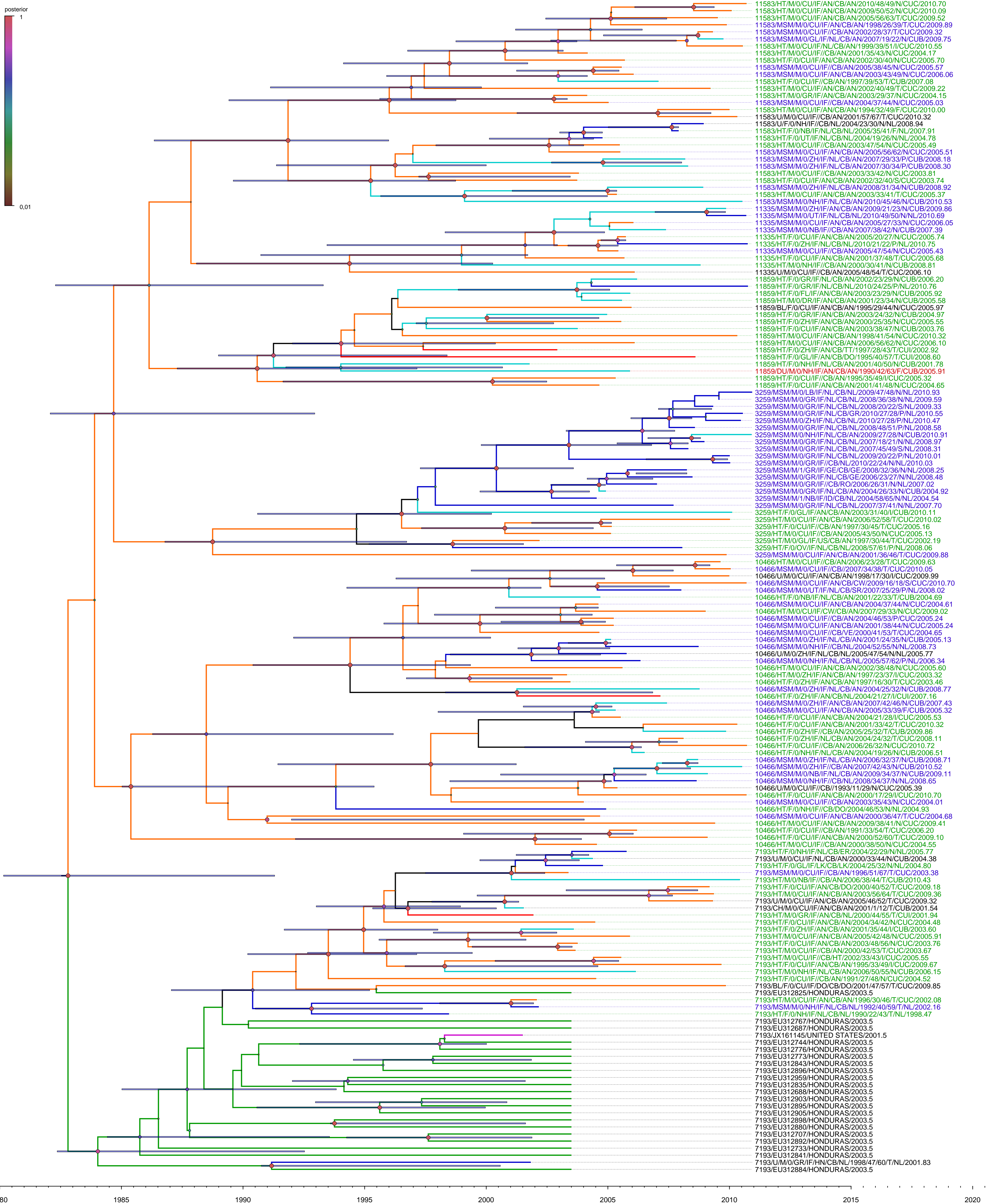

Supplement: S1 Trees — The first number of the branch name is the cluster name. Bars indicate the 95% HPD intervals of the node ages. In total, 380 of the Los Alamos sequences selected are included in the 91 large MSM-majority clusters. Twenty-six clusters have no Los Alamos sequences included. Nine clusters include ≥5 time-stamped sequences from a particular country (Foreign). Although some mixing is visible, these sequences form mainly separate clusters within the respective countries. Timed tree of the 15 other clusters (Other): four clusters with a majority of patients of Suriname origin, three clusters with a majority of patients of Antillean origin, two clusters of heterosexually infected individuals and MSM of mainly Dutch origin, one cluster with mainly PWID of Polish origin, the largest PWID cluster, and the six clusters on Curaçao with bars (two are also in MSM). (ZIP) [file pmed.1001898.s014.zip › S1_Trees/Other/Tree_CU_bars.pdf]

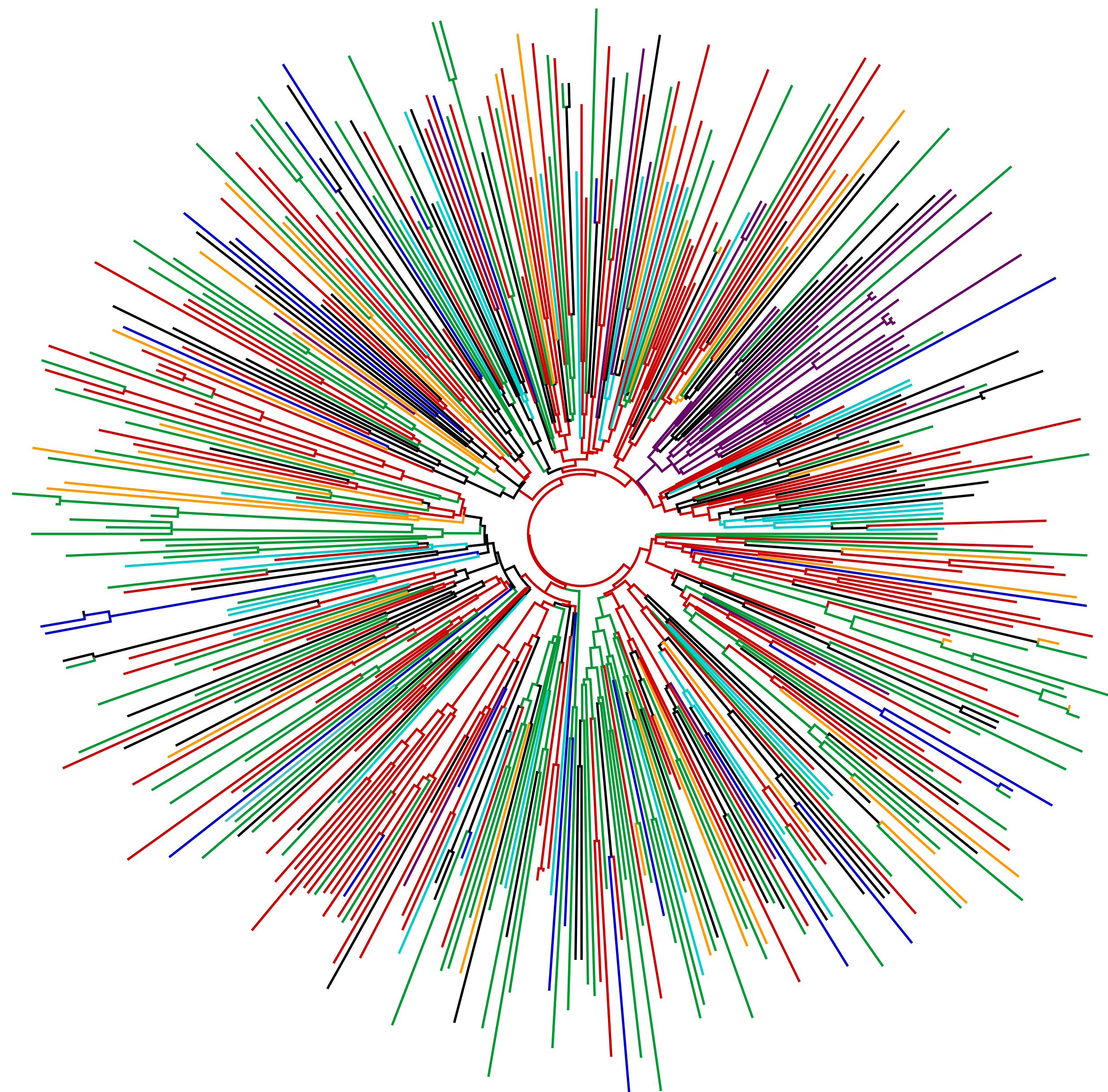

Transmission group:

- drug users
- unknown
- heterosexual
- Italy
- MSM
- USA
- other foreign

5 years

Supplement: S1 Trees — The first number of the branch name is the cluster name. Bars indicate the 95% HPD intervals of the node ages. In total, 380 of the Los Alamos sequences selected are included in the 91 large MSM-majority clusters. Twenty-six clusters have no Los Alamos sequences included. Nine clusters include ≥5 time-stamped sequences from a particular country (Foreign). Although some mixing is visible, these sequences form mainly separate clusters within the respective countries. Timed tree of the 15 other clusters (Other): four clusters with a majority of patients of Suriname origin, three clusters with a majority of patients of Antillean origin, two clusters of heterosexually infected individuals and MSM of mainly Dutch origin, one cluster with mainly PWID of Polish origin, the largest PWID cluster, and the six clusters on Curaçao with bars (two are also in MSM). (ZIP) [file pmed.1001898.s014.zip › S1_Trees/Other/Tree_Largestcluster_drugusers_rootprior.pdf]

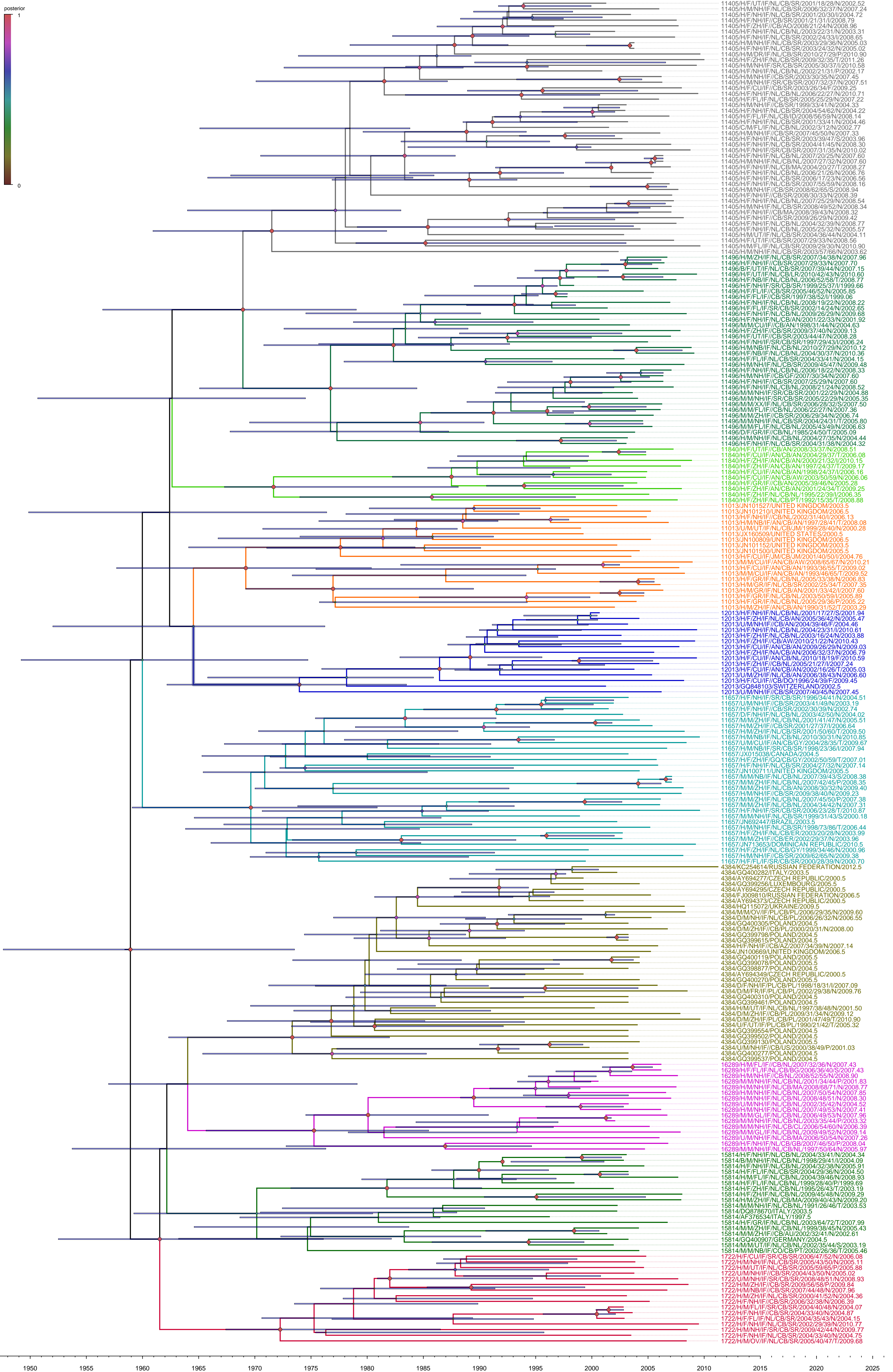

Supplement: S1 Trees — The first number of the branch name is the cluster name. Bars indicate the 95% HPD intervals of the node ages. In total, 380 of the Los Alamos sequences selected are included in the 91 large MSM-majority clusters. Twenty-six clusters have no Los Alamos sequences included. Nine clusters include ≥5 time-stamped sequences from a particular country (Foreign). Although some mixing is visible, these sequences form mainly separate clusters within the respective countries. Timed tree of the 15 other clusters (Other): four clusters with a majority of patients of Suriname origin, three clusters with a majority of patients of Antillean origin, two clusters of heterosexually infected individuals and MSM of mainly Dutch origin, one cluster with mainly PWID of Polish origin, the largest PWID cluster, and the six clusters on Curaçao with bars (two are also in MSM). (ZIP) [file pmed.1001898.s014.zip › S1_Trees/Other/Tree_other.pdf]

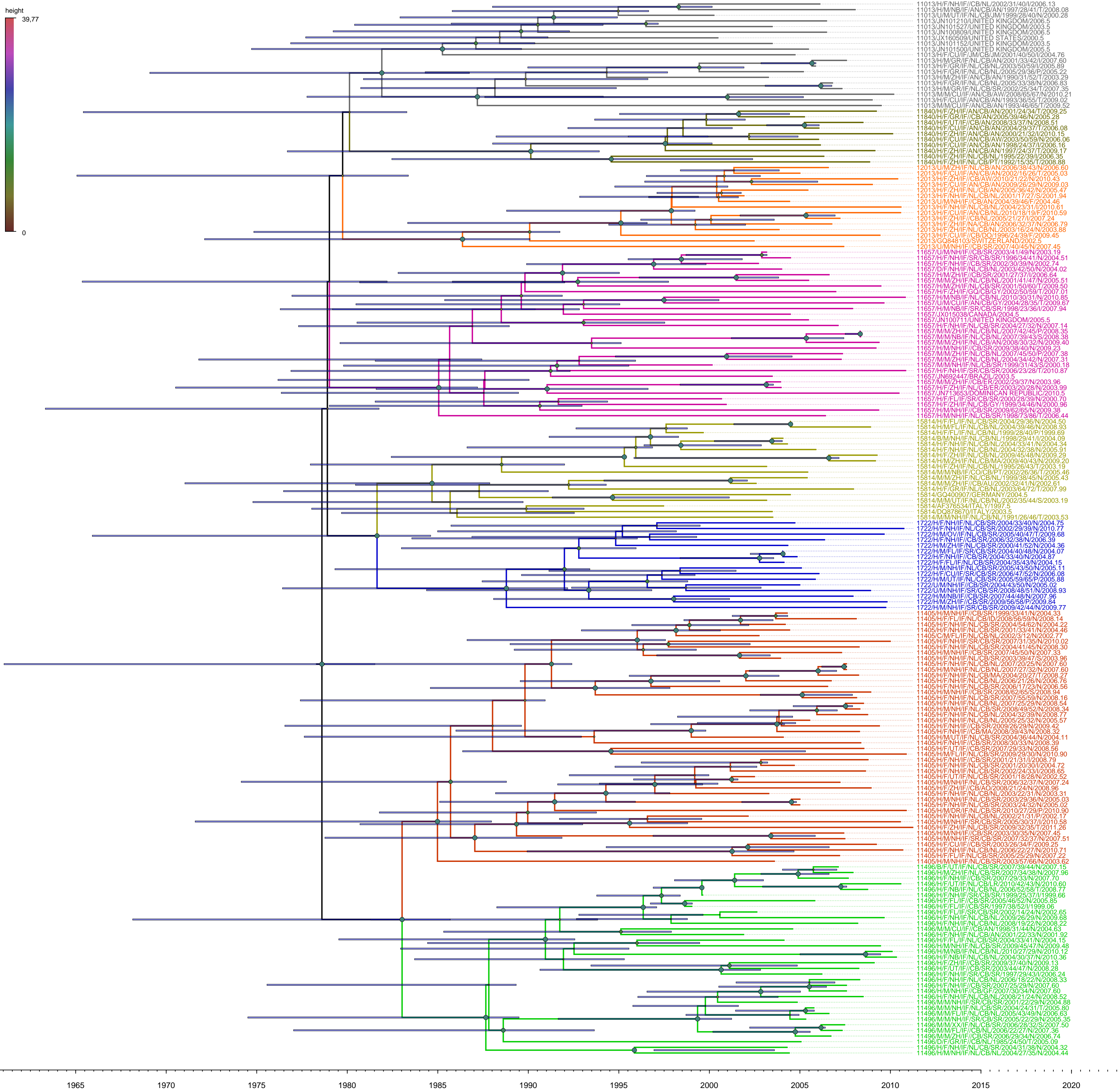

Supplement: S1 Trees — The first number of the branch name is the cluster name. Bars indicate the 95% HPD intervals of the node ages. In total, 380 of the Los Alamos sequences selected are included in the 91 large MSM-majority clusters. Twenty-six clusters have no Los Alamos sequences included. Nine clusters include ≥5 time-stamped sequences from a particular country (Foreign). Although some mixing is visible, these sequences form mainly separate clusters within the respective countries. Timed tree of the 15 other clusters (Other): four clusters with a majority of patients of Suriname origin, three clusters with a majority of patients of Antillean origin, two clusters of heterosexually infected individuals and MSM of mainly Dutch origin, one cluster with mainly PWID of Polish origin, the largest PWID cluster, and the six clusters on Curaçao with bars (two are also in MSM). (ZIP) [file pmed.1001898.s014.zip › S1_Trees/Other/Tree_SR.pdf]
