# Supplementary material for: Dispersion of the HIV-1 Epidemic in Men Who Have Sex with Men in the Netherlands: A Combined Mathematical Model and Phylogenetic Analysis
Source: PLoS Med. 2015 Nov 3;12(11):e1001898. doi: 10.1371/journal.pmed.1001898 (PMC4631366; doi:10.1371/journal.pmed.1001898)
Supplement: S9 Fig — (A) Branches are colored according to region of residence. (B) Branches are colored according to the self-reported risk group of infection. The root of each cluster is indicated in bold. HT, heterosexual transmission. (PDF) [file pmed.1001898.s009.pdf]

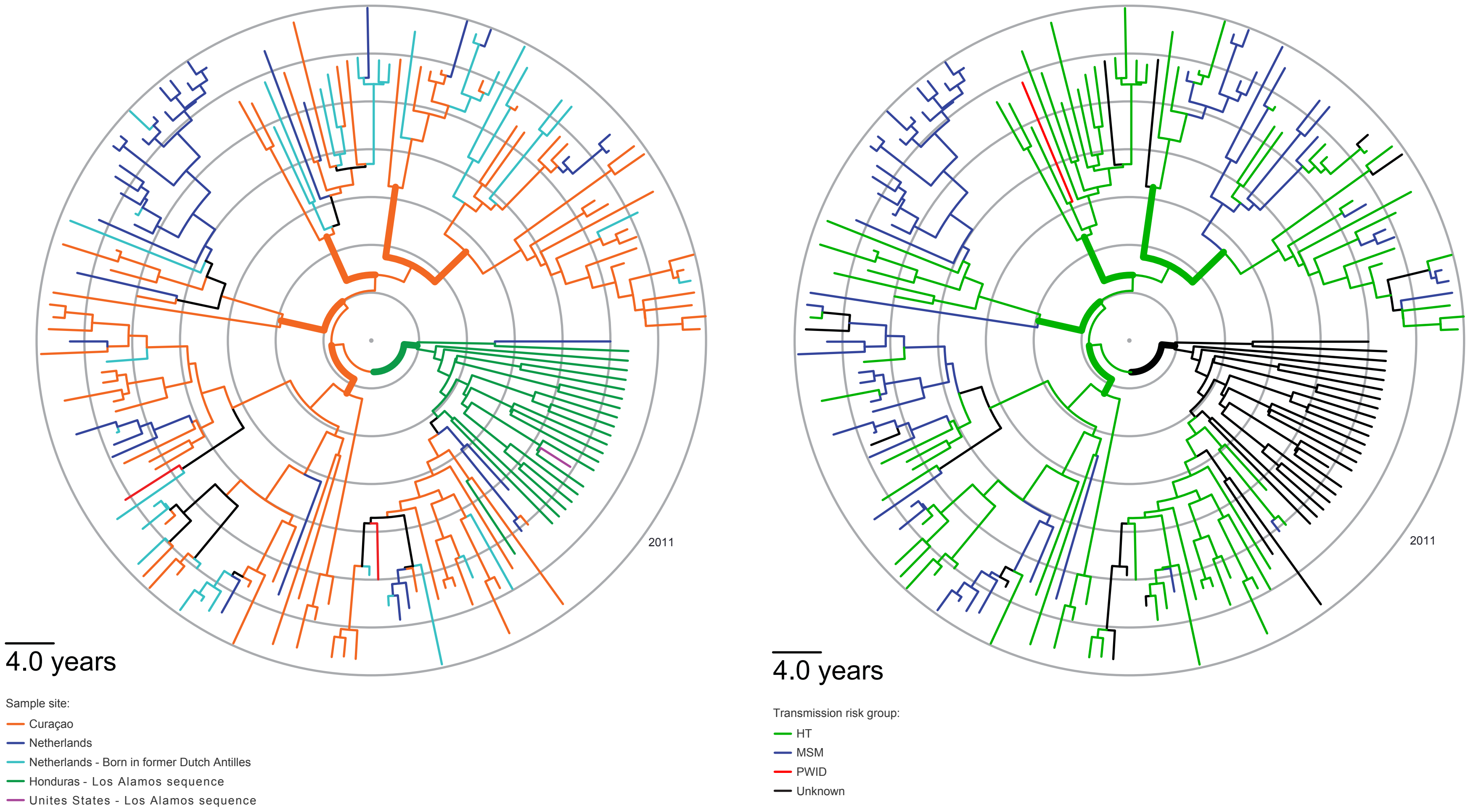
2011
4.0 years
Sample site:
Curaçao
Netherlands
Netherlands - Born in former Dutch Antilles
Honduras - Los Alamos sequence
Unites States - Los Alamos sequence
2011
4.0 years
Transmission risk group:
HT
MSM
PWID
Unknown
