# Supplementary material for: Dispersion of the HIV-1 Epidemic in Men Who Have Sex with Men in the Netherlands: A Combined Mathematical Model and Phylogenetic Analysis
Source: PLoS Med. 2015 Nov 3;12(11):e1001898. doi: 10.1371/journal.pmed.1001898 (PMC4631366; doi:10.1371/journal.pmed.1001898)
Supplement: S10 Fig — Under the Poisson (left) and geometric (right) model. The top three rows show how these proportions change as a function of π, the proportion of cases with a sequence, for a mean number of offspring of R = 1, R = 0.9, and R = 1.1, respectively. The vertical red dotted lines indicate the observed proportion of cases with a sequence, π = 0.3 over the whole time period considered. The bottom row shows how the proportions of singletons and small clusters change as a function of R, the mean number of offspring, for a proportion of cases with a sequence of π = 0.3. In all plots, the black and grey curves show the expected and observed proportion of singletons, respectively, with dotted lines indicating confidence intervals around the observed proportion; the dark and light blue curves show the expected and observed proportion of small clusters (size 2–9), with dashed lines indicating confidence intervals around the observed proportion. (PDF) [file pmed.1001898.s010.pdf]

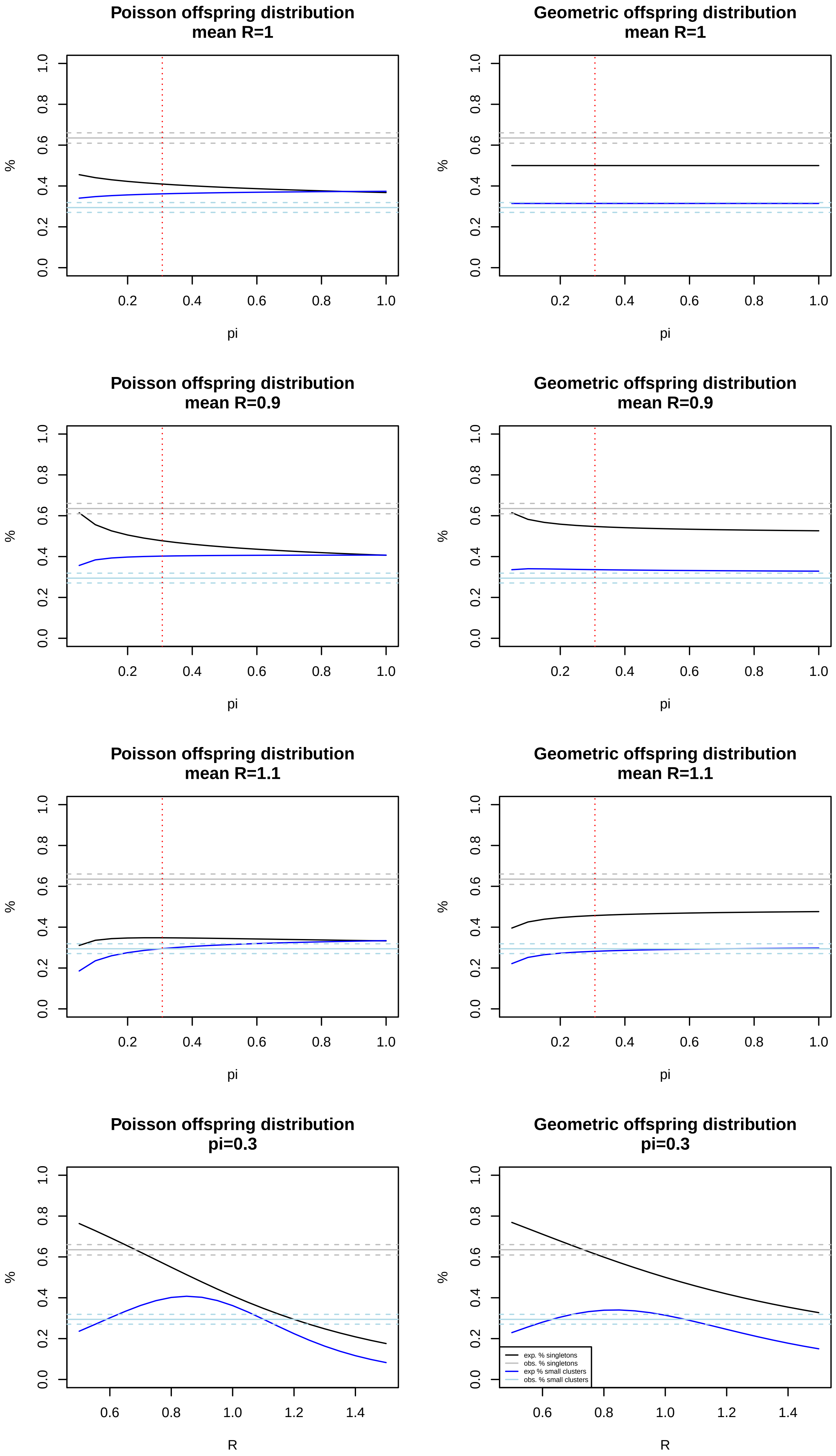
Poisson offspring distribution
mean R=1
Geometric offspring distribution
mean R=1
Poisson offspring distribution
mean R=0.9
Geometric offspring distribution
mean R=0.9
Poisson offspring distribution
mean R=1.1
Geometric offspring distribution
mean R=1.1
Poisson offspring distribution
pi=0.3
Geometric offspring distribution
pi=0.3
%
pi
R
0.0
0.2
0.4
0.6
0.8
1.0
1.2
1.4
exp. % singletons
obs. % singletons
exp % small clusters
obs. % small clusters
