# Supplementary material for: Dispersion of the HIV-1 Epidemic in Men Who Have Sex with Men in the Netherlands: A Combined Mathematical Model and Phylogenetic Analysis
Source: PLoS Med. 2015 Nov 3;12(11):e1001898. doi: 10.1371/journal.pmed.1001898 (PMC4631366; doi:10.1371/journal.pmed.1001898)
Supplement: S1 Table — To test whether HIV-1 pol sequences in the last 1,000 posterior trees obtained by BEAST were structured more strongly by risk group for the ATHENA sequences and more strongly by country for the sequences from the Los Alamos HIV Sequence Database [18] than expected by chance alone, a phylogenetic trait association analysis using Bayesian tip-association significance testing was performed [39]. The maximum monophyletic clade (MC) size statistic was estimated for each trait, which provides an estimate of the mean cluster size of sequences sampled for each trait. Significant clustering is defined as critical (p < 0.01), marginally significant (0.01 ≤ p ≤ 0.05), or not significant (p > 0.05). (PDF) [file pmed.1001898.s011.pdf]

**Risk group / Country**	**N**	**MC (95% HPD)**	**P-value**
UNITED STATES	38	7.1 (4-12)	**<0.001**
SPAIN	13	2.0 (2-2)	**0.018**
GERMANY	9	1.0 (1-1)	1
ITALY	91	2.0 (1-3)	0.253
SENEGAL	3	1.2 (1-2)	1
FRANCE	4	1 (1-1)	1
CANADA	2	1 (1-1)	1
ARGENTINA	7	2.0 (2-2)	**0.003**
CZECH REPUBLIC	1	1 (1-1)	1
PWID	136	6.2 (6-7)	**0.004**
IRELAND	1	1 (1-1)	1
BELGIUM	2	1 (1-1)	1
DENMARK	1	1 (1-1)	1
AUSTRIA	1	1 (1-1)	1
SWITZERLAND	10	2.0 (2-2)	**0.015**
UNITED KINGDOM	12	2.6 (2-3)	**<0.001**
MSM	24	1.7 (1-2)	0.105
Heterosexual	122	(8-9)	**<0.001**
