# Supplementary material for: Dispersion of the HIV-1 Epidemic in Men Who Have Sex with Men in the Netherlands: A Combined Mathematical Model and Phylogenetic Analysis
Source: PLoS Med. 2015 Nov 3;12(11):e1001898. doi: 10.1371/journal.pmed.1001898 (PMC4631366; doi:10.1371/journal.pmed.1001898)
Supplement: S1 Text — (PDF) [file pmed.1001898.s013.pdf]

Dispersion of the HIV-1 Epidemic in Men Who Have Sex with Men in the Netherlands: A Combined Mathematical Model and Phylogenetic Analysis

Daniela Bezemer[1☯]*, Anne Cori[2☯], Oliver Ratmann[2], Ard van Sighem[1], Hillegonda S. Hermanides[3], Bas E. Dutilh[4,5,6], Luuk Gras[1], Nuno Rodrigues Faria[7], Rob van den Hengel[1], Ashley J. Duits[3], Peter Reiss[1,8,9], Frank de Wolf[2], Christophe Fraser[2], and the ATHENA observational cohort[¶]

1 HIV Monitoring Foundation, Amsterdam, the Netherlands,

2 Medical Research Council Centre for Outbreak Analysis and Modelling, Department of Infectious Disease Epidemiology, School of Public Health, Imperial College London, London, United Kingdom,

3 Red Cross Blood Bank Foundation, Willemstad, Curaçao,

4 Centre for Molecular and Biomolecular Informatics, Nijmegen Centre for Molecular Life Sciences, Radboud University Medical Centre, Nijmegen, the Netherlands

5 Department of Marine Biology, Institute of Biology, Federal University of Rio de Janeiro, Rio de Janeiro, Brazil,

6 Theoretical Biology and Bioinformatics, Utrecht University, Utrecht, the Netherlands,

7 Department of Zoology, University of Oxford, Oxford, United Kingdom,

8 Department of Global Health, Academic Medical Center, Amsterdam, the Netherlands,

9 Amsterdam Institute for Global Health and Development, Amsterdam, the Netherlands

***** d.o.bezemer@amc.uva.nl

☯ These authors contributed equally to this work.

SUPPLEMENTARY INFORMATION

CONTENTS

ANALYSIS ON R_t

Page 3 Distribution of the diagnosis interval

Page 4 Derivation of q_i and its impact on the estimates of the reproduction numbers

Page 5 Analyses of a truncated dataset to assess the robustness of estimates of R_t

Page 5 Comparison of R_t over different time periods

Page 5 Estimate the rate of increase in the mean estimated R_t

PHYLOGENETIC ANALYSIS

Page 6 Non-MSM majority transmission clusters

Page 6 PWID - Largest cluster

Page 7 Transmission clusters circulating on Curaçao

ANALYSIS OF CLUSTER SIZES

Page 8

References

Page 12

SUPPLEMENTARY INFORMATION - ANALYSIS ON R_t

Distribution of the diagnosis interval

The time varying diagnosis interval was obtained by numerically simulating a compartmental HIV transmission model previously fitted to HIV and AIDS diagnosis data on MSM in the Netherlands, where it is assumed no transmission occurs when individuals are on treatment [1]. We considered a simplified version of this model, where we neglect treatment failure since this contributes little to the diagnosis interval. Once infected, individuals go through the primary infection stage, followed by five stages of infection. At any of those 5 stages, individuals can be diagnosed, after which they progress through some of 5 mirroring diagnosed stages, in any of which they can initiate treatment. Transition rates as well as relative infectivity of the different stages were taken from Bezemer et al. [2]. For each year between 1975 and 2014, we simulated the trajectory of 100 cases infected that year, as well as the trajectory of their secondary cases. For all pairs of index-secondary cases, we recorded the time between diagnosis of the index case and diagnosis of the secondary case, and stratified these according to the date of diagnosis of the index case, leading to a numerical approximation of the diagnosis interval distribution $w_t(.)$, with a lower bound -S = -20 years (see S1 Fig).

Note that our extension of the Wallinga and Teunis method, which allows for negative diagnoses intervals, may allow "cycles", whereby A infects B and B infects A. However, the aim of the Wallinga and Teunis method, and of the extension we propose here, is not to reconstruct who infected whom per se, but rather to assess how many secondary cases, on average, a case infected or diagnosed at a given time will produce. In that sense, although p_{ij} is described as the relative probability that i infects j, it should in fact be regarded as the relative probability that any case diagnosed the same year as i infects j (or any case infected the same year as j). This view highlights the fact that this method is only applicable to large networks. In that case, the cycle issue A infects B infects A translates into "someone diagnosed the same year as A infects B and someone diagnosed same year as B infects A", which is not an issue.

Another important feature of our analysis is that we have assumed homogeneity across clusters both in terms of the proportion of individuals in each cluster for which a sequence was taken

(which was assumed to vary over time but not across clusters), and in terms of timing of HIV transmission (the diagnosis interval derived from Bezemer et al. [2] was assumed to vary over time but to be homogeneous across clusters).

Derivation of q_i and its impact on the estimates of the reproduction numbers

We defined q_i as the likelihood that case i has been infected by a case observed up to year T.

This is equal to $q_i = \frac{\sum_{t=0}^{T} R(t) I(t) w_t(t_i - t)}{\sum_{t=0}^{+\infty} R(t) I(t) w_t(t_i - t)}$, where R is the effective reproduction number and I is the incidence of diagnoses. In this formula, $R(t) I(t) w_t(t_i - t)$ is the average number of secondary cases diagnosed at time t_i who were generated by the $I(t)$ individuals diagnosed at time t. The numerator is therefore the average number of secondary cases diagnosed at time t_i who are generated by cases diagnosed up to time T, whereas the denominator is the overall average number of secondary cases diagnosed at time t_i.

We approximated this quantity by: $q_i = \frac{\sum_{t=0}^{T} w_t(t_i - t)}{\sum_{t=0}^{+\infty} w_t(t_i - t)}$, which assumes that the product RI is constant over time. Here, we assess how q_i is affected by this assumption. We considered 4 scenarios in which this product is exponentially increasing, with doubling times 1, 5, 10 and 20 years, corresponding to growth rates of 0.69, 0.14, 0.069 and 0.035 per year. We compared these to a scenario with constant RI (growth rate 0) and a scenario with exponentially decaying RI, with a growth rate of -0.035 per year. The corresponding q_i's are shown in S2 Fig.

In scenarios where incidence is increasing over time, our derivation of q_i under the constant incidence assumption is an underestimate of the true q_i in recent years. This leads us to underestimate $p_{ij} = p_{ij}^{*} \times q_i$ (the relative probability that case i has been infected by case j) when individual i is infected in recent years. S3 Fig shows how this affects our yearly estimates of R for the four largest transmission clusters. Interestingly, although the estimated values of R are sensitive to our assumptions, the temporal trends in R are very robust.

Analyses of a truncated dataset to assess the robustness of estimates of R_t

To further assess the robustness of our estimates of the reproduction number, we repeated our estimation procedure, but truncating the data after 2000. We then compared the estimates of the reproduction number obtained using the truncated and the full dataset (S4 Fig). Note that the estimates of R_t in 2000 based on the full dataset appear to be closer to the threshold value 1 than those based on the truncated dataset. Out of the 15 clusters which could be compared, four (panels A-D) had estimates based on both truncated and non-truncated datasets below 1, five (panels E-I) had both estimates above 1, and 6 (panels J-O) had estimates above 1 based on the truncated dataset and below 1 based on the full dataset. However, for these 6 clusters, both credible intervals included the threshold value 1. Importantly, for these 6 clusters, the overall trend in transmissibility around 2000 were not dramatically different based on the truncated and the full dataset.

Comparison of R_t over different time periods

We performed a paired t-test to compare R_t in three different time periods of our estimation: period 1, <1996; period 2, 1996-2001; and period 3, >2001). S5 Fig shows the comparison of R_t in different time periods. There was a significant but small decrease in the mean R_t between periods 1 and 2 (mean decrease of 0.090 $p<0.005$), and no significant change between time periods 2 and 3.

Estimate the rate of increase in the mean estimated R_t

We investigated if recently introduced clusters have reproductive numbers that differ from the older clusters when they were new, to see if R_t might be higher early after founding of a network. We reran our estimation procedure to derive estimates of R_t over the first 5 years for each cluster. Results are shown in S6 Fig. We used a linear regression to estimate the rate of increase in the mean estimated R_t as a function of the year the cluster appeared (defined as the year of the earliest diagnosis for this cluster). R_t increased by 0.018 per year (adjusted R^2=0.66). These results confirm our main findings that transmissibility in recent clusters is greater than it was in older clusters.

SUPPLEMENTARY INFORMATION - PHYLOGENETIC ANALYSIS

Non-MSM majority transmission clusters

From the phylogenetic tree of 8,320 HIV-1 subtype B *polymerase* sequences in total (S7 Fig), 106 large transmission clusters were identified that included sequences from ≥10 patients from the ATHENA cohort. Fifteen of the 106 clusters were not MSM majority clusters. Six of these 15 large clusters were dominated by sequences from patients living or born on the (former) Dutch Antilles. One cluster was a mixed Latin Caribbean cluster including also 5 'Los Alamos' sequences from the UK, and another one was the largest cluster in this study, containing 66% of all 207 sequences from drug-users in our study. Four clusters together included sequences from 69 heterosexually infected individuals (and 6 MSM) of Surinam origin, of which one also included 3 sequences from patients sampled in Suriname. Together, these four clusters included 43% of the 159 sequences from heterosexually infected individuals born in Suriname. Another cluster was found to include sequences from 6 persons who injected drugs (PWID) and 1 MSM, all of Polish origin, and also included Los Alamos sequences, 9 from Czech republic and 14 from Poland. This cluster included 30% of sequences from the Polish born people in this study, and all PWID born in Poland. Two other clusters had a mix of sequences from MSM and heterosexually infected individuals. Of the sequences from heterosexually infected patients in the Netherlands, 19% (194) were in 64 large MSM-dominated clusters, of these patients 129 (66%) were men, of whom 77% had a Dutch origin.

PWID - Largest cluster

Only eight (4%) sequences from PWID were in 5 large MSM majority clusters. Overall, 66% (136) of all sequences from PWID in this study were part of the largest cluster found in this study. This cluster consisted of in total 327 ATHENA sequences: 42% (136) sequences from PWID, 37% (122) from heterosexually infected individuals, and only 7% (24) from MSM. The earliest diagnosis in the cluster was a PWID in 1982. The latest recent infection through drug use was in 1999. The timed phylogenetic tree showed no evidence of transmission amongst PWID since 2000, i.e. the latest time point of the common ancestor of any two sequences from PWID. In 2010, the last year of this study, 4 heterosexually infected individuals, 2 PWID and one MSM were diagnosed in this cluster. The cluster also included 200 sequences from the Los Alamos

comparison, of whom 91 from Italy and 38 from the United states. S1 table shows the results of Bayesian Tip-association Significance testing (BaTS analysis) [2]. Significant clustering was found within PWID and heterosexual risk groups, but not amongst the MSM in this cluster. The sequences from the United States significantly clustered together at the root of the tree, whilst the Italian sequences were dispersed throughout the tree.

Transmission clusters circulating on Curaçao

Six clusters included ≥5 sequences from patients registered on Curaçao, a Caribbean island within the kingdom of the Netherlands. All 6 clusters included sequences from patients in the Netherlands and consisted of ≥10 sequences. The 6 clusters were confirmed by visual inspection of the whole phylogenetic tree and in a dated phylogenetic tree (S9 Fig). In total, these six clusters included 33% (73) of sequences from 219 patients on the island. 35% (18) of MSM and 33% (48) of heterosexually infected patients with a sequence in this study. Diversification by risk group is visible. In total, the 6 clusters included sequences from 71 patients in the Netherlands, of whom 48% were born in the former Dutch Antilles. Diversification can be seen into MSM clusters within the Netherlands (two majority MSM). Besides these 6 large clusters, 51 sequences from patients on Curaçao were identified as singletons (17%), 58 sequences were in 39 smaller clusters, and 37 sequences (14%) were in 23 clusters in the Netherlands. In total, this adds up to 119 (54%) clusters on the island, of which 5% (6) identified as on-going established transmission clusters.

SUPPLEMENTARY INFORMATION - ANALYSIS OF CLUSTER SIZES

We wanted to assess whether the observed proportion of singletons, or of small clusters (size 2-9) was consistent with branching process theory. We considered the total "cluster" produced by each imported case. The distribution of the total cluster size can be analytically derived for certain offspring distributions, i.e. the distribution of number of secondary cases generated by each case. Farrington et al. (Biostatistics 2003) show that the distribution of the total cluster size, X, for an offspring distribution with mean R is (after simplification):

$P(X = x|R, Pois) = \frac{x^{x-2} R^{x-1} e^{-Rx}}{\Gamma(x)}$ if the offspring distribution is Poisson, and

$P(X = x|R, Geom) = \frac{\Gamma(2x-1)}{\Gamma(x)\Gamma(x+1)} \frac{R^{x-1}}{(1+R)^{2x-1}}$ if the offspring distribution is Geometric.

This allows in particular evaluating the expected true proportion of singletons, or of small clusters (size 2-9) amongst all clusters, by computing $P(X = 1|R, Model)$ and $\sum_{x=2}^{9} P(X = x|R, Model)$ respectively.

Let's now assume that only a proportion π of cases are observed, and denote Y the observed cluster size. The distribution of Y given X is given by a binomial distribution:

$$P(Y = y|X = x, \pi) = \binom{x}{y} \pi^y (1-\pi)^{x-y}$$

Therefore, the distribution of Y, given an offspring distribution $Model$ with mean R can be written as: $P(Y = y|R, \pi, Model) = \sum_{x \geq 1} P(Y = y|X = x)\, P(X = x|R, Model)$

and the expected observed proportion of singletons can be calculated as:

$$P(Y = 1|Y \geq 1, R, \pi, Model) = \frac{P(Y = 1|R, \pi, Model)}{P(Y \geq 1|R, \pi, Model)} = \frac{P(Y = 1|R, \pi, Model)}{1 - P(Y = 0|R, \pi, Model)}$$
$$= \frac{\sum_{x \geq 1} P(Y = 1|X = x, \pi)\, P(X = x|R, Model)}{1 - \sum_{x \geq 1} P(Y = 0|X = x, \pi)\, P(X = x|R, Model)}$$

and the proportion of small clusters can be derived in a similar manner.

S10 Fig shows how the expected proportion of observed singletons or small clusters varies according to the proportion of cases sampled, π, and the mean offspring R, under the Poisson and the Geometric offspring distributions. Interestingly, under the Geometric model, when $R = 1$, the expected proportion of observed singletons or small clusters is independent on the sampling fraction π (see below for an analytical proof for the singletons). The figure also shows the proportion of singletons and small clusters observed in our analysis. The observed proportion of small clusters is well in line with the expected proportion under a geometric model with mean offspring $R = 1$. The observed proportion of singletons, on the other hand, is higher than expected under this model.

However, the branching process model with random sampling described above doesn't capture the following feature of our data. As the sampling fraction is not complete, we may be unable to detect that two cases with a sequence belong to the same cluster, as sequences from intermediate cases might have been needed to identify these belonging to the same transmission cluster. We may also be unable to detect that two cases with a sequence belong to the same cluster if one or both of these cases is multiply infected, or had a sample sequenced at an advanced stage of infection. Therefore the observed proportion of singletons may be higher than expected under the model described above, precisely because we may be unable to merge some of the singletons with larger clusters based only on a partial and imperfect sample of sequences.

Another element to note is that the theoretical derivation considers the final size of the outbreak generated by each importation, whereas the data captures the size of each cluster at a point in time where the clusters might still be ongoing. Therefore observed clusters are smaller than expected, because we may not have observed them until they die out.

Overall, we conclude that, although a simple branching model with random sampling is unable to fully capture the complexity of our data, the model with geometric offspring distribution with $R = 1$ or slightly lower is reasonably well suited to describe our data. Under this model, the expected proportion of singletons and of small clusters is independent on the sampling fraction, so that the true proportion of singletons and small clusters is similar to the observed one despite partial sampling. Our observations show that 64% of clusters are singletons, and 29% are small (size 2-9), whilst the geometric model with mean offspring $R = 1$ leads to 50% of singletons and 31% of small clusters. This suggests that amongst all imported cases, only a small fraction (7%

according to the observations, and 19% according to the model) will go on and establish a large cluster (i.e. 10 cases or more).

Proof that the observed proportion of singletons is independent of the sampling fraction under the geometric model with *R*=1.

The observed proportion of singletons is

$P(Y=1|Y\geq 1,R,\pi,Geom)=\frac{\sum_{x\geq 1}P(Y=1|X=x,\pi)P(X=x|R,Geom)}{1-\sum_{x\geq 1}P(Y=0|X=x,\pi)P(X=x|R,Geom)}$, with $P(Y=y|X=x,\pi)=\binom{x}{y}\pi^{y}(1-\pi)^{x-y}$ and $P(X=x|R,Geom)=\frac{\Gamma(2x-1)}{\Gamma(x)\Gamma(x+1)}\frac{R^{x-1}}{(1+R)^{2x-1}}$.

We want to show that $(Y=1|Y\geq 1,R,\pi,Geom)$ is independent of π.

First, let's simplify the numerator:

$\sum_{x\geq 1}P(Y=1|X=x,\pi)\,P(X=x|R,Geom)=\sum_{x\geq 1}x\pi(1-\pi)^{x-1}\frac{\Gamma(2x-1)}{\Gamma(x)\Gamma(x+1)}\frac{R^{x-1}}{(1+R)^{2x-1}}=$
$\frac{\pi(1+R)}{(1-\pi)R}\sum_{x\geq 1}\frac{\Gamma(2x-1)}{\Gamma(x)^2}\left(\frac{R(1-\pi)}{(1+R)^2}\right)^{x}=\frac{\pi}{(1+R)}\sum_{x\geq 1}\binom{2x-2}{x-1}\left(\frac{R(1-\pi)}{(1+R)^2}\right)^{x-1}=$
$\frac{\pi}{(1+R)}\sum_{x\geq 0}\binom{2x}{x}\left(\sqrt{\frac{R(1-\pi)}{(1+R)^2}}\right)^{2x}=\frac{\pi}{(1+R)}\frac{1}{\sqrt{1-4\frac{R(1-\pi)}{(1+R)^2}}}$, the last equality being derived from the power series: $\sum_{n\geq 0}\frac{(2n)!}{2^{2n}(n!)^2}z^{2n}=\frac{1}{\sqrt{1-z^2}}$.

When $R=1$, this simplifies to $\sum_{x\geq 1}P(Y=1|X=x,\pi)\,P(X=x|R=1,Geom)=\frac{\sqrt{\pi}}{2}$

Now, let's simplify the denominator:

1-$\sum_{x\geq 1} P(Y=0|X=x,\pi)\, P(X=x|R,Geom) = 1-\sum_{x\geq 1}(1-\pi)^x \frac{\Gamma(2x-1)}{\Gamma(x)\Gamma(x+1)} \frac{R^{x-1}}{(1+R)^{2x-1}} = 1-$

$\frac{1+R}{R}\sum_{x\geq 1}\frac{\Gamma(2x-1)}{\Gamma(x)\Gamma(x+1)}\left(\frac{R(1-\pi)}{(1+R)^2}\right)^x = 1-\frac{1+R}{2R}\sqrt{\frac{R(1-\pi)}{(1+R)^2}}\sum_{x\geq 1}\binom{2x}{x}\frac{1}{2x-1}\left(\sqrt{\frac{R(1-\pi)}{(1+R)^2}}\right)^{2x-1} = 1-$

$\frac{1+R}{2R}Af(A)$

with $A=\sqrt{\frac{R(1-\pi)}{(1+R)^2}}$ and $f(A)=\sum_{x\geq 1}\binom{2x}{x}\frac{1}{2x-1}A^{2x-1}$.

The differentiation of this power series leads to (after some manipulation and using again $\sum_{n\geq 0}\frac{(2n)!}{2^{2n}(n!)^2}z^{2n}=\frac{1}{\sqrt{1-z^2}}$.):

$f'(A)=\frac{1-\sqrt{1-4A^2}}{A^2\sqrt{1-4A^2}}$, wich integrates back to $f(A)=\frac{1-\sqrt{1-4A^2}}{A}$ (using $f(0)=0$).

Therefore, $\sum_{x\geq 1} P(Y=0|X=x,\pi)\, P(X=x|R,Geom) = 1-\frac{1+R}{2R}\left(1-\sqrt{1-4A^2}\right)$.

When $R=1$, $A=\frac{\sqrt{1-\pi}}{2}$ and this simplifies to $\sum_{x\geq 1} P(Y=0|X=x,\pi)\, P(X=x|R=1,Geom)=\sqrt{\pi}$.

Therefore in this specific case $P(Y=1|Y\geq 1,R=1,\pi,Geom)=\frac{\frac{\sqrt{\pi}}{2}}{\sqrt{\pi}}=\frac{1}{2}$, which is independent of π. In particular the observed proportion of singletons for any value of π is the same as the true proportion of singletons if all cases are observed (i.e. for $\pi=1$).

References

1. Bezemer D, de Wolf F, Boerlijst MC, vanSighem A, Hollingsworth TD, Fraser C (2010) 27 years of the HIV epidemic amongst men having sex with men in the Netherlands: an in depth mathematical model-based analysis. Epidemics 2: 66-79. S1755-4365(10)00030-7 [pii];10.1016/j.epidem.2010.04.001 [doi].

2. Parker J, Rambaut A, Pybus OG (2008) Correlating viral phenotypes with phylogeny: accounting for phylogenetic uncertainty. Infect Genet Evol 8: 239-246.

3. Los Alamos National Laboratory (2015) HIV Sequence Database [database]. http://www.hiv.lanl.gov/content/sequence/HIV/mainpage.html
